# Fate of hepatitis D virus-specific CD8+ T cells during bulevirtide monotherapy in patients with chronic hepatitis delta^☆^

**DOI:** 10.1016/j.jhepr.2026.101970

**Published:** 2026-07-24

**Authors:** Valerie Oberhardt, Elisabetta Degasperi, Michelle Maas, Marta Borghi, Kathrin Heim, Roberta Soffredini, Maria Paola Anolli, Floriana Facchetti, Stéphanie Narguet, Tarik Asselah, Elahe Salimi Alizei, Özlem Sogukpinar, Frances Winkler, Sian Llewellyn-Lacey, Florian Emmerich, David A. Price, Tobias Boettler, Bertram Bengsch, Hendrik Luxenburger, Maike Hofmann, Robert Thimme, Pietro Lampertico, Christoph Neumann-Haefelin

**Affiliations:** 1 Department of Medicine II, Freiburg University Medical Center, Faculty of Medicine, University of Freiburg, Freiburg, Germany; 2 Division of Gastroenterology and Hepatology, Foundation IRCCS Ca’ Granda Ospedale Maggiore Policlinico, Milan, Italy; 3 Faculty of Biology, University of Freiburg, Freiburg, Germany; 4 Department of Gastroenterology and Hepatology, University Hospital Cologne, Faculty of Medicine, University of Cologne, Cologne, Germany; 5 University of Paris-Cité, Hôpital Beaujon, Service d'hépatologie AP-HP & INSERM UMR1149, Clichy, France; 6 Division of Infection and Immunity, Cardiff University School of Medicine, University Hospital of Wales, Cardiff, UK; 7 Institute for Transfusion Medicine and Gene Therapy, Freiburg University Medical Center, Faculty of Medicine, University of Freiburg, Freiburg, Germany; 8 Systems Immunity Research Institute, Cardiff University School of Medicine, University Hospital of Wales, Cardiff, UK; 9 Signalling Research Centres BIOSS and CIBSS, University of Freiburg, Freiburg, Germany; 10 CRC “A. M. and A. Migliavacca” Center for Liver Disease, Department of Pathophysiology and Transplantation, University of Milan, Milan, Italy; 11 D-SOLVE consortium, an EU Horizon Europe funded project (No 101057917), Hannover, Germany; 12 German Centre for Infection Research (DZIF), Site Bonn-Cologne, Cologne, Germany; 13 Center for Molecular Medicine Cologne (CMMC), University of Cologne, Cologne, Germany

**Keywords:** Hepatitis D virus, Hepatitis delta, Virus-specific CD8+ T cell, Viral escape, Bulevirtide

## Abstract

**Background & Aims:**

Bulevirtide reduces viremia in chronic hepatitis D virus (HDV) infection, but long-term treatment is required in most patients to prevent relapse. Sustained treatment responses may be fostered by therapy-induced amelioration of HDV-specific CD8+ T-cell responses that are exhausted because of high viremia and antigen loads during chronic HDV infection. We therefore studied the effect of bulevirtide monotherapy on HDV-specific CD8+ T-cell repertoire, phenotype, and functionality.

**Methods:**

Twenty-eight patients with HDV infection and cirrhosis starting bulevirtide treatment were followed for 40–120 weeks on treatment. HLA class I typing and HDV sequencing was performed. HDV-specific CD8+ T cells were analyzed longitudinally using optimal epitopes and overlapping peptides spanning the L-HDAg. *Ex vivo* high-dimensional flow cytometry analysis of peptide/HLA class I tetramer+ CD8+ T cells was performed longitudinally in selected patients.

**Results:**

In 42% of patients, an HDV-specific CD8+ T-cell response was detectable at baseline, but did not substantially increase during treatment. Importantly, a large majority of HDV-specific CD8+ T-cell responses targeted viral epitopes with sequence variations consistent with viral escape mutations. Only one HLA-B∗35-restricted HDV-specific CD8+ T-cell epitope was conserved and continuously targeted throughout therapy. HDV-specific CD8+ T cells targeting this conserved epitope displayed a predominant terminally exhausted phenotype at baseline that shifted longitudinally to a memory-like phenotype with suppression of HDV load.

**Conclusions:**

Albeit based on small patient numbers, antiviral treatment with bulevirtide could ameliorate exhausted HDV-specific CD8+ T cells targeting conserved HDV epitopes. However, as the majority of HDV-specific CD8+ T-cell responses target escaped viral epitopes, this does not translate to a substantial improvement in overall HDV-specific CD8+ T-cell immunity. Our findings may explain in part why long-term bulevirtide treatment is required to prevent viral relapse.

**Impact and implications:**

Bulevirtide can control viremia and normalize liver enzymes in a majority of patients with chronic hepatitis delta, but sustained cure with negative HDV RNA even after therapy is difficult to achieve. Restoration of functional HDV-specific CD8+ T cells attributable to reduced antigen loads may prevent viral relapse. We demonstrate here that amelioration of HDV-specific CD8+ T cells does indeed occur, but is limited to the few HDV-specific CD8+ T-cell responses targeting conserved viral epitopes. A majority of HDV-specific CD8+ T cells no longer recognize their target antigens because of mutational viral escape. These findings explain the need for long-term treatment continuation even once HDV RNA is negative, to prevent viral relapse.

## Highlights

• During bulevirtide treatment, HDV-specific CD8+ T cells targeting conserved HDV epitopes are phenotypically ameliorated during antigen load reduction.
• However, the majority of HDV-specific CD8+ T-cell responses target viral epitopes with mutational viral escape.
• These results suggest that HDV-specific T cells may not contribute to prevent HDV relapse after bulevirtide discontinuation.
• Thus, long-term treatment may be needed even after achievement of HDV RNA negativity.

## Graphical abstract

**Figure:**
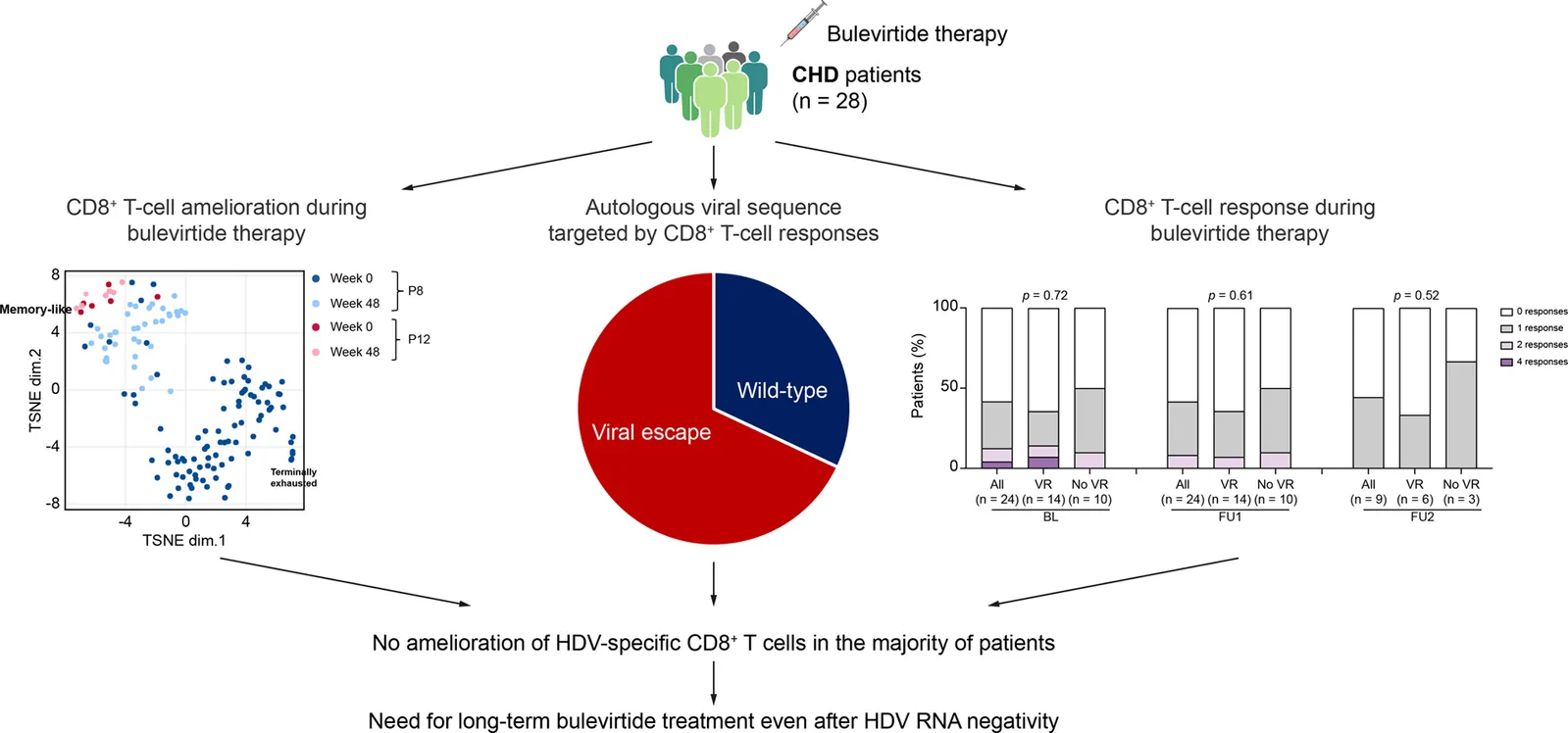

## Introduction

HDV is a small defective hepatotropic RNA virus that depends on the HBsAg for cell entry into hepatocytes. HDV super-infection of individuals who are HBV-positive mostly results in chronic hepatitis delta, leading to cirrhosis in ∼75% of patients within 15 years.^1^ Until 2020, off-label pegylated interferon-alpha (pegIFNα) was the only therapeutic option for patients with chronic hepatitis delta. Treatment with pegIFNα, however, results in sustained virological response in only ∼20% and is contraindicated in many patients, including those with advanced cirrhosis. In 2020, the entry inhibitor bulevirtide (BLV), a peptide homologue of the N-terminal HBsAg domain preS1, was approved for the treatment of chronic hepatitis delta in Europe. BLV leads to a decline of HDV viremia as well as normalization of liver enzymes in the majority of patients. Indeed, ∼70% of treated patients display a virological response, defined as an at least two log decline of HDV RNA, and a biochemical response, defined as normalization of alanine aminotransferase (ALT) levels, respectively, resulting in a combined response (virological + biochemical response) in ∼50% of patients.[2], [3], [4] While treatment response continues to increase with treatment duration, only a smaller part of patients become completely negative for HDV RNA after a treatment duration of up to 3 years, and even these patients remain at high risk of viral relapse after stopping BLV therapy.^5^ Thus, long-term treatment with BLV is the current state of the art.^6^

In chronic hepatitis delta, HDV-specific CD8+ T cells are detectable in a subset of patients. Indeed, two studies using overlapping peptides as well as one study mapping HDV-specific CD8+ T cell epitopes in regions with HLA class I-associated sequence variations identified HDV-specific CD8+ T-cell responses in 38.9–70.6% of patients.[7], [8], [9] The HDV-specific CD8+ T cell epitopes were largely restricted by HLA-B alleles. Mutational viral escape was observed in a substantial number of epitopes; however, the proportion of HDV-specific CD8+ T-cell responses affected by viral escape has not been defined.^7^^,^^8^ Of note, HDV-specific CD8+ T cells targeting wild-type epitopes displayed a ‘chronically activated’ or exhausted phenotype.[7], [8], [9], [10] As T-cell exhaustion is thought to be mainly driven by high antigen loads, BLV-induced decline of HDV viremia may result in amelioration of HDV-specific CD8+ T-cell immunity. Reinvigorated HDV-specific CD8+ T cells may in turn foster a sustained virological response to BLV treatment, even off therapy. We thus aimed to study the effect of BLV therapy on the HDV-specific CD8+ T-cell repertoire, phenotype and functionality in a unique cohort of 28 patients with chronic hepatitis delta starting BLV monotherapy.

## Patients and methods

A total of 28 patients with cirrhosis caused by chronic hepatitis delta (CHD) starting BLV monotherapy were included in this study. Patients were recruited at the Foundation IRCCS Ca’ Granda Ospedale Maggiore Policlinico, Milan, Italy, the Freiburg University Medical Center, Freiburg, Germany, and the University of Paris-Cité, Hôpital Beaujon, Paris, France. All patients were infected with HDV genotype 1. Patient characteristics are summarized in Table S1. Clinical assessment was performed every 4 weeks; sampling for peripheral blood mononuclear cell isolation was performed every 4–8 weeks during the first 24 weeks and then every 8–16 weeks. The last peripheral blood mononuclear cell sampling and analysis occurred after 40–52 (n = 19) or 80–120 (n = 9) weeks of therapy. HLA class I typing was performed by next-generation sequencing. Written informed consent was obtained from all participants before inclusion. The study was conducted according to federal guidelines and local ethics committee regulations (Albert-Ludwigs-Universität, Freiburg, Germany; #23-1458-S1) and the Declaration of Helsinki (1975).

Methods for immunological analyses, viral sequence analysis, and statistics are supplied in the Supplementary materials.

## Results

### Virological and biochemical response to BLV treatment in a cohort of patients with cirrhosis with CHD

**Fig. 1:**
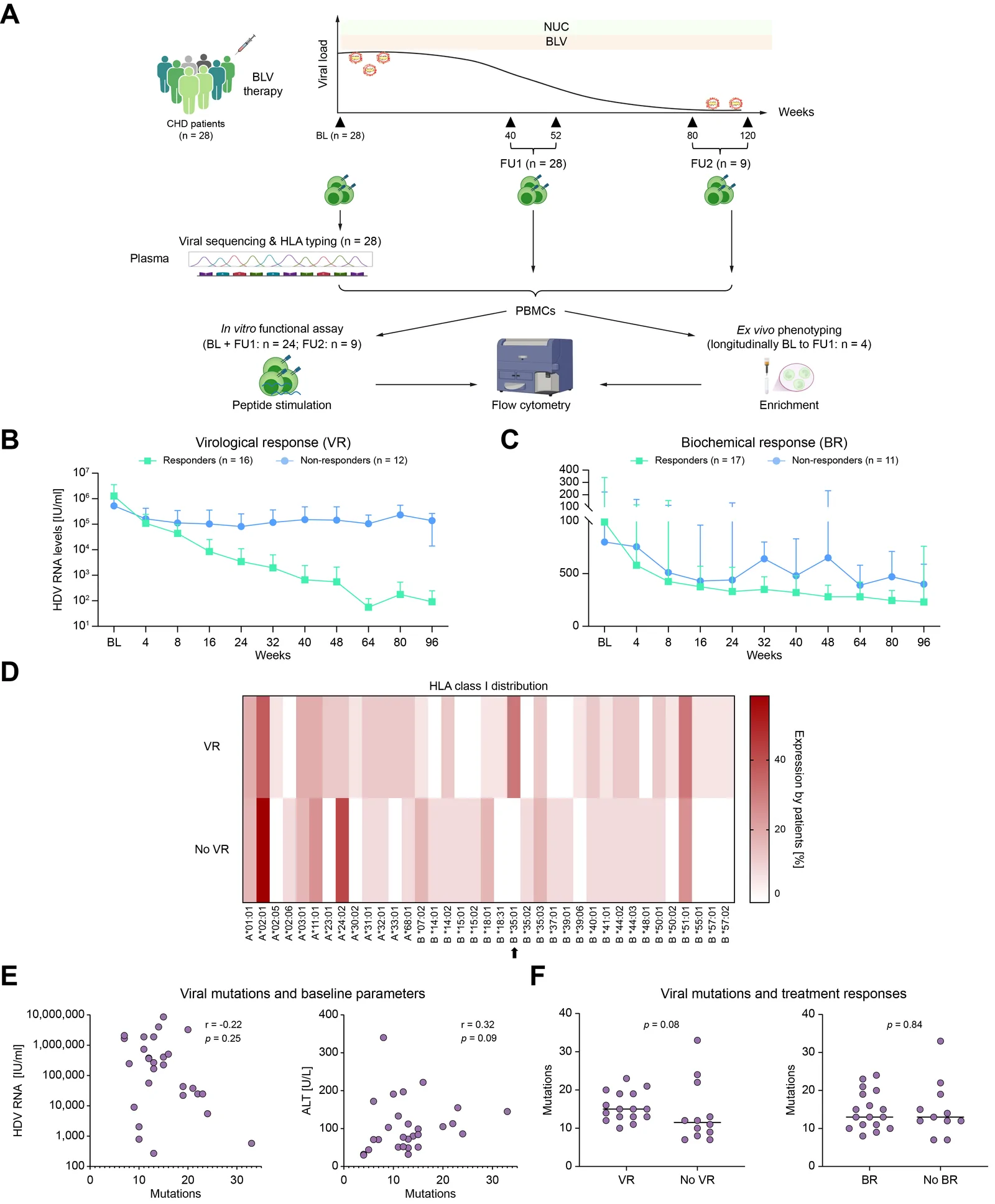
Study design, treatment response, HLA class I, and viral sequence data in a cohort of patients with cirrhosis with chronic hepatitis delta and BLV therapy. (A) Study design. (B) Longitudinal HDV RNA levels in patients with virological response or non-response. (C) Longitudinal ALT levels in patients with biochemical response or non-response. (D) HLA class I distribution in patients with virological response *vs.* virological non-response. (E) Correlation of the number of HDV amino acid variations compared with consensus with HDV RNA levels and ALT levels in the respective patient at baseline. (F) Correlation of the number of HDV amino acid variations compared with consensus with virological and biochemical treatment response. Statistics: (E) Non-parametric Spearman correlation; (F) Mann–Whitney *U* test. ALT, alanine aminotransferase; BLV, bulevirtide; FU1, follow-up 1; FU2, follow-up 2; PBMCs, peripheral blood mononuclear cells.

We recruited a cohort of 28 patients with chronic hepatitis delta starting on BLV monotherapy. All patients had compensated cirrhosis, were infected with HDV genotype 1, and had an HBV backbone treatment with entecavir or tenofovir (Table S1). Patients were followed for 40–120 weeks on treatment (Fig. 1A). At baseline, HDV sequence analysis and HLA class I typing was performed. At baseline (n = 24), Week 40–52 (follow-up 1 [FU1], n = 24), and Week 80–120 (follow-up 2 [FU2], n = 9), comprehensive immunological analyses were performed. For analyses of the immunological results, patients were stratified as virological responders (HDV RNA decline >2 log10 or HDV RNA negative at Week 48) or virological non-responders and biochemical responders (ALT normalization at Week 48) or biochemical non-responders (Fig. 1B,C). Of note, virological and biochemical response rates were in the same range as previously reported (57% and 61%, respectively); they were not linked to baseline HBsAg levels (Fig. S1A). HLA class I typing reflected a typical Caucasian population with balanced HLA class I expression between patients with virological response and non-response, with a single exception for HLA-B∗35:01 which tended to be over-represented in virological responders (*p* = 0.055) (Fig. 1D; HLA-B∗35:01 is indicated by an arrow). HDV sequence analysis revealed an expected HDV variability (median 13, range 7–33 of the 214 amino acids of L-HDAg variant from consensus), with no correlation with HDV RNA or ALT levels at baseline (Fig. 1E) and no association with virological or biochemical treatment response (Fig. 1F).

### HDV-specific CD8+ T-cell responses at baseline and on BLV treatment

**Fig. 2:**
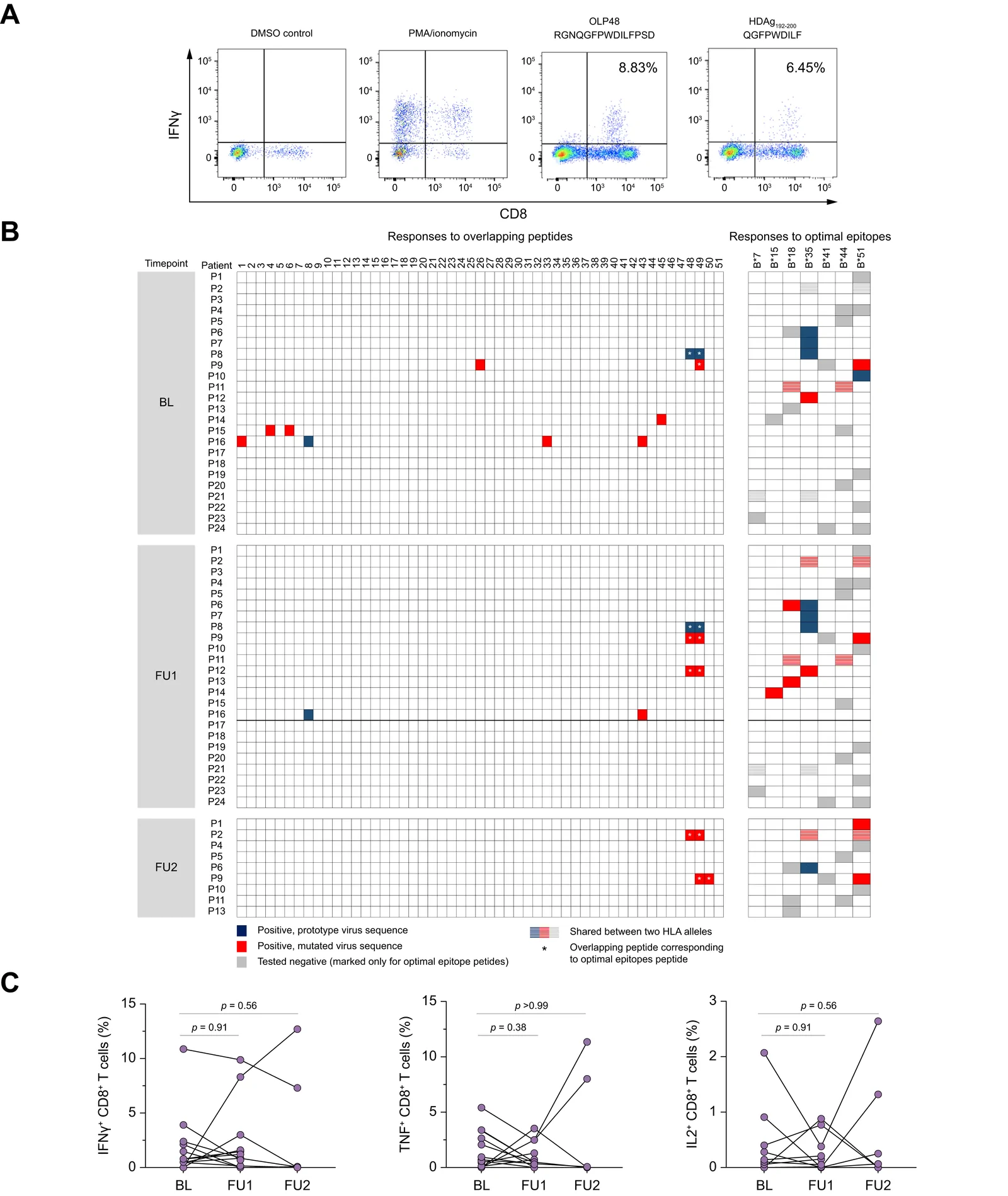
Comprehensive analysis of the longitudinal HDAg-specific CD8+ T-cell response. (A) Exemplary CD8+ T-cell response to an overlapping HDAg peptide and the corresponding pre-described optimal epitope. After peptide-specific culture, intracellular cytokine staining (IFNγ, TNF, and IL-2) was performed after restimulation without an addition of peptide (DMSO negative control), after restimulation with PMA and ionomycin (positive control), or after restimulation with OLP 48 (amino acid indicated) or the pre-described optimal HLA-B∗51-restricted epitope HDAg_192–200_ (amino acid sequence indicated) contained within OLP 48. IFNγ staining is displayed. (B) Positive responses for OLPs and optimal epitope peptides at baseline, FU1 and FU2. Positive responses are displayed in blue (epitopes with conserved amino acid sequence in the respective patient) or red (epitopes with mutated amino acid sequence in the respective patient). For the optimal epitope peptides, only peptides corresponding to the HLA class I alleles of the respective patient were tested; peptides that tested negative are displayed in grey. (C) Longitudinal strength of HDAg-specific CD8+ T-cell responses producing IFNγ, TNF, and IL-2 are displayed. Statistics: (C) Wilcoxon matched-pairs signed rank test. FU1, follow-up 1; FU2, follow-up 2; IFNγ, interferon-gamma; OLP, overlapping peptide; TNF, tumor necrosis factor.

**Fig. 3:**
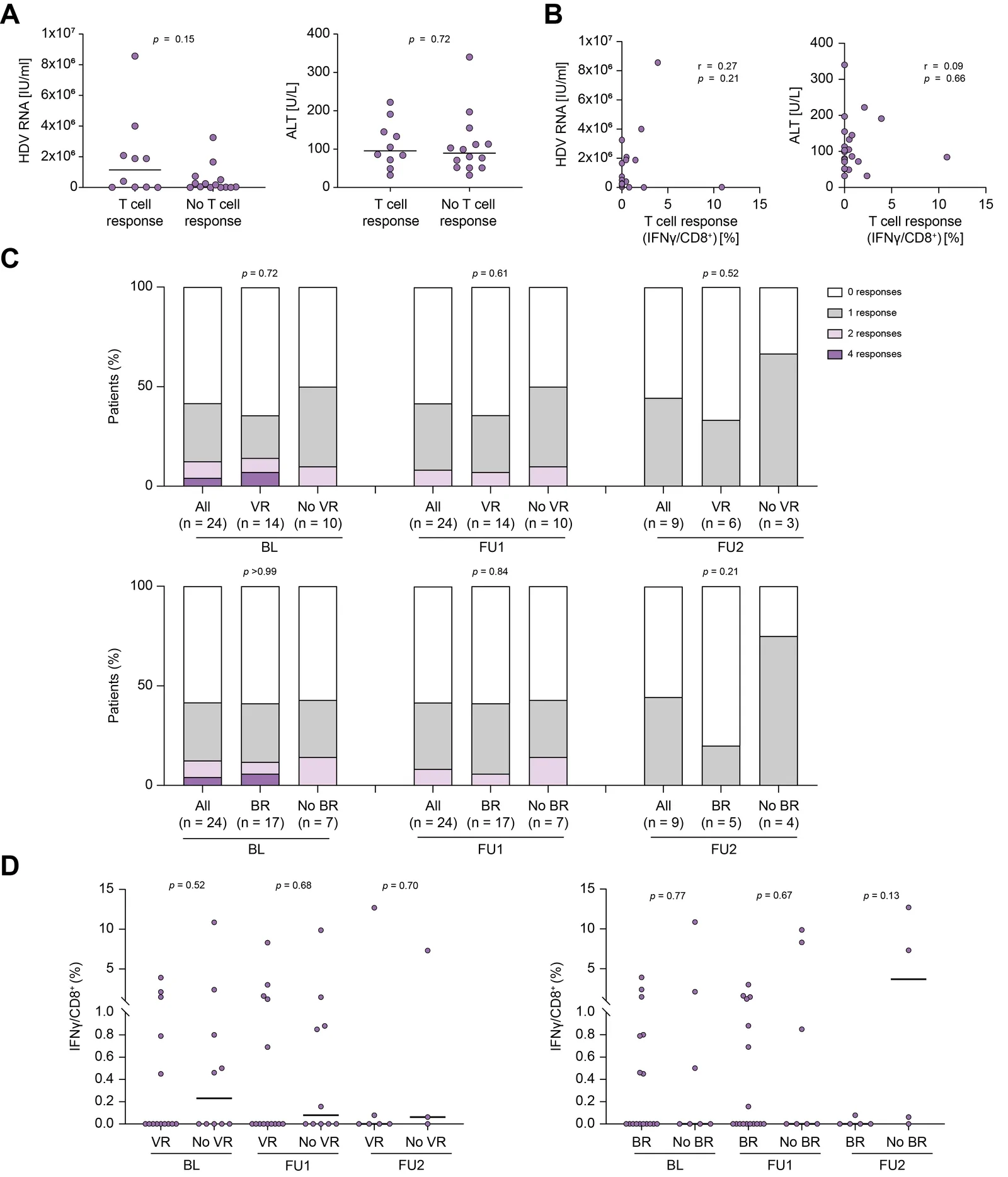
Correlation of the HDAg-specific CD8+ T-cell response with baseline virological and biochemical data and treatment response. (A) Presence or absence of HDAg-specific CD8+ T-cell responses was correlated with HDV RNA and ALT levels. (B) Strength of HDAg-specific CD8+ T-cell responses was correlated with HDV RNA and ALT levels. (C,D) The longitudinal number (C) and strength (D) of HDAg-specific CD8+ T-cell responses is displayed for patients with or without virological response (VR) and with or without biochemical response (BR), respectively. For all graphs, CD8+ T-cell responses detected with OLPs or optimal epitope peptides were combined, counting responses against an OLP and an optimal epitope covered by this OLP only once. Statistics: (A,C,D): Mann–Whitney test; (B): non-parametric Spearman correlation. ALT, alanine aminotransferase; FU1, follow-up 1; FU2, follow-up 2; IFNγ, interferon-gamma; OLPs, overlapping peptides.

We next studied HDV-specific CD8+ T-cell responses longitudinally at baseline and the two follow-up time points during BLV treatment (FU1 ∼1 year on treatment [Week 40–52]; FU2 ∼2 years on treatment [Week 80–120]). In a comprehensive analysis, we used (1) 51 overlapping peptides (OLPs; 14–15mers, overlapping by 11 aa) spanning the complete L-HDAg protein (Table S2); and (2) a set of five pre-described and fine-mapped L-HDAg-specific CD8+ T-cell epitopes restricted by seven different HLA class I alleles (Table S3)[7], [8], [9] and performed peptide-specific expansion for 14 days followed by intracellular interferon-gamma (IFNγ) staining as a sensitive approach (Fig. 2A). Using OLPs, we detected HDV-specific CD8+ T-cell responses in a minority of patients at baseline (five of 24 patients, 20.8%) (Fig. 2B, upper left panel). In patients with positive responses, one to four (median two) responses were detectable per patient. Strikingly, after 1 and 2 years on treatment, the CD8+ T-cell response was not enhanced as hypothesized, but rather contracted. At FU1, CD8+ T-cell responses were detectable in four of 24 patients (16.7%), with one response each in three of these patients and two responses in the fourth patient (Fig. 2B, middle left panel). At FU2, two of nine (22.2%) tested patients displayed a CD8+ T-cell response with one response each (Fig. 2B, lower left panel). Using HLA class I-matched previously described CD8+ T-cell epitope peptides, we observed CD8+ T-cell responses in seven of 24 (29.1%) patients at baseline (Fig. 2B, upper right panel), nine of 24 (37.5%) patients at FU1 (Fig. 2B, middle right panel), and four of nine (44.4%) patients at FU2 (Fig. 2B, lower right panel). All patients were positive for a single pre-described CD8+ T-cell epitope, except for one patient who targeted two pre-described CD8+ T-cell epitopes at FU2. As expected, CD8+ T-cell responses identified by OLPs and pre-described CD8+ T-cell epitope peptides partially overlapped (marked by an asterisk in Fig. 2B). Combining results or overlapping peptides and optimal epitope peptides, 10/24 (41.7%), 10/24 (41.7%), and four of nine (44.4%) patients displayed a positive response at baseline, FU1, and FU2, respectively. When we longitudinally compared the strength of the positive CD8+ T-cell responses regarding production of IFNγ, tumor necrosis factor (TNF), and IL-2, we did not observe a significant evolution between baseline, FU1 and FU2 time points (Fig. 2C; a separate analysis of responses towards OLPs and pre-described epitopes is displayed in Fig. S2). In a subset of patients, we studied HDV-specific CD8+ T cells every 4–8 weeks of the first 48 weeks of therapy (n = 11), and at Week 2 of therapy (n = 4). Of note, we did not observe a transient increase of HDV-specific CD8+ T-cell responses in these patients. When we correlated the baseline presence as well as strength of combined positive responses (responses against OLPs + responses against pre-described epitopes) with the baseline HDV RNA levels, ALT levels, as well as HBsAg levels, we did not observe any significant correlation (Fig. 3A,B and Fig. S3A and B). In addition, the number or strength of baseline response was not associated with virological or biochemical response to BLV treatment (Fig. 3C,D, baseline data points; a separate analysis of responses towards OLPs and pre-described epitopes is displayed in Fig. S3C and D). Furthermore, longitudinal evolution of HDV-specific CD8+ T-cell responses did not differ between patients with or without virological or biochemical response (Fig. 3C,D and Fig. S3C and D). The six patients who had a biochemical response without a virological response displayed similar results (three with one CD8+ T-cell response at baseline and FU1, three without a response at both time points), indicating that this dissociation in treatment response is also not related to the HDV-specific CD8+ T-cell response.

### Viral sequence mutations affect the large majority of HDV-specific CD8+ T-cell responses

We and others have described mutational viral escape from CD8+ T-cell responses in chronic HDV infection on both, the individual as well as population level.^7^^,^^8^^,^^10^ We thus wondered how many of the detected CD8+ T-cell responses targeted mutated CD8+ T-cell epitopes. Indeed, the large majority of CD8+ T-cell responses detected in our patient cohort targeted epitopes with a non-prototype sequence: 10/15 (66.7%) responses at baseline, eight of 12 (66.7%) responses at FU1, and three of four (75%) responses at FU2 (Fig. 2B, responses to epitopes with non-prototype sequence displayed in red; Table S4). Of note, for some but not all viral variants viral escape has been demonstrated by functional assays previously.^7^^,^^8^ These data thus suggest that the majority of CD8+ T-cell responses were affected by viral escape. Strikingly, the only CD8+ T-cell response that targeted the prototype sequence in the majority of patients (three of four patients, 75%) and that was maintained throughout treatment was directed against the HLA-B∗35-restricted CD8+ T-cell epitope L-HDAg_192–200/194–202_ (Fig. 2B).

### Partial phenotypic amelioration of HLA-B∗35 restricted HDV-specific CD8+ T cells targeting the wild-type epitope

**Fig. 4:**
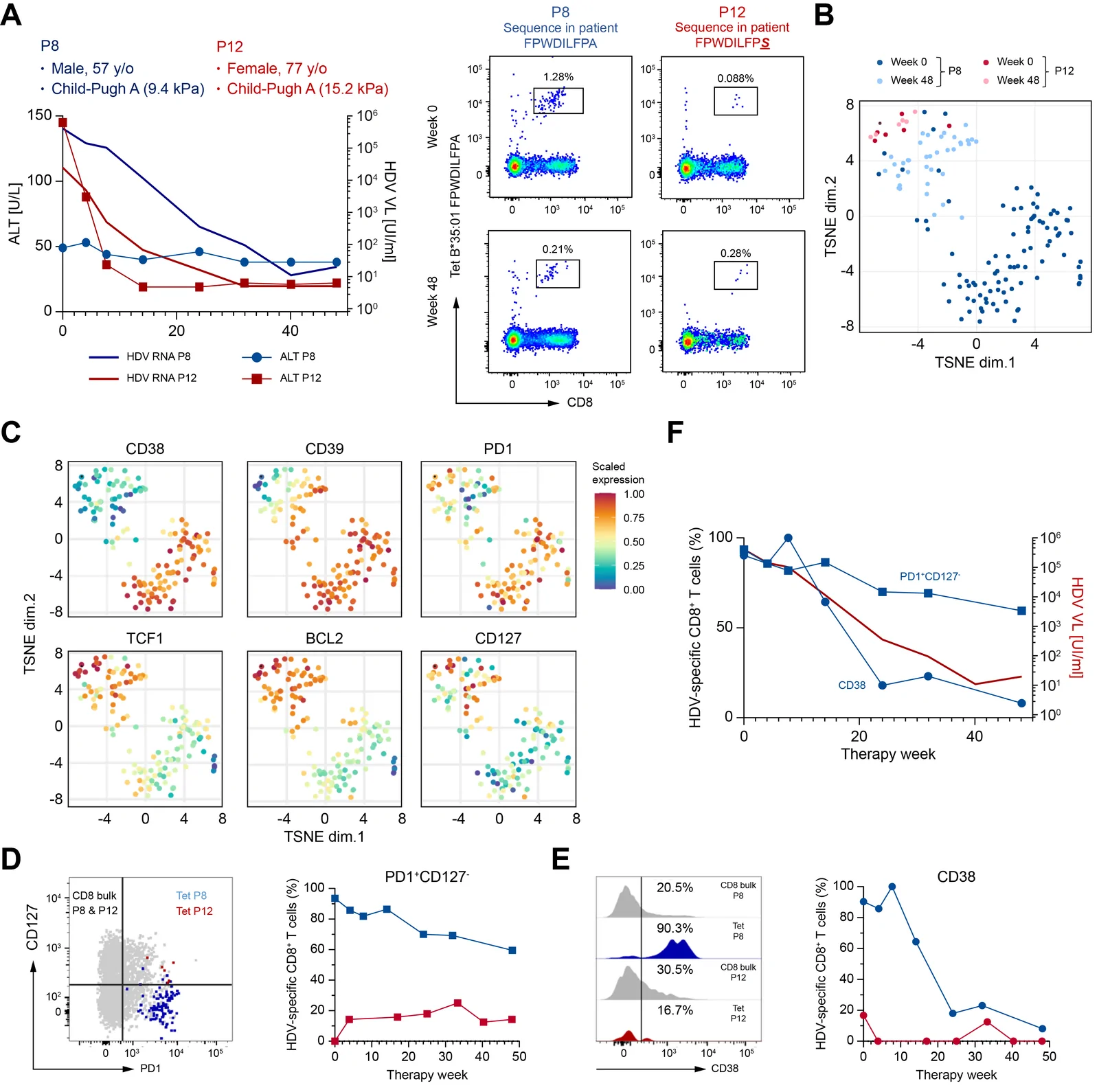
BLV therapy ameliorates HDV-specific CD8+ T cells targeting epitopes with prototype viral sequence only. (A) Clinical data and tetramer staining of HLA-B∗35:01-restricted HDAg_192–200_-specific CD8+ T cells in patient P8 (prototype viral sequence) and patient P12 (mutated epitope sequence) at baseline and treatment Week 48, respectively. (B) t-SNE analysis of HDAg_192–200_-specific CD8+ T cells from the two patients (P8 and P12) and the two time points (baseline and Week 48). (C) Expression level of the markers CD38, CD39, PD1, TCF1, BCL2, and CD127 blotted and the t-SNE distribution displayed in (B). (D) Longitudinal percentage of PD1+CD127− HDAg_192–200_-specific CD8+ T cells in patients P8 and P12. The blot displays the expression of CD127 and PD1 at baseline. (E) Longitudinal percentage of CD38+ HDAg_192–200_-specific CD8+ T cells in patients P8 and P12. The blot displays the expression of CD38 at baseline. (F) Longitudinal correlation of HDV RNA level, CD38 expression on HDAg_192–200_-specific CD8+ T cells, and percentage of PD1+CD127− HDAg_192–200_-specific CD8+ T cells in patient P8. ALT, alanine aminotransferase; t-SNE, T-distributed Stochastic Neighbor Embedding.

In chronic HCV infection, sustained virological response to direct-acting antiviral therapy results in partial phenotypical restoration of HCV-specific CD8+ T cells with a shift from predominantly terminally exhausted to memory-like CD8+ T cells.[11], [12], [13] This effect is limited to CD8+ T cells targeting wild-type epitopes, because CD8+ T cells targeting escaped epitopes display a memory-like phenotype already before antiviral therapy, because of a lack of antigen triggering by the mutated epitope. We hypothesized that in patients with virological response to BLV therapy a similar amelioration of virus-specific CD8+ T cells may occur. We focused on the HLA-B∗35 restricted CD8+ T-cell epitope L-HDAg_194–202_, because this was the only fine-mapped HDV-specific CD8+ T-cell epitope with predominance of the wild-type epitope that was longitudinally detectable during BLV treatment in our patient cohort. Importantly, we were able to detect L-HDAg_194–202_-specific CD8+ T cells *ex vivo* in two HLA-B∗35:01+ patients: Patient P8 with a wild-type epitope sequence, and patient P12 with a variant epitope sequence. We performed a multiparameter peptide/HLA class I tetramer staining for a comprehensive panel of activation, exhaustion, and memory markers longitudinally over 48 weeks of BLV treatment (Fig. 4A, exemplary plots for tetramer-positive cells as well as baseline characteristics and on-treatment kinetics of ALT and HDV RNA levels of the two patients). T-distributed Stochastic Neighbor Embedding (t-SNE) analysis revealed that the HDV-specific CD8+ T cells from patient P8 targeting the wild-type epitope underwent phenotypical evolution from baseline to week 48 of BLV treatment (Fig. 4B, data of patient P8 displayed in blue color). Of note, the phenotypical evolution continuously progressed throughout the 48 weeks of BLV treatment (Fig. S4A depicts the t-SNE analysis for all time points analyzed). On a single marker level, HDV-specific CD8+ T cells expressed high levels of the activation markers CD38 and CD39 as well as the exhaustion-associated marker PD-1 at baseline, but a reduced expression of these markers and an increasing level of the memory and survival markers CD127, BCL2, and TCF1 during BLV treatment (Fig. 4C and Fig. S4B). Indeed, HDV-specific CD8+ T cells shifted from a terminally exhausted (PD1+CD127−) to a memory-like phenotype (PD1+CD127+) during BLV treatment (Fig. 4D). Of note, HDV-specific CD8+ T cells in patient P12 targeting a variant epitope sequence did not phenotypically evolve during BLV therapy, but displayed a memory-like phenotype already at baseline, in line with the assumption that the CD8+ T cells did not experience antigen triggering by the variant epitope (Fig. 4B, data of patient P12 displayed in red color). For the activation marker CD38, differences between HDV-specific CD8+ T cells targeting a conserved epitope (patient P8) *vs*. a mutated epitope (patient P12) became most evident (Fig. 4E). For CD39, TCF1 and BCL2, differences between T cells targeting the conserved *vs*. mutated epitope were also obvious, but the temporal evolution was less marked compared with PD1; additional markers such as KLRG1, EOMAS, Tbet, TIGIT, and TOX did not substantially contribute to differential phenotypes or temporal evolution (Fig. S4C). Of note, the expression of activation, memory, and survival markers did not evolve differentially on memory-like (PD1+CD127+; Fig. S4D) *vs*. terminally exhausted (PD1+CD127−; Fig. S4E) HDV-specific CD8+ T cells. Importantly, the global CD8+ T-cell population in the two patients did not show a phenotypic evolution during BLV therapy, further supporting that the phenotypic changes are indeed limited to HDV-specific CD8+ T cells that are triggered by antigen (Fig. S4F). When we compared the temporal decline of HDV RNA and the decline of the activation maker CD38 on HDV-specific CD8+ T cells as well as the frequency of terminally exhausted PD1+CD127− HDV-specific CD8+ T cells in patient P8 (conserved epitope), we observed that CD38 expression declined synchronically with HDV RNA levels, while the decline of terminally exhausted HDV-specific CD8+ T cells was delayed (Fig. 4F). This observation suggests that the decline of HDV viremia reduces activation of HDV-specific CD8+ T cells, leading to a delayed shift from predominantly terminally exhausted to maintained memory-like HDV-specific CD8+ T cells. It thus argues against a causal role of HDV-specific CD8+ T cells in HDV control during BLV treatment.

**Fig. 5:**
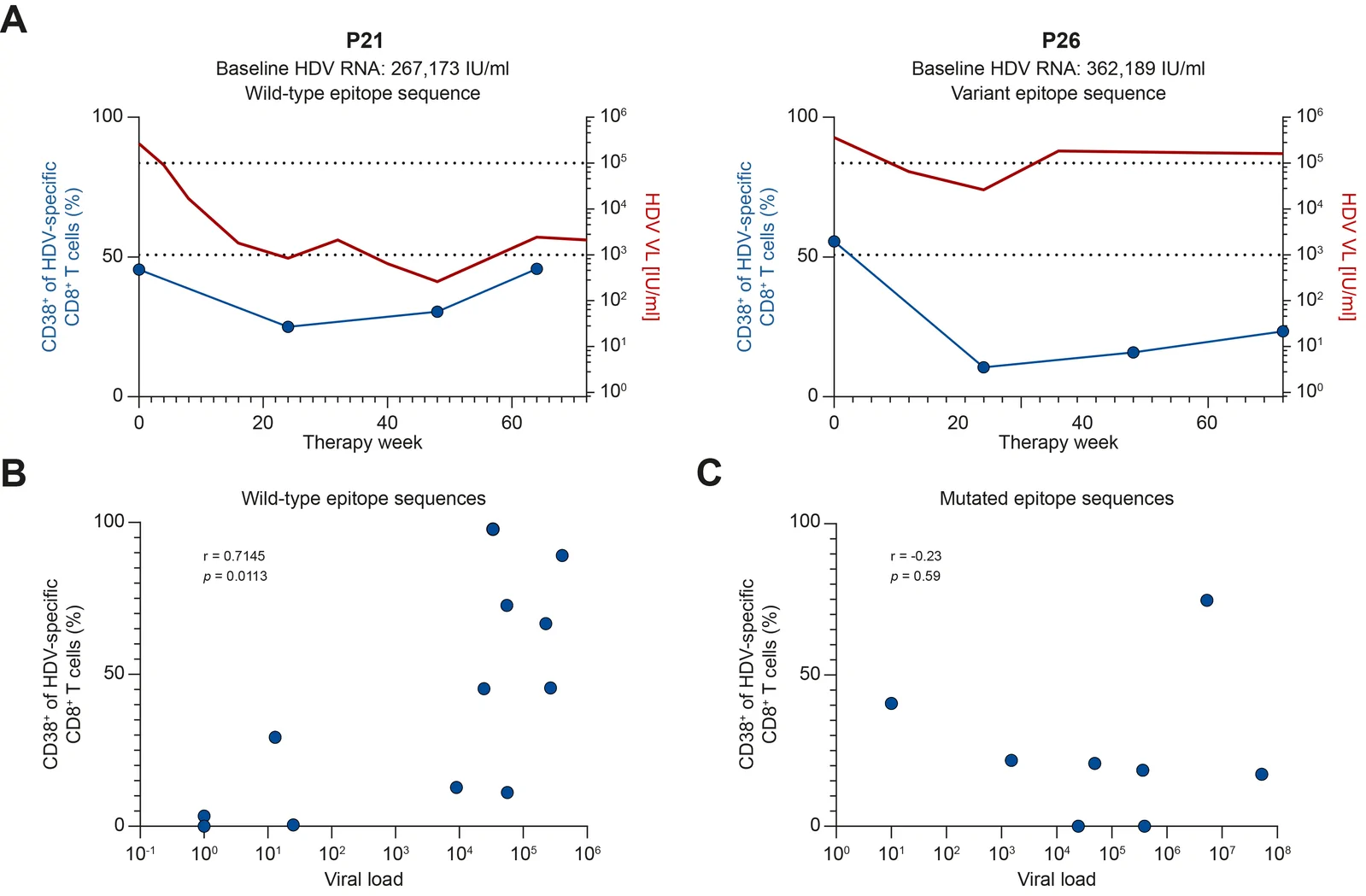
CD38 expression by HDAg-specific CD8+ T cells mirrors the HDV RNA course in patients treated with BLV with viral rebound and correlates with HDV RNA in untreated patients. (A) Longitudinal HDV RNA levels and CD38 expression by HDAg-specific CD8+ T cells in BLV-treated patients P21 and P26. (B) Correlation of HDV RNA levels and percentage of CD38+ HDAg-specific CD8+ T cells in 11 BLV-naïve patients with chronic HDV infection displaying consensus epitope sequence (baseline data from patients P8, P21, P27, and P28 from the cohort of patients treated with BLV and patients P29–P34, P39, and P40 without BLV treatment; compare Tables S1 and S4). (C) Correlation of HDV RNA levels and percentage of CD38+ HDAg-specific CD8+ T cells in 8 BLV-naïve patients with chronic HDV infection displaying variant epitope sequence (Tables S1 and S4: baseline data from patients P12, P25, and P26 from the cohort of patients treated with BLV; patients P31 and P35–P38 without BLV treatment). Statistics: (B,C): non-parametric Spearman correlation. BLV, bulevirtide.

We extended the HDV-specific CD8+ T cell phenotype analyses in patients P21 and P26 who displayed an initial HDV RNA decline, followed by a partial viral rebound on treatment (Fig. 5A). HDV-specific CD8 T cells of these patients displayed similar kinetics of CD38 expression, with an initial decline followed by a rebound. Effects were more obvious for patient P21 targeting a wild-type epitope and more moderate in patient P26 targeting an epitope with viral variant sequence treatment (Fig. 5A). These data further support the concept that the partial amelioration is mainly driven by evolving HDV RNA load and not by the BLV treatment *per se*. This concept is further supported by a clear correlation between the activation marker CD38 on HDV-specific CD8+ T cells targeting conserved HDV epitopes and HDV RNA levels in BLV-naïve patients (Fig. 5B; these data include baseline data from patients P8, P27, and P28 from the cohort of BLV-treated patients and patients P29–P34, P39, and P40 without BLV treatment; for patient and epitope data compare Table S1 and Table S4, respectively). Of note, the same association was not found for HDV-specific CD8+ T cells targeting variant HDV epitopes in BLV-naïve patients (Fig. 5C; baseline data from patients P12, P25, and P26 from the cohort of BLV-treated patients and patients P31 and P35–P38 without BLV treatment; for patient and epitope data compare Tables S1 and S4, respectively).

## Discussion

To our knowledge, this is the first study investigating T-cell responses in patients with CHD patients with cirrhosis treated with BLV monotherapy. In this analysis conducted on 28 patients, we found baseline detectable HDV-specific CD8+ T-cell response in 42%, but no substantial increase during BLV treatment for 40–120 weeks. Moreover, the large majority of HDV-specific CD8+ T-cell responses targeted viral epitopes with sequence variations consistent with viral escape mutations. This observation suggests that antiviral immunity is limited in its ability to support sustained virological response, even in the long-term. Of note, cure of HCV infection with direct-acting antivirals (DAA) leads to amelioration of HCV-specific CD8+ T cells, with a decline of terminally exhausted HCV-specific CD8+ T cells and an increase of memory-like HCV-specific CD8+ T cells, associated with an increase of pro-survival transcription factors and enhanced functional capacities.^11^^,^^12^ These partially restored HCV-specific CD8+ T cells are thought to contribute to sustained virological response after DAA treatment of chronic HCV infection.

In contrast to HCV-specific CD8+ T cells, little is known about the fate of HDV-specific CD8+ T cells during antiviral therapy of CHD with either pegIFNα or BLV. Indeed, virological response to pegIFNα treatment was shown to be associated with sustained production of cytokines most likely by HDV-specific CD8+ T cells, whereas patients failing treatment displayed decreasing cytokine production during pegIFNα treatment.^14^ Further analyses, including phenotypical analyses of HDV-specific CD8+ T cells was, however, not performed in this study. For BLV treatment, a very recent study demonstrated a shift in NK cell phenotype in patients with biochemical response *vs*. those without biochemical response.^15^ However, HDV-specific CD8+ T cells were so far not studied during BLV treatment. We thus hypothesized that a partial restoration of HDV-specific CD8+ T cells during BLV treatment may contribute to viral suppression during treatment and prevention of HDV relapse off treatment.

Surprisingly, when we analyzed our cohort of patients with chronic HDV infection and progressed liver disease undergoing BLV treatment, we observed rather a decrease then an increase of HDV-specific CD8+ T cells. We used a sensitive method, including peptide-specific expansion of HDV-specific CD8+ T cells followed by intracellular cytokine staining (IFNγ, TNF, and IL-2) and used both, overlapping peptides spanning the complete L-HDAg as well as a set of five previously described fine-mapped HDV-specific CD8+ T-cell epitope peptides restricted by seven HLA-class I alleles. While this experimental set-up is very sensitive to detect antiviral CD8+ T cells capable to produce antiviral cytokines, it cannot detect terminally exhausted virus-specific CD8+ T cells that are severely compromised in their capability to expand and to produce antiviral cytokines. Thus, the fact that we did not observe an increased frequency of HDV-specific CD8+ T cells during BLV treatment even stronger argues against a general increase or functional restoration of HDV-specific CD8+ T cells during BLV treatment in our cohort.

When we correlated our data on targeted HDV-specific CD8+ T-cell epitopes with the autologous viral sequences obtained from the respective patients, we found that at baseline, two-thirds of HDV-specific CD8+ T-cell responses targeted epitope sequences with amino acid variations compared with the prototype sequence, suggesting viral escape.^7^ Strikingly, during BLV treatment, the HDV-specific CD8+ T-cell response seemed to narrow and focus on an HLA-B∗35-restricted CD8+ T-cell epitope that displayed a conserved viral sequence in the majority of patients. Intriguingly, HLA-B∗35:01 was the only HLA class I allele that was enriched in patients with virological response in our cohort. It is important to note, however, that this association was only borderline significant (*p* = 0.055) and was only present for HLA-B∗35:01 but not B∗35:02 or B∗35:03. Thus, definitively larger patient cohorts will be needed to confirm a beneficial role of HLA-B∗35:01 during BLV treatment.

The positive detection of HLA-B∗35-restricted HDV-specific CD8+ T cells by peptide/HLA class I tetramer staining allowed us to perform a multiparameter phenotypical analysis of HDV-specific CD8+ T cells during BLV treatment. Of note, HDV-specific CD8+ T cells targeting the prototype sequence displayed a terminally exhausted phenotype at baseline, but shifted to a memory-like phenotype gradually during the first 48 weeks of BLV treatment. By contrast, HDV-specific CD8+ T cells targeting a variant epitope sequence in another HLA-B∗35+ patient displayed a memory-like phenotype already at baseline that did not change during BLV treatment, consistent with the notion that these HDV-specific CD8+ T cell did not recognize their cognate antigen and are thus not driven into exhaustion by repetitive antigen triggering. When we correlated the evolution of the HDV-specific CD8+ T-cell phenotype and the HDV RNA level during the course of BLV treatment, we observed that the phenotypic changes followed the decrease of viral load, suggesting that the amelioration of the CD8+ T-cell phenotype is rather a consequence and not a cause of viral control and antigen reduction. The observed shift toward a memory-like phenotype thus reflects reduced antigenic stimulation rather than immune recovery, explaining that it does not translate into functional improvement or contribution to viral control. This concept was also supported by the correlation of the expression of the activation marker CD38 on HDV-specific CD8+ T cells and HDV RNA levels in patients not undergoing antiviral treatment, and the similar evolution of expression of other markers by memory-like (PD1+CD127+) *vs*. terminally exhausted (PD1+CD127−) subsets. Interestingly, our findings in HDV infection partially mirror previous findings in HCV infection.^11^^,^^12^ In both infections, memory-like virus-specific CD8+ T cells are enriched compared with terminally exhausted T cells during antiviral therapy, resulting in an overall increased expression of survival markers such as TCF-1 and BCL2 on virus-specific CD8+ T cells. These changes were, however, much more pronounced in HCV infection, likely linked to sustained viral control in this infection, and rather decent in HDV, where HDV is rather controlled then completely eliminated. On the memory-like T cell subset, however, only the activation marker CD38 showed a substantial decline during antiviral treatment in both infections, indicating that rather a shift of T-cell subsets then a real reprogramming of virus-specific CD8+ T cells occurred.

Our data thus indicate that amelioration of HDV-specific CD8+ T cells during BLV treatment is possible in principle, however, most HDV-specific CD8+ T-cell epitopes are affected by viral escape. Thus, in the overall cohort, amelioration of HDV-specific CD8+ T cells does not play a substantial role. These results partially explain that long-term BLV treatment is necessary to prevent viral relapse.

Of note, our study has some limitations. First, decline of HDV viremia is slow during BLV monotherapy, so amelioration of HDV-specific CD8+ T cells may require more time than 1–2 years. Second, all patients analyzed in our study had advanced liver disease, possibly limiting antiviral CD8+ T-cell immunity. However, we did not observe a clear difference between HDV-specific CD8+ T-cell responses in patients with cirrhosis *vs*. patients without cirrhosis when we analyzed an independent cohort of 60 patients with hepatitis delta, using the same approach (unpublished data). Third, our cohort only included patients with BLV monotherapy (on a nucleos(t)ide analogue backbone), but did not include antiviral treatment aiming at HBsAg reduction. As HDV contains both HDAg and HBsAg as possible targets of antiviral CD8+ T-cell immunity, it will be important to study HDAg- as well as HBsAg-specific CD8+ T cells in patients undergoing BLV/pegIFN combination therapy as well as in patients undergoing novel treatments such as monoclonal antibodies and siRNA. These treatments may restore HBsAg-specific CD8+ T cells and contribute to prevention of HDV relapse. Fourth, our findings are based on a rather small number of patients with detectable HDV-specific CD8+ T cell responses. Viral escape as a frequent limitation of HDV-specific CD8+ T-cell responses has been supported by studies from us and others.^7^^,^^8^ The finding that BLV treatment allows amelioration of HDV-specific CD8+ T cell responses targeting wild-type epitopes clearly requires confirmation in future studies. Fifth, our study only analyzed peripheral HDV-specific CD8+ T cells, while these are likely enriched in the liver and may also differentially react to antiviral therapy. Sixth, we limited our study to the CD8+ T-cell compartment. In the OLP experiments, we also detected a small number of CD4+ T cell responses without a trend towards enhanced number or magnitude during BLV treatment, but this observation requires confirmation. In addition to these conceptual limitations, our study also has some methodical limitations. Although the combined use of overlapping peptides and optimal peptides of described HDV-specific CD8+ T-cell epitopes is the most comprehensive approach used to date, some HDV-specific CD8+ T-cell responses may have been missed because of the use of peptides based on the HDV genotype 1 consensus sequence and not patient-specific sequences. In addition, overlapping peptides with the length of 14 or 15 amino acids do not always stimulate CD8+ T cells that usually recognize 8- to 10-mer epitope peptides. In line with this limitation, a combined analyses of responses towards pre-described epitope peptides and OLPs did not differ from (and not substantially add to) the analyses performed with pre-described epitope peptides only. Further, because of the very low frequencies of HDV-specific CD8+ T cells, we could not perform functional assays *ex vivo*, thus we can only speculate on the functional implications of the phenotypic changes observed *ex vivo*.

In sum, HDV-specific CD8+ T cells can be ameliorated during BLV monotherapy, but this effect likely has little impact on antiviral control during BLV treatment or on prevention of HDV relapse off treatment, because viral escape is the dominant mechanism underlying the ineffective HDV-specific CD8+ T-cell response.

## Abbreviations

ALT, alanine aminotransferase; BLV, bulevirtide; CHD, chronic hepatitis delta; DAA, direct-acting antivirals; FU1, follow-up 1; FU2, follow-up 2; IFNγ, interferon-gamma; OLPs, overlapping peptides; PBMCs, peripheral blood mononuclear cells; pegIFNα, pegylated interferon-alpha; TNF, tumor necrosis factor; t-SNE, T-distributed Stochastic Neighbor Embedding.

## Authors’ contributions

Conceptualization: PL, CNH. Recruitment of patients: ED, MB, RS, MPA, FF, SN, TA, TB, BB, HL. Four-digit HLA-typing: FE. Designs and synthesis of peptide/HLA class I tetramers: SLL, DAP. Performance and analysis of viral sequencing and immunological data: VO, MM, KH, ESA, ÖS, FW. Interpretation of data: VO, MM, MH, RT, PL, CNH. Drafting of the manuscript: VO, MM, HL, MH, RT, PL, CNH. Reviewing and editing of the manuscript: all authors. Approved the submitted version: all authors.

## Data availability

No data are deposited on public databases. All requests for raw and analyzed data and materials will be reviewed by the corresponding author to verify if the request is subject to any confidentiality obligations. Patient-related data not included in the paper were generated as part of clinical examination and may be subject to patient confidentiality. Any data and materials that can be shared will be released via a material transfer agreement.

## Financial support

This project was supported by grants from the Deutsche Forschungsgemeinschaft (DFG, German Research Foundation; TRR-179, project number 272983813, to TB, BB, HL, MH, RT, and CNH; SFB-1160, project number 256073931, to BB, MH, RT, and CNH) and the Deutsches Zentrum für Infektionsforschung (DZIF, German Center for Infection Research; project number TTU 05.822, to CNH). MH was supported by the DFG Heisenberg program (project no. HO 5836/2-1). DAP was supported by the PolyBio Research Foundation (Balvi B43). The authors thank the D-SOLVE consortium, a European Union funded project (No 101057917).

## Conflicts of interest

ED: on advisory board for Gilead Sciences, IPSEN, and Roche; speaking and teaching for Gilead Sciences and IPSEN; travel grant from Gilead Sciences; and research grant from Gilead Sciences. TA: consultant, expert, and speaker for Bluejay, Gilead Sciences, GSK, Janssen, MYR Pharmaceuticals, and VIR Biotechnology. TB: advisor and speaker bureau for Gilead Sciences and Falk Foundation. BB: advisor for Roche and speaker bureau for Falk Foundation, BMS, AstraZeneca, and Roche. RT: speaker bureau for Bayer Vital and Falk Foundation and advisor for AbbVie, Gilead Sciences, OPASCA, Topas Therapeutics, and Roche. PL: advisor and speaker bureau for Roche Pharma/Diagnostics, Gilead Sciences, GSK, AbbVie, Janssen, MYR, Eiger, Antios, Aligos, VIR, Grifols, Altona, Roboscreen, and Bluejay. CN–H: speaker bureau for AbbVie, Gilead Sciences, MSD, Pfizer, and Falk Foundation. The remaining authors declare no conflicts of interest that pertain to this work.

Please refer to the accompanying ICMJE disclosure forms for further details.

## References

[1] Asselah T., Rizzetto M. (2023). Hepatitis D virus infection. N Engl J Med.

[2] Degasperi E., Anolli M.P., Jachs M. (2025). Real-world effectiveness and safety of bulevirtide monotherapy for up to 96 weeks in patients with HDV-related cirrhosis. J Hepatol.

[3] Wedemeyer H., Aleman S., Brunetto M. (2024). Bulevirtide monotherapy in patients with chronic HDV: efficacy and safety results through week 96 from a phase III randomized trial. J Hepatol.

[4] Wedemeyer H., Aleman S., Brunetto M.R. (2023). A phase 3, randomized trial of bulevirtide in chronic hepatitis D. N Engl J Med.

[5] Wedemeyer H., Aleman S., Blank A. (2026). 144 Weeks of bulevirtide monotherapy for chronic hepatitis D: final and posttreatment results from a Phase 3 randomized trial. J Hepatol.

[6] European Association for the Study of the Liver (2023). EASL clinical practice guidelines on hepatitis delta virus. J Hepatol.

[7] Karimzadeh H., Kiraithe M.M., Oberhardt V. (2019). Mutations in hepatitis D virus allow it to escape detection by CD8+ T cells and evolve at the population level. Gastroenterology.

[8] Kefalakes H., Koh C., Sidney J. (2019). Hepatitis D virus-specific CD8+ T cells have a memory-like phenotype associated with viral immune escape in patients with chronic hepatitis D virus infection. Gastroenterology.

[9] Landahl J., Bockmann J.H., Scheurich C. (2019). Detection of a broad range of low-level major histocompatibility complex class II-restricted, hepatitis delta virus (HDV)-specific T-cell responses regardless of clinical status. J Infect Dis.

[10] Karimzadeh H., Kiraithe M.M., Kosinska A.D. (2018). Amino acid substitutions within HLA-B∗27-restricted T cell epitopes prevent recognition by hepatitis delta virus-specific CD8+ T cells. J Virol.

[11] Hensel N., Gu Z., Sagar (2021). Memory-like HCV-specific CD8+ T cells retain a molecular scar after cure of chronic HCV infection. Nat Immunol.

[12] Tonnerre P., Wolski D., Subudhi S. (2021). Differentiation of exhausted CD8+ T cells after termination of chronic antigen stimulation stops short of achieving functional T cell memory. Nat Immunol.

[13] Wieland D., Kemming J., Schuch A. (2017). TCF1+ hepatitis C virus-specific CD8+ T cells are maintained after cessation of chronic antigen stimulation. Nat Commun.

[14] Grabowski J., Yurdaydin C., Zachou K. (2011). Hepatitis D virus-specific cytokine responses in patients with chronic hepatitis delta before and during interferon alfa-treatment. Liver Int.

[15] Chen P.C., Deterding K., Engelskircher S.A. (2025). TIGIT expression on natural killer cell subsets is an early indicator of alleviating liver inflammation following bulevirtide treatment in chronic hepatitis D. Hepatology.

