# Supplementary material for: Fate of hepatitis D virus-specific CD8+ T cells during bulevirtide monotherapy in patients with chronic hepatitis delta
Source: JHEP Rep. 2026 Jul 24;8(10):101970. doi: 10.1016/j.jhepr.2026.101970 (PMC13594906; doi:10.1016/j.jhepr.2026.101970)
Supplement: Supplementary file 1 [file mmc1.pdf]

Fate of hepatitis D virus-specific CD8+ T cells during bulevirtide monotherapy in patients with chronic hepatitis delta

Valerie Oberhardt, Elisabetta Degasperi, Michelle Maas*, Marta Borghi, Kathrin Heim, Roberta Soffredini, Maria Paola Anolli, Floriana Facchetti, Stéphanie Narguet, Tarik Asselah, Elahe Salimi Alizei, Özlem Sogukpinar, Frances Winkler, Sian Llewellyn-Lacey, Florian Emmerich, David A. Price, Tobias Boettler, Bertram Bengsch, Hendrik Luxenburger, Maike Hofmann, Robert Thimme, Pietro Lampertico, Christoph Neumann-Haefelin

Table of contents

Supplementary methods……………………………………………………………………………2
Table S1……………………………………………………………………………………………...6
Table S2……………………………………………………………………………………………...7
Table S3……………………………………………………………………………………………...9
Table S4…………………………………………………………………………………………….10
Fig. S1……………………………………………………………………………………………...11
Fig. S2……………………………………………………………………………………………12
Fig. S3……………………………………………………………………………………………13
Fig. S4……………………………………………………………………………………………15
Fig. S5……………………………………………………………………………………………20

Supplementary methods

PBMC isolation

Venous blood samples (50 mL per draw) were collected in EDTA-anticoagulated tubes. Peripheral blood mononuclear cells (PBMCs) were isolated with lymphocyte separation medium by density gradient centrifugation (Pancoll separation medium, PAN Biotech GmbH; Aidenbach, Germany). PBMCs were stored at -80°C. Frozen PBMCs were thawed and resuspended in RPMI 1640 medium supplemented with 10% fetal calf serum, 1% penicillin/streptomycin, and 1.5% HEPES buffer 1 mol/L containing 50 U/mL benzonase (Sigma-Aldrich) (RPMI complete medium; all additives from ThermoScientific; Waltham, MA).

Peptides and tetramers for T-cell analysis

A total of 51 overlapping peptides (OLP) spanning the L-HDAg (consensus HDV genotype 1 sequence obtained from 104 patients [1]) were synthesized as 14- or 15-mers overlapping by 11 amino acids with an unmodified amine NH2 terminus and an amidated acid COOH terminus with standard Fmoc chemistry and a purity of >70% (Genaxxon Bioscience, Ulm, Germany; **Table S2**). Additionally, peptides for optimal epitopes (9-10-mers) were synthesized in the same manner (**Table S3**). Peptide/HLA-class I tetrameric complexes were generated as described previously [2]. For HLA-B*08:01 tetramers, peptide was loaded on HLA class I easYmers (immunAware) according to the manufacturer´s instructions. For tetramerization, the HDV peptide-loaded HLA class I monomers were subsequently incubated together with phycoerythrin (PE)- conjugated streptavidin (Agilent) following the manufacturer´s protocol.

In vitro expansion and intracellular IFNγ staining using overlapping peptides or pre-described optimal CD8+ T-cell epitopes

In vitro expansion with OLPs was performed as follows: 20% of the PBMCs were stimulated with a pool of all 51 OLPs (5 µM/peptide) for 1 h at 37 °C, washed and co-cultured with the remaining PBMCs in RPMI complete medium supplemented with 20 U/mL with recombinant interleukine-2 (IL-2) (StemCell Technologies) and anti-CD28 monoclonal antibody (0.5 µg/mL) (BD Bioscience). Alternatively, PBMCs were stimulated with optimal peptides (10 µM) for *in vitro* expansion. Cells were then fed every 3 days with RPMI complete medium containing 20 U/mL recombinant interleukin (IL-2). On day 10, intracellular IFNγ staining was performed with 12 smaller pools of OLPs (9 pools with 4 OLPs each and 3 pools with 5 OLPs

each). Therefore, cells were re-stimulated with OLP pools (25 µM/peptide for 1h; 5 µM/peptide final concentration), dimethyl sulfoxide as a negative control or phorbol 12-myristate 13-acetate and ionomycin as positive control. After 1h at 37°C, Brefeldin A (GolgiPlug, 0.5 µl/mL) and monensin (GolgiStop, 0.5 µl/mL) (both BD Bioscience) were added. After an additional incubation for 4 h, surface (anti-CD8-APC, anti-CD4-BV421; Viaprobe) and intracellular (anti-IFNγ-FITC) staining was performed. Stained cells were fixed in phosphate-buffered saline (PBS) containing 2% paraformaldehyde (Sigma-Aldrich) and were acquired via flow cytometry. Subsequently, on day 12-14 single OLPs of positive pools and HLA-matched optimal CD8+ T-cell epitopes were tested by intracellular cytokine staining. In particular, cells were re-stimulated with individual OLP (25 µM for 1h; 5 µM final concentration) or optimal CD8+ T-cell epitopes (75 µM for 1h; 15 µM final concentration), dimethyl sulfoxide as a negative control or phorbol 12-myristate 13-acetate and ionomycin as positive control. After 1h at 37°C, Brefeldin A (GolgiPlug, 0.5 µl/mL) and monensin (GolgiStop, 0.5 µl/mL) (both BD Bioscience) were added. After an additional incubation for 4 h, surface (anti-CD8-APC, anti-CD4-BV421; Viaprobe) and intracellular (IFNγ-FITC, TNF-PE-Cy7, anti-IL-2-PerCP-Cy5.5) staining was performed. Data were acquired with and BD FACSCanto II flow cytometer (BD Biosciences) and analyzed with FlowJo software, version 10.6.2 (FlowJo, Ashland, OR).

Analysis of HDV sequences and HLA class I-associated viral polymorphism

Total RNA was extracted from serum or plasma samples using the QiAmp Viral RNA Kit (Qiagen, Hilden, Germany) and with SuperScript III First strand synthesis Kit for RT PCR (Invitrogen, MA, USA) and HDV-specific primer 771R (5´-CGGTCCCCTCGGAATGTTG-3´) reverse transcribed into cDNA. By using GoTaq®G2 Flexi DNA Polymerase (Promega, WI, USA), the L-HDAg-encoding region was amplified from cDNA in a 2-step nested polymerase chain reaction (PCR) incoporating the HDV-specific primers 891F (5´-AGGTCGGACCGCGRGGAGGT-3`), 339R (5´-GCTGAAGGGGTCCTCTGGAGGTG-3`), 912F (5´-GAGATGCCATGCCGACCCGAAGAG-3´) and 1674R (5´-AGAAAAGAGTAAGAGYACTGAGG-3´). According thermal profile was used for the first PCR: (i) 94 °C for 3 minutes; (ii) 94 °C for 1 minute, (iii) 94 °C for 30 seconds, 70 °C-1dT 1 °C (70°-58 °C) for 45 seconds, 72 °C for 90 seconds (12 cycles); (iiii) 94 °C for 30 seconds, 58 °C for 45 seconds, 72 °C for 90 seconds (23 cycles) and (iiiii) 72 °C for 7 minutes. The second PCR thermal profile contains the following steps: (i) 94 °C for 3 minutes; (ii) 97 °C for 1minute, (iii) 94 °C for 30 seconds, 70 °C-1dT 1 °C (70°-55 °C) 45 seconds, 72 °C for 90 seconds (15 cycles); (iiii) 94 °C for 30 seconds, 55 °C for 45 seconds, 72 °C for 90 seconds (20 cycles) and (iiiii) 72 °C for 7 minutes.

PCR products were purified with the QuickStep^{TM}2 PCR purification kit (EdgeBio, CA, USA) and sequenced on an ABI 3730xl DNA Analyzer (Eurofins Genomics Germany GmbH; Ebersberg, Germany) with the internal primers 912F and 1674R. Sequence homology analyses were performed in Genious Prime v.11.0.5. The reference genome of human HDV (M21012.1) was downloaded from the National Center for Biotechnology Information data base (https://www.ncbi.nlm.nih.gov/nuccore/M21012.1). HDV epitopes were then mapped to the corresponding protein alignment.

Magnetic bead-based enrichment of HDV-specific CD8+ T cells

Tetramer-based enrichment was performed as described by Alanio *et al.* [3]. Briefly, 1 x 10^7 – 2 x 10^7 PBMCs were labelled with PE-coupled peptide-loaded HLA class I tetramers for 30 min. Next, the enrichment was performed using anti-PE beads with MACS technology (Miltenyi Biotec) according to the manufacturer´s instructions. Subsequently, analysis of the enriched HDV-specific CD8+ T cells was conducted by multiparametric flow cytometry and frequencies of HDV-specific CD8+ T cells were calculated as follows: Absolute number of HDV-specific CD8+ T cells (enriched sample) divided by the absolute number of CD8+ T cells (pre-enriched sample) x 200. Only samples including > 5 HDV-specific CD8+ T cells were used for further subset gating and phenotypic analyses (detection limit of 5 x 10^{-6}).

Multiparametric flow cytometry for T-cell analysis

The following antibodies were used for multiparametric flow cytometry: anti-CD8-BUV395 (RPA-T8, 1:400), anti-CD45RA-BUV496 (HI100, 1:800), anti-CD127-BUV737 (HIL-7R-M21, 1:50), anti-BCL-2-BV421 (100; 1:100), anti-TIGIT-BV510 (A15153G; 1:8.3), anti-PD-1-BV605 (EH12.1; 1:25), anti-CD39-BV650 (TU66, 1:1.5), anti-KLRG1-BV711 (13F12F2, 1:50), anti-CCR7-BV786 (G043H7, 1:50), anti-TCF1-AlexaFluor488 (C63D9, 1:100) (Cell Signaling), anti-Eomes-PE-Cy5.5 (104; 1:800), anti-CD244-PE-CF594 (C1.7, 1:200), anti-Tbet-PE-Cy7 (4B10, 1:200), anti-TOX-eFluor660 (TRX19, 1:100) (Thermo Fisher), anti-CD38-AlexaFluor700 (HIT2, 1:400), anti-CD19-APC-eF780 (HIB19, 1:400), anti-CD14-APC-eF780 (61D3, 1:400), anti-IFNγ-FITC (25723.11, 1:50), anti-CD4 (RPA-T4, 1:500), anti-TNF-α-PE-Cy7 (MAb11, 1:400) (BD Bioscience), anti-IL-2-PerCP-Cy5.5 (MQ1-17H12, 1:100). For live/dead discrimination a fixable Viability Dye (APC-eFluor780 1:200) was used. All antibodies were purchased from BD Biosciences (Germany), Thermo Scientific and Cell Signaling Technology (Danvers, MA). FoxP3/Transcription Factor Staining Buffer Set (Thermo Fisher) and Fixation/Permeabilization Solution Kit

(BD Biosciences) were used according to the manufacturer's protocol to stain for intranuclear and cytoplasmic molecules, respectively. After fixation of cells in 2 % para- formaldehyde (PFA, Sigma), analyses were performed on a FACSCanto II or LSRFortessa with FACSDiva software version 10.6.2 (BD). Data were analysed with FlowJo 10.6.2 (Treestar). Gating strategy is displayed in **Fig. S5.**

Dimensional reduction of multiparametric flow cytometry data

Tetramer-positive CD8+ T cells were defined using FlowJo 10. Only samples with ≥ 5 HDV-specific CD8+ T cells were included in the dimensionality reduction analysis. Dimensionality reduction of flow cytometry data was performed with R version 4.5.0 using the Bioconductor CATALYST package (version 1.32.0). The analyses were performed on gated virus-specific CD8+ T cells including the markers CD38, CD39, PD1, TCF-1, BCL-2, CD127, CD45RA, CCR7, EOMES, TIGIT, KLRG1, 2B4, T-BET and TOX. Downsampling of cells to 100 or 200 cells (t-SNE or diffusion maps) was performed before dimensionality reduction to facilitate the visualization of different samples.

Statistics

Statistical analysis was performed with GraphPad Prism 10 (GraphPad Prism Software). Statistical significance was assessed by Mann-Whitney test, nonparametric Spearman correlation, or Wilcoxon matched-pairs signed rank test as indicated. Analyses were performed in independent experiments.

References for Supplementary methods

1 Karimzadeh H, Kiraithe MM, Kosinska AD, Glaser M, Fiedler M, Oberhardt V*, et al.* Amino acid substitutions within HLA-B*27-restricted T cell epitopes prevent recognition by hepatitis delta virus-specific CD8+ T cells. J Virol 2018;**92**.

2 Price DA, Brenchley JM, Ruff LE, Betts MR, Hill BJ, Roederer M*, et al.* Avidity for antigen shapes clonal dominance in CD8+ T cell populations specific for persistent DNA viruses. J Exp Med 2005;**202**:1349-61.

3 Alanio C, Lemaitre F, Law HK, Hasan M, Albert ML. Enumeration of human antigen-specific naive CD8+ T cells reveals conserved precursor frequencies. Blood 2010;**115**:3718-25.

Table S1: Patients' characteristics

ID	Age	Sex	Country of birth	Liver elastography (kPa)	HDV RNA (log10)	ALT (U/L)	Child -Pugh Score	Oesoph. varices	Bilirubin (mg/dl)	Virological response	Bio-chemical response	HLA-A1	HLA-A2	HLA-B1	HLA-B2	Epitope repertoire analysis	T-cell pheno-typing
P1	62	M	caucasian	7.8	4.64	99	A5	yes	1.4	yes	yes	32:01	33:01	14:02	51:01	yes	
P2	40	F	caucasian	24.0	4.34	113	A6	yes	1.5	yes	no	02:01	03:01	35:03	51:01	yes	
P3	62	M	caucasian	39.8	6.22	80	A6	yes	1.4	no	no	24:02	32:01	39:01	50:01	yes	
P4	40	F	caucasian	14.6	3.31	51	A5	yes	0.5	yes	yes	23:01	24:02	44:03	51:01	yes	
P5	70	F	caucasian	16.5	4.58	155	A6	no	1	yes	yes	02:01	33:01	14:02	44:02	yes	
P6	29	M	caucasian	10.9	6.32	133	A5	yes	1	no	no	02:01	24:02	18:01	35:03	yes	
P7	49	M	caucasian	11.6	3.95	86	A5	yes	1.7	no	yes	11:01	24:02	35:02	35:03	yes	
P8	57	F	caucasian	9.4	5.61	49	A5	no	0.9	yes	yes	01:01	11:01	35:01	41:01	yes	yes
P9	34	F	caucasian	16.3	4.39	84	A5	yes	1.4	no	no	02:01	24:02	14:02	51:01	yes	
P10	54	M	caucasian	33.3	6.60	222	A6	yes	1	yes	no	01:01	03:01	51:01	57:01	yes	
P11	68	M	caucasian	15.5	6.27	105	A5	yes	1.5	no	yes	31:01	68:01	18:01	44:03	yes	
P12	77	M	caucasian	15.2	4.39	145	A5	no	0.5	yes	yes	03:01	11:01	35:01	35:03	yes	yes
P13	59	M	caucasian	57.8	5.87	112	A5	yes	1.4	yes	yes	31:01	32:01	18:01	39:06	yes	
P14	47	F	caucasian	17.4	3.74	32	B7	yes	1.8	no	yes	02:01	02:06	15:01	48:01	yes	
P15	37	F	caucasian	22.3	6.27	72	A5	no	0.8	yes	yes	02:01		18:31	44:03	yes	
P16	67	F	tunisian	54.5	6.93	191	A6	yes	0.8	yes	yes	23:01	30:02	50:01	50:02	yes	
P17	56	M	egyptian	41.2	5.70	340	A5	yes	1.2	yes	yes	02:05	68:01	50:01	57:02	yes	
P18	38	F	caucasian	21.2	6.51	197	B7-8	yes	2.9	yes	yes	31:01	68:01	40:01	55:01	yes	
P19	51	M	caucasian	32.8	2.76	52	A6	no	0.9	no	no	02:01	24:02	51:01		yes	
P20	36	F	caucasian	16.5	2.43	51	B8	yes	2.1	no	yes	01:01	03:01	14:01	44:02	yes	
P21	50	M	caucasian	33.2	5.43	77	C10	yes	2.43	yes	no	02:01		07:02	35:01	yes	yes
P22	60	F	caucasian	26.2	5.22	71	A6	yes	1.19	yes	yes	11:01	24:02	40:01	51:01	yes	
P23	44	M	caucasian	ND	2.90	32	A5	yes	0.55	no	yes	02:01	03:01	07:02	40:01	yes	
P24	47	F	caucasian	ND	5.39	103	B8	yes	1	no	yes	02:01		41:01	51:01	yes	
P25	40	M	caucasian	8.9	5.58	172	A5	yes	0.6	yes	no	02:01		35:01	44:02		yes
P26	58	F	caucasian	53.1	5.56	44	A5	yes	0.9	no	no	01:01	11:01	15:02	37:01		yes
P27	59	M	caucasian	62.1	5.35	71	B7	yes	1.9	yes	no	01:01	02:01	35:01	49:01		yes
P28	53	F	caucasian	9.6	4.75	30	A5	no	0.7	no	no	01:01	11:01	07:02	15:01		yes
P29	68	M	caucasian	12.3	1.11	34	A5	no	0.9	no BLV therapy		01:01	02:01	08:01	27:02		yes
P30	41	M	caucasian	8.1	4.52	89	no cirrhosis	no	0.5	no BLV therapy		01:01	33:03	08:01	52:01		yes
P31	43	M	caucasian	10.0	1.00	35	no cirrhosis	no	1.7	no BLV therapy		01:01	02:01	08:01	15:01		yes
P32	38	F	caucasian	4.9	4.38	36	no cirrhosis	no	0.4	no BLV therapy		01:01	02:01	08:01	15:01		yes
P33	56	F	caucasian	ND	4.74	156	B8	ND	2.0	no BLV therapy		02:01		07:02	15:01		yes
P34	46	M	caucasian	5.1	3.95	73	no cirrhosis	no	ND	no BLV therapy		02:01	03:01	07:02	15:01		yes
P35	32	F	caucasian	9.7	6.72	90	no cirrhosis	no	0.6	no BLV therapy		24:02		15:01	35:02		yes
P36	37	F	caucasian	5.9	4.69	33	no cirrhosis	no	0.5	no BLV therapy		01:01	02:01	08:01	15:01		yes
P37	25	M	caucasian	ND	7.72	570	no cirrhosis	no	0.7	no BLV therapy		02	68	13	15:01		yes
P38	55	F	caucasian	6.7	3.18	35	no cirrhosis	no	0.4	no BLV therapy		01:01	32:01	14:01	15:01		yes
P39	30	F	african	4.5	1.40	62	no cirrhosis	no	0.3	no BLV therapy		30:01	34:02	35:01	42:01		yes
P40	61	M	caucasian	5.4	1.00	35	no cirrhosis	no	0.9	no BLV therapy		11:01	24:02	35:01	58:01		yes

Table S2: Overlapping peptides and pools covering L-HDAg

Peptide name	Pool	Sequence	Length	Position in L-HDAg
HDV-OLP-1	pool 1	MSRSESKKNRGGREE	15	1-15
HDV-OLP-2		ESKKNRGGREEVLEQ	15	5-19
HDV-OLP-3		NRGGREEVLEQWVNG	15	9-23
HDV-OLP-4		REEVLEQWVNGRKKL	15	13-27
HDV-OLP-5	pool 2	LEQWVNGRKKLEELE	15	17-31
HDV-OLP-6		VNGRKKLEELERDLR	15	21-35
HDV-OLP-7		KKLEELERDLRKVKK	15	25-39
HDV-OLP-8		ELERDLRKVKKKIKK	15	29-43
HDV-OLP-9	pool 3	DLRKVKKKIKKLEDD	15	33-47
HDV-OLP-10*		VKKKIKKLEDDNPWL	15	37-51
HDV-OLP-11		IKKLEDDNPWLGNIK	15	41-55
HDV-OLP-12		EDDNPWLGNIKGILG	15	45-59
HDV-OLP-13	pool 4	PWLGNIKGILGKKDK	15	49-63
HDV-OLP-14		NIKGILGKKDKDGEG	15	53-67
HDV-OLP-15		ILGKKDKDGEGAPPA	15	57-71
HDV-OLP-16		KDKDGEGAPPAKRAR	15	61-75
HDV-OLP-17	pool 5	GEGAPPAKRARTDQM	15	65-79
HDV-OLP-18		PPAKRARTDQMEVDS	15	69-83
HDV-OLP-19		RARTDQMEVDSGPRK	15	73-87
HDV-OLP-20		DQMEVDSGPRKRPLR	15	77-91
HDV-OLP-21	pool 6	VDSGPRKRPLRGGFT	15	81-95
HDV-OLP-22		PRKRPLRGGFTDKER	15	85-99
HDV-OLP-23		PLRGGFTDKERQDHR	15	89-103
HDV-OLP-24		GFTDKERQDHRRRKA	15	93-107
HDV-OLP-25	pool 7	KERQDHRRRKALENK	15	97-111
HDV-OLP-26		DHRRRKALENKKKQL	15	101-115
HDV-OLP-27		RKALENKKKQLSAGG	15	105-119
HDV-OLP-28		ENKKKQLSAGGKSLS	15	109-123
HDV-OLP-29	pool 8	KQLSAGGKSLSKEEE	15	113-127
HDV-OLP-30		AGGKSLSKEEEEELK	15	117-131

HDV-OLP-31		SLSKEEEEELKRLTE	15	121-135
HDV-OLP-32		EEEEELKRLTEEDER	15	125-139
HDV-OLP-33	pool 9	ELKRLTEEDERRERR	15	129-143
HDV-OLP-34		LTEEDERRERRVAGP	15	133-147
HDV-OLP-35		DERRERRVAGPPVGG	15	137-151
HDV-OLP-36		ERRVAGPPVGGVNPL	15	141-155
HDV-OLP-37	pool 10	AGPPVGGVNPLEGGS	15	145-159
HDV-OLP-38		VGGVNPLEGGSRGAP	15	149-163
HDV-OLP-39		NPLEGGSRGAPGGGF	15	153-167
HDV-OLP-40		GGSRGAPGGGFVPSM	15	157-171
HDV-OLP-41		GAPGGGFVPSMQGVP	15	161-175
HDV-OLP-42	pool 11	GGFVPSMQGVPESPF	15	165-179
HDV-OLP-43		PSMQGVPESPFTRTG	15	169-183
HDV-OLP-44		GVPESPFTRTGEGLD	15	173-187
HDV-OLP-45		SPFTRTGEGLDIRGN	15	177-191
HDV-OLP-46		RTGEGLDIRGNQGFP	15	181-195
HDV-OLP-47	pool 12	GLDIRGNQGFPWDIL	15	185-199
HDV-OLP-48		RGNQGFPWDILFPSD	15	189-203
HDV-OLP-49		GFPWDILFPSDPPFS	15	193-207
HDV-OLP-50		DILFPSDPPFSPQSC	15	197-211
HDV-OLP-51		PSDPPFSPQSCRPQ	14	201-214

* HDV-OLP-10 data was excluded from analysis due to non-specificity for CHD patients (data not shown)

Table S3: Pre-described optimal epitopes tested in this study

HLA restriction	Position in L-HDAg	Sequence	Reference
B*07	194-202	FPWDILFPA	[4]
B*15	170-179	SMQGVPESPF	[5]
B*18	46-54	DENPWLGNI	[5], [4]
B*35	192-200	QGFPWDILF	[4]
B*35	194-202	FPWDILFPA	[4]
B*41	140-149	RERRVAGPPV	[5]
B*44	46-54	DENPWLGNI	[4]
B*51	192-200	QGFPWDILF	[6] and own data

Patients in this cohort did not express the rare HLA alleles A*02:05, B*27, B*37, B*52, or B*58. Described epitopes restricted by these HLA alleles were thus not tested.

References for Table S3:

4 Kefalakes H, Koh C, Sidney J, Amanakis G, Sette A, Heller T, *et al.* Hepatitis D virus-specific CD8+ T cells have a memory-like phenotype associated with viral immune escape in patients with chronic hepatitis D virus infection. Gastroenterology 2019;**156**:1805-19 e9.

5 Karimzadeh H, Kiraithe MM, Oberhardt V, Salimi Alizei E, Bockmann J, Schulze Zur Wiesch J, *et al.* Mutations in hepatitis D virus allow it to escape detection by CD8+ T cells and evolve at the population level. Gastroenterology 2019;**156**:1820-33.

6 Landahl J, Bockmann JH, Scheurich C, Ackermann C, Matzat V, Heide J, *et al.* Detection of a broad range of low-level major histocompatibility complex class II-restricted, hepatitis delta virus (HDV)-specific T-cell responses regardless of clinical status. J Infect Dis 2019;**219**:568-77.

Table S4: Autologous viral epitope sequences

Patient	Epitope name	Prototype sequence	Autologous viral sequence
P1	HLA-B*51: HDAg192-200	QGFPWDILF	RGFPWDMLF
P2	HLA-B*35/*51: HDAg192-200 (and olp48/49)	QGFPWDILF	RGFPWDILF
P6	HLA-B*18: HDAg46-54	DENPWLGNI	EDNPWLGNI
	HLA-B*35: HDAg192-200	QGFPWDILF	QGFPWDILF
P7	HLA-B*35: HDAg192-200	QGFPWDILF	QGFPWDILF
P8	HLA-B*35: HDAg194-202 (and olp48/49)	FPWDILFPA	FPWDILFPA
P9	HLA-B*51: HDAg194-202 (and olp48/49)	FPWDILFPA	FPWDILFPS
	olp26	DHRRRKALENKKKQL	AHRRRKALENKKKQL
P10	HLA-B*51: HDAg194-202	QGFPWDILF	QGFPWDILF
P11	HLA-B*18/*44: HDAg46-54	DENPWLGNI	DDNPWLGNI
P12	HLA-B*35: HDAg194-202 (and olp48/49)	FPWDILFPA	FPWDILFPS
P13	HLA-B*18: HDAg46-54	DENPWLGNI	EENPWLGNI
P14	HLA-B*15: HDAg170-179	SMQGVPESPF	NMLEIPESPF
	olp45	SPFTRTGEGLDIRGN	SPFSRTGEGLDVRGN
P15	olp4	REEVLEQWVNGRKKL	REETLEQWVSARKKL
	olp6	VNGRKKLEELERDLR	VSARKKLEDLERDLR
P16	olp1	MSRSESKKNRGGREE	MSRAESKKHRGGREE
	olp8	ELERDLRKVKKKIKK	ELERDLRKVKKKIKK
	olp33	ELKRLTEEDERRERR	ELERLTKEDEKRKRR
	olp43	PSMQGVPESPFTRTG	PSMQGVPESPFSRHG
P21	HLA-B*35: HDAg192-200	QGFPWDILF	QGFPWDILF
P25	HLA-B*35: HDAg194-202	QGFPWDILF	RGFPWDILF
P26	HLA-B*37: HDAg100-108	QDHRRRKAL	QEHRRRKAL
P27	HLA-B*35: HDAg194-202	QGFPWDILF	QGFPWDILF
P28	HLA-B*15: HDAg170-179	SMQGVPESPF	SMQGVPESPF
P29	HLA-B*8: HDAg50-58	WLGNIKGIL	WLGNIKGIL
P30	HLA-B*8: HDAg50-58	WLGNIKGIL	WLGNIKGIL
P31	HLA-B*8: HDAg50-58	WLGNIKGIL	WLGNIKGIL
	HLA-B*15: HDAg170-179	SMQGVPESPF	NMQGVPESPF
P32	HLA-B*8: HDAg50-58	WLGNIKGIL	WLGNIKGIL
P33	HLA-B*15: HDAg170-179	SMQGVPESPF	SMQGVPESPF
P34	HLA-B*15: HDAg170-179	SMQGVPESPF	SMQGVPESPF
P35	HLA-B*15: HDAg170-179	SMQGVPESPF	NMQGVPESPF
P36	HLA-B*15: HDAg170-179	SMQGVPESPF	NMQGVPESPF
P37	HLA-B*15: HDAg170-179	SMQGVPESPF	NMQGVPESPF
P38	HLA-B*15: HDAg170-179	SMQGVPESPF	NMQGVPESPF
P39	HLA-B*35: HDAg194-202	QGFPWDILF	QGFPWDILF
P40	HLA-B*35: HDAg194-202	QGFPWDILF	QGFPWDILF

Fig. S1

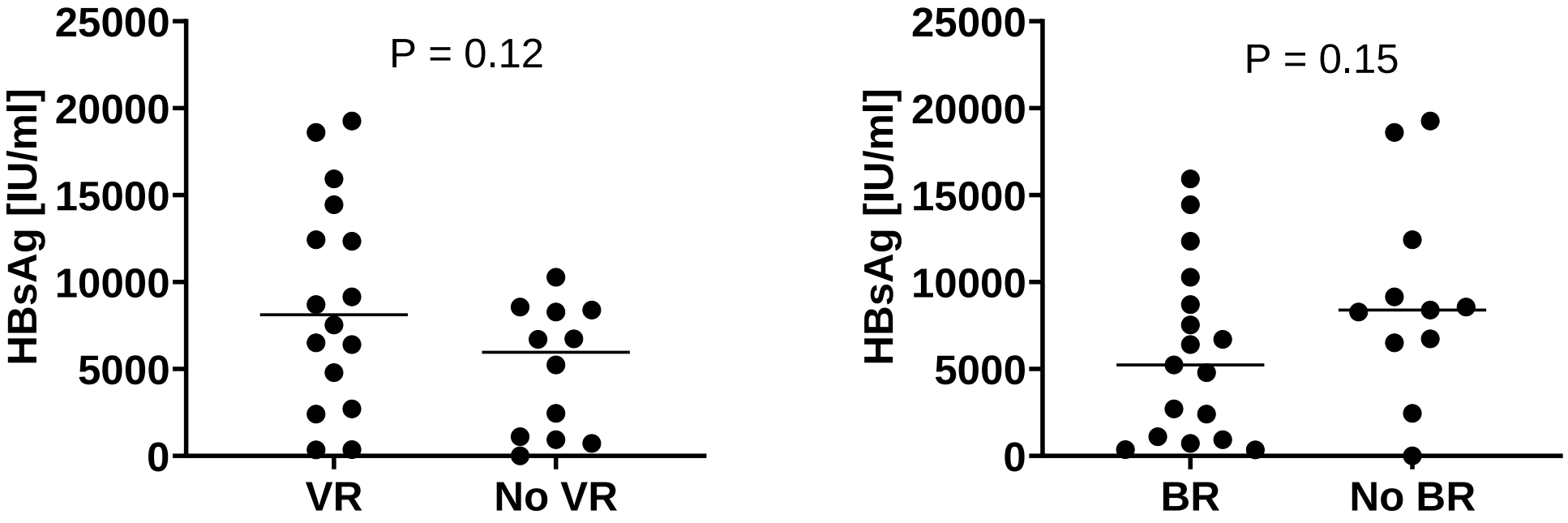

Fig. S1: Comparison of baseline HBsAg levels in patients with or without virological response (left) and in patients with or without biochemical response (right). Statistics: Mann-Whitney test.

Fig. S2

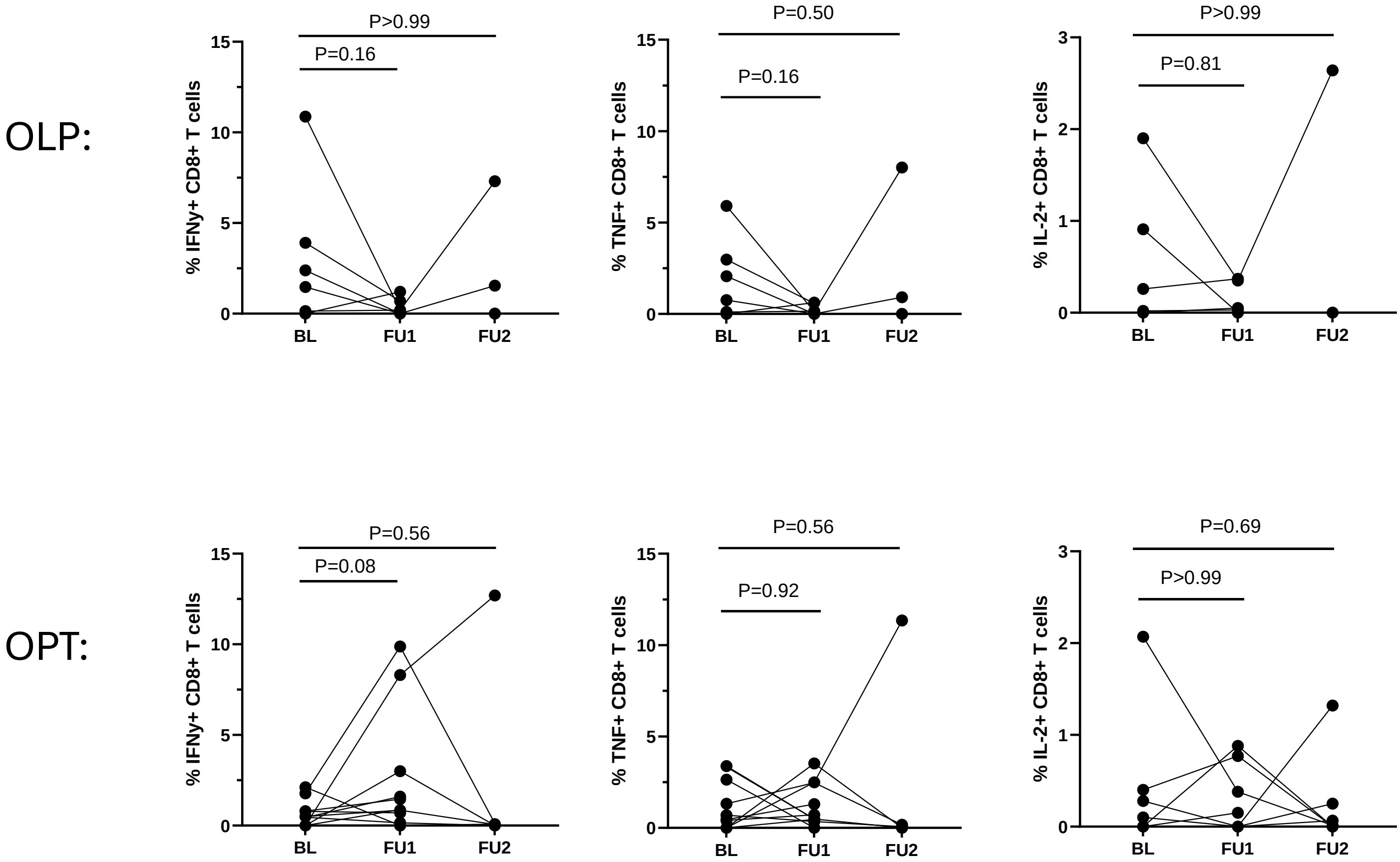

Fig. S2: Longitudinal strength of HDAg-specific CD8+ T-cell responses producing IFNγ, TNF, and IL-2. Results are displayed separately for experiments using OLP (upper row) and optimal epitope peptides (lower row), respectively. Statistics: Wilcoxon matched-pairs signed rank test.

Fig. S3

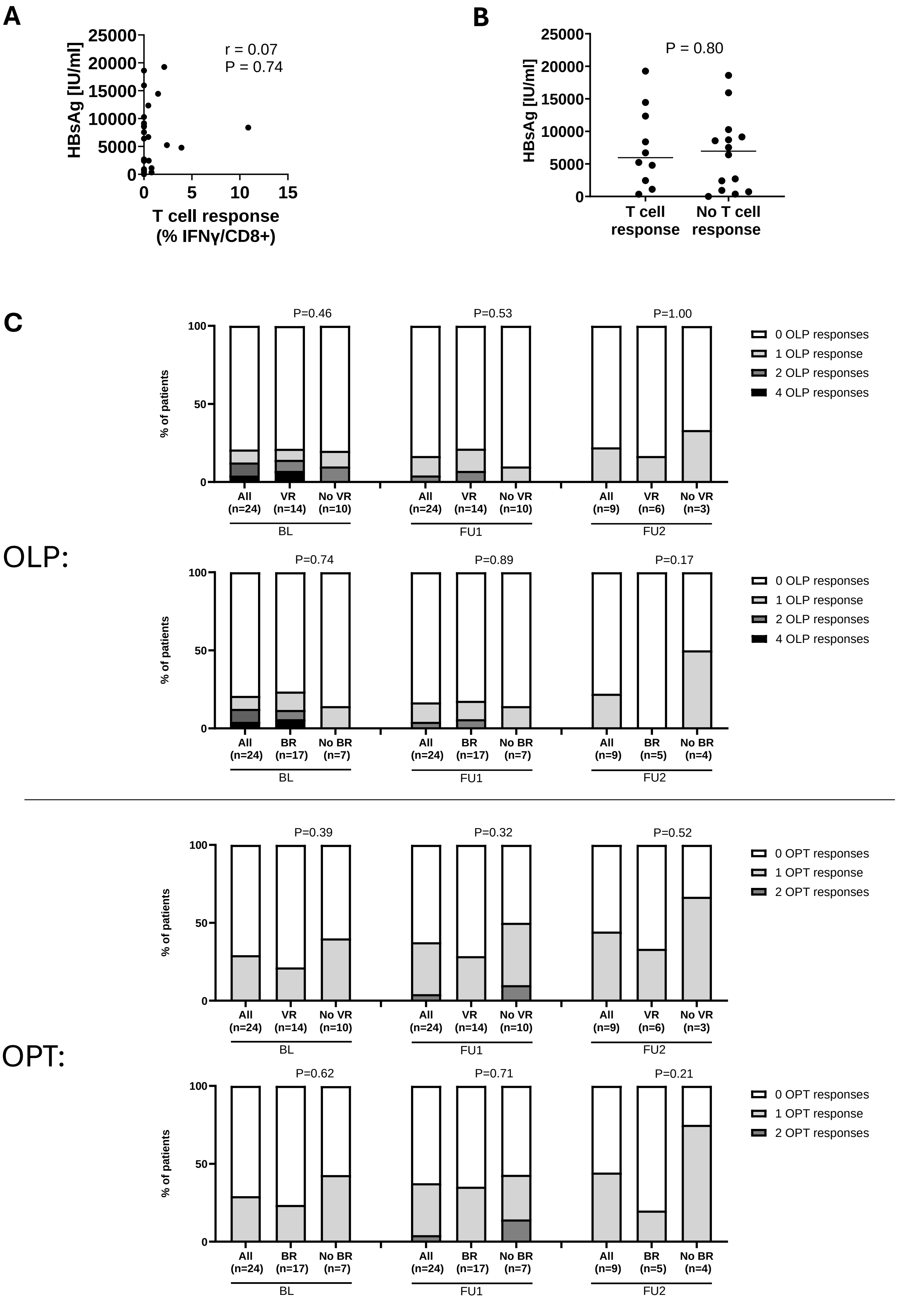
A
HBsAg [IU/ml]
25000
20000
15000
10000
5000
0
0 5 10 15
T cell response
(% IFNγ/CD8+)
r = 0.07
P = 0.74
B
HBsAg [IU/ml]
P = 0.80
T cell response
No T cell response
C
OLP:
P=0.46
P=0.53
P=1.00
% of patients
100
50
0
All (n=24)
VR (n=14)
No VR (n=10)
BL
All (n=24)
VR (n=14)
No VR (n=10)
FU1
All (n=9)
VR (n=6)
No VR (n=3)
FU2
0 OLP responses
1 OLP response
2 OLP responses
4 OLP responses
P=0.74
P=0.89
P=0.17
All (n=24)
BR (n=17)
No BR (n=7)
BL
All (n=24)
BR (n=17)
No BR (n=7)
FU1
All (n=9)
BR (n=5)
No BR (n=4)
FU2
OPT:
P=0.39
P=0.32
P=0.52
0 OPT responses
1 OPT response
2 OPT responses
P=0.62
P=0.71
P=0.21

Fig. S3 (continued)

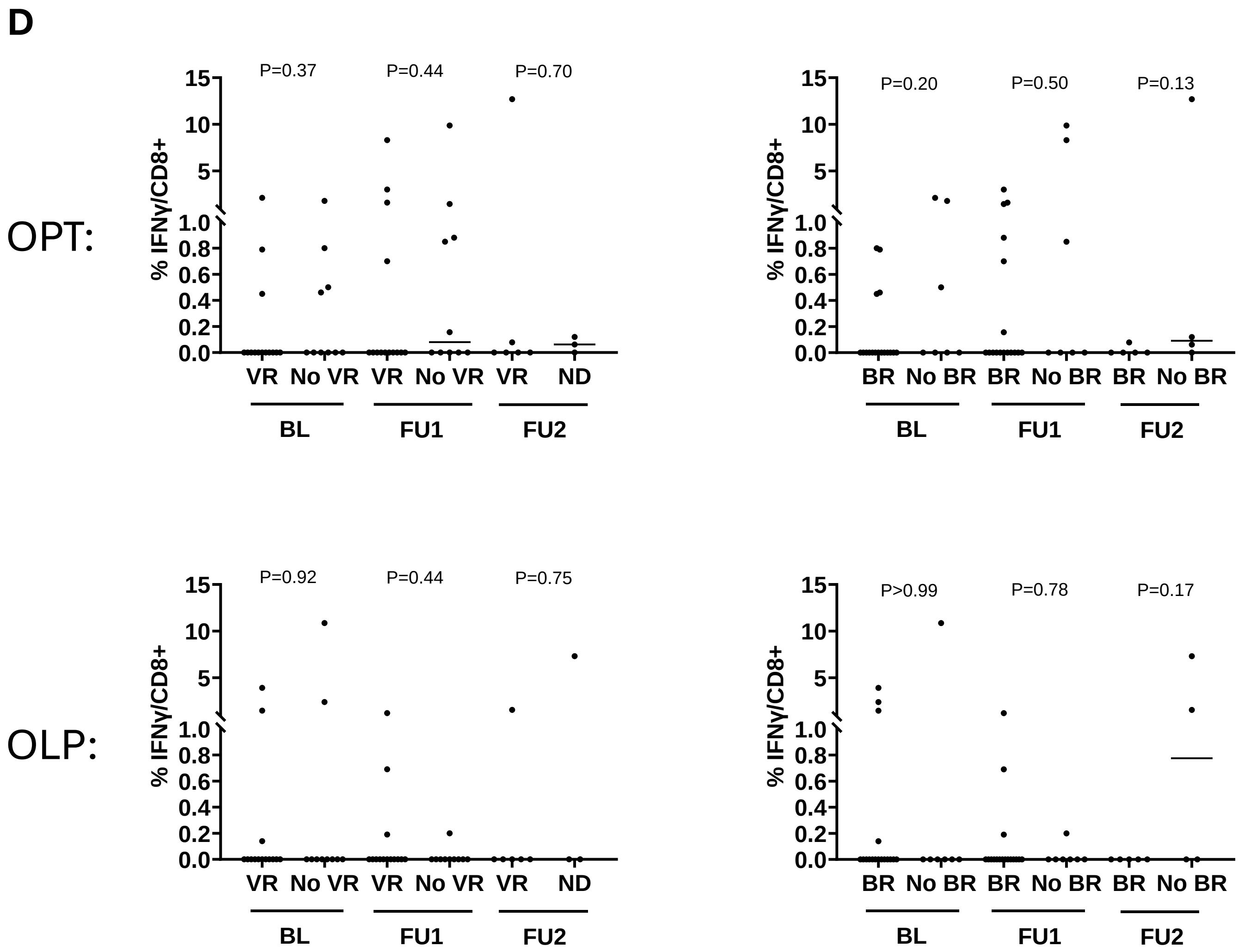

Fig. S3: Correlation of the HDAg-specific CD8+ T-cell response with baseline virological and biochemical data and treatment response.

A Presence or absence of HDAg-specific CD8+ T-cell responses was correlated with baseline HBsAg levels. **B** Strength of HDAg-specific CD8+ T-cell responses was correlated with baseline HBsAg levels. **C and D** The longitudinal number (C) and strength (D) of HDAg-specific CD8+ T-cell responses is displayed for patients with or without virological response (VR) and with or without biochemical response (BR), respectively. Responses detected using OLPs and optimal epitope peptides are displayed separately. Statistics: A, C, D: Mann-Whitney test; B: nonparametric Spearman correlation.

Fig. S4

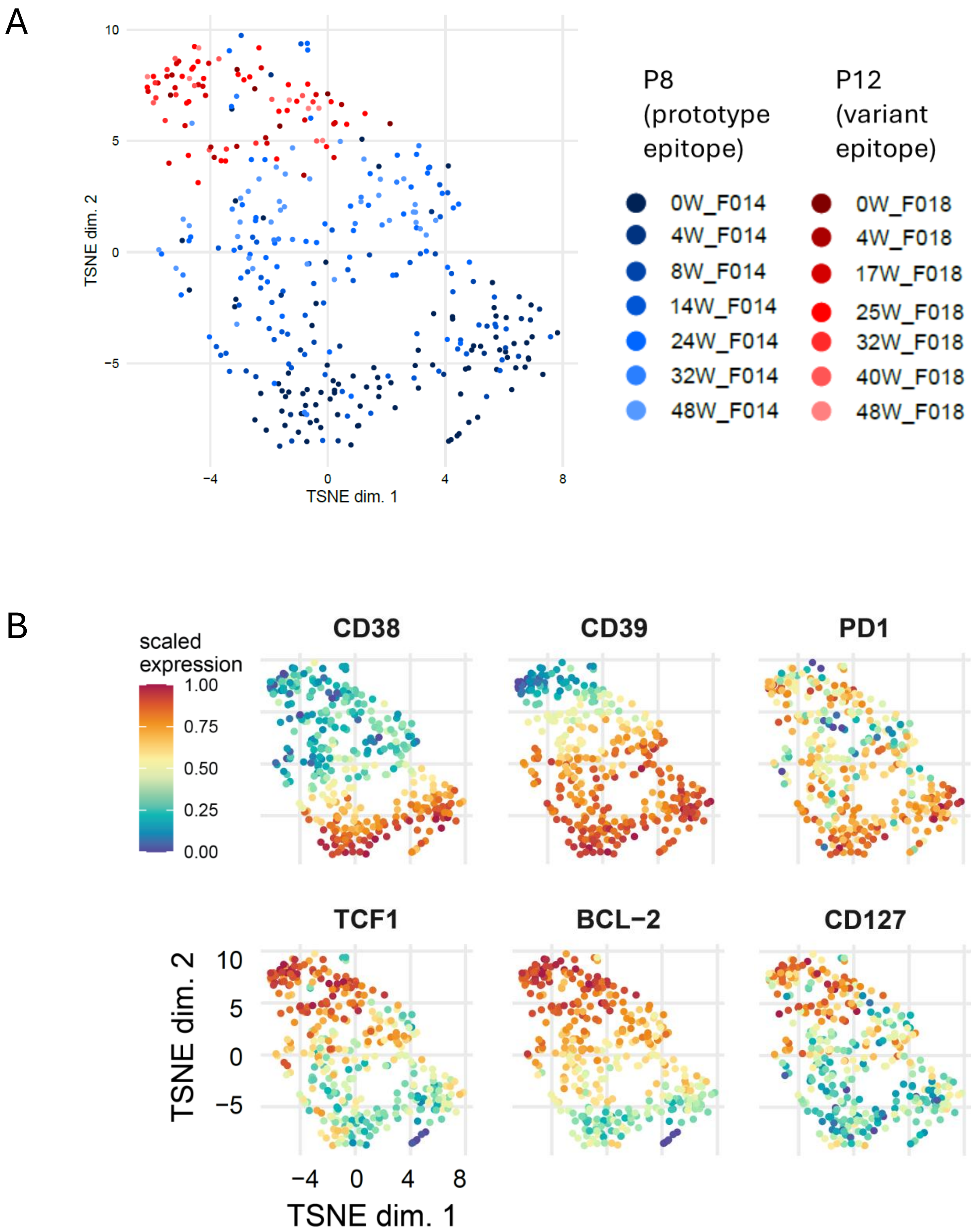
A
10
5
0
−5
TSNE dim. 2
−4
0
4
8
TSNE dim. 1
P8
(prototype
epitope)
P12
(variant
epitope)
0W_F014
4W_F014
8W_F014
14W_F014
24W_F014
32W_F014
48W_F014
0W_F018
4W_F018
17W_F018
25W_F018
32W_F018
40W_F018
48W_F018
B
scaled
expression
1.00
0.75
0.50
0.25
0.00
CD38
CD39
PD1
TCF1
BCL−2
CD127
10
5
0
−5
TSNE dim. 2
−4
0
4
8
TSNE dim. 1

Fig. S4 (continued)

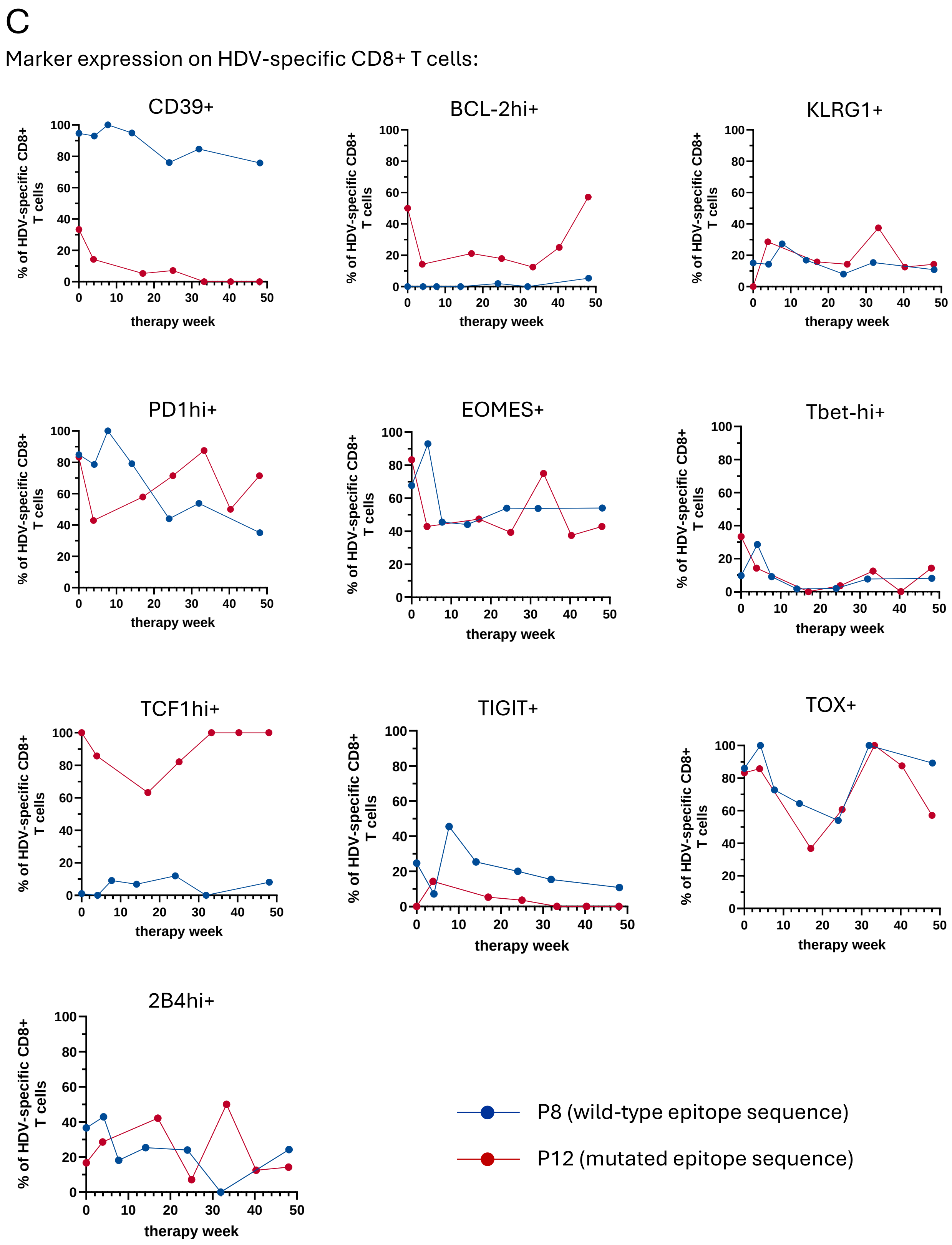

Fig. S4 (continued)

D

Marker expression on **PD1+/CD127+** HDV-specific CD8+ T cells:

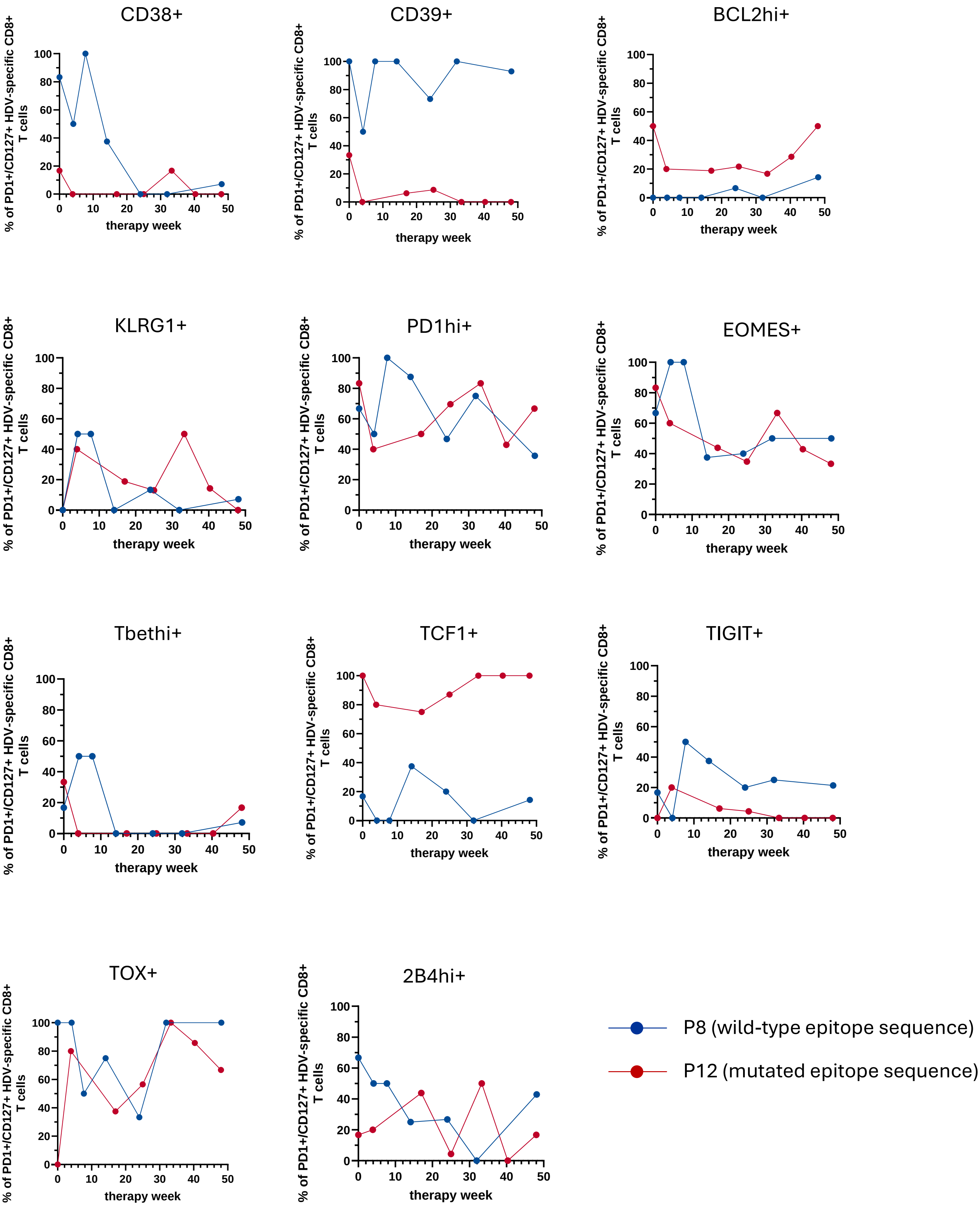

Fig. S4 (continued)

E

Marker expression on **PD1+/CD127-** HDV-specific CD8+ T cells

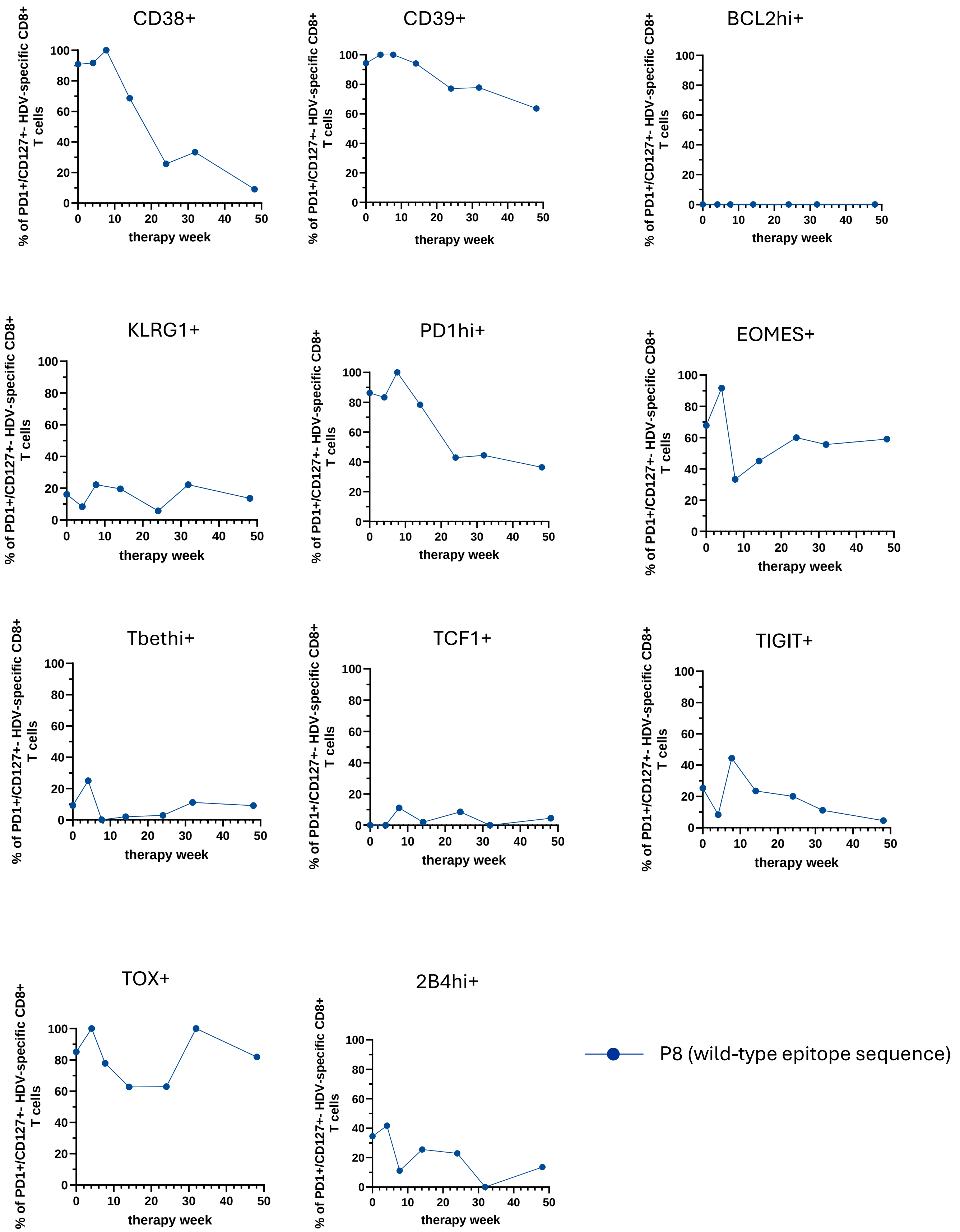

Fig. S4 (continued)

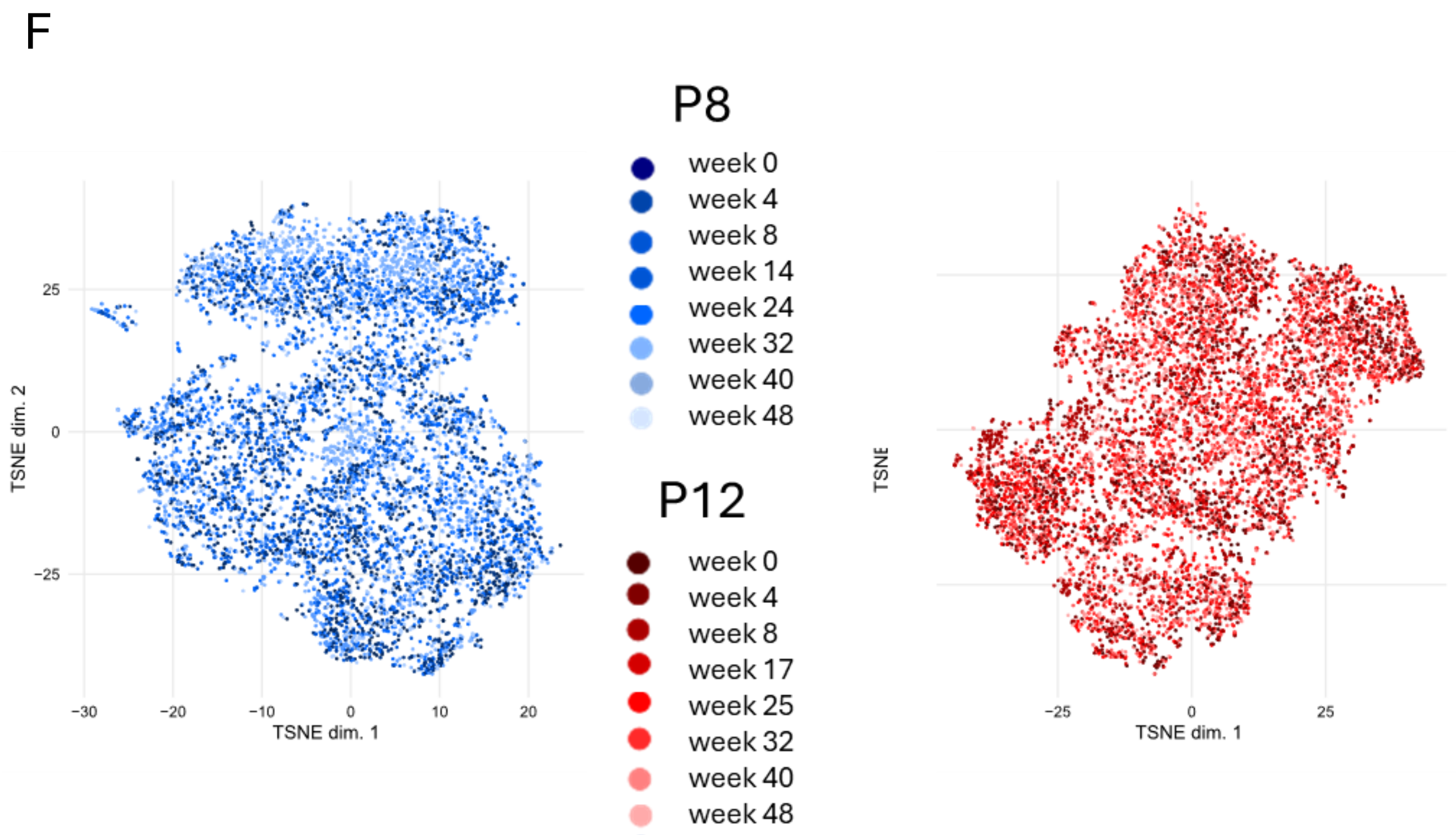

Fig. S4: BLV therapy ameliorates HDV-specific CD8+ T cells targeting epitopes with prototype viral sequence only.

A t-SNE analysis of HDAg192-200-specific CD8+ T cells from the two patients (P8 with prototype viral epitope sequence and P12 with variant viral epitope sequence) from multiple time points during 48 weeks of BLV treatment (compare Fig. 4B, where only baseline and week 48 are displayed).

B Scaled expression of single markers.

C Longitudinal percentage of marker-positive HDAg192-200-specific CD8+ T cells in patients P8 and P12.

D Longitudinal percentage of marker-positive PD1+CD127+ HDAg192-200-specific CD8+ T cells in patients P8 and P12.

E Longitudinal percentage of marker-positive PD1+CD127- HDAg192-200-specific CD8+ T cells in patients P8 (P12 is not shown, since in this patient, the number of PD1+CD127- was not sufficient for phenotypic analysis at several time-points).

F t-SNE analysis of global CD8+ T cell population from patients P8 and P12 from multiple time points during BLV treatment.

Fig. S5

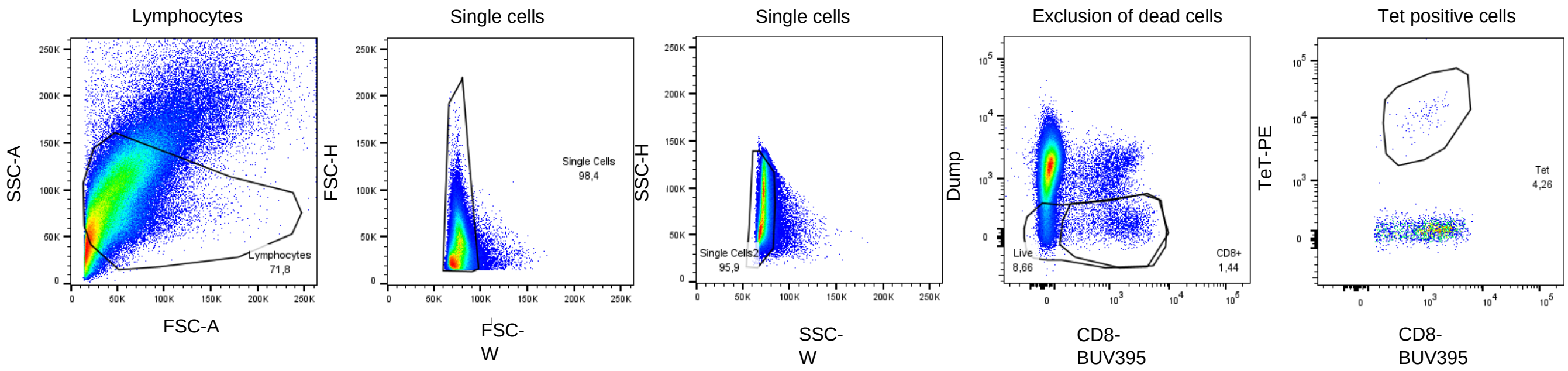

Fig. S5: Gating strategy for phenotypical analysis of tetramer-positive CD8+ T cells.
