# Supplementary material for: Fate of hepatitis D virus-specific CD8+ T cells during bulevirtide monotherapy in patients with chronic hepatitis delta
Source: JHEP Rep. 2026 Jul 24;8(10):101970. doi: 10.1016/j.jhepr.2026.101970 (PMC13594906; doi:10.1016/j.jhepr.2026.101970)
Supplement: Supplementary file 3 [file mmc4.pdf]

Research article

JHEP|Reports

Fate of hepatitis D virus-specific CD8+ T cells during bulevirtide monotherapy in patients with chronic hepatitis delta

Authors

Valerie Oberhardt, Elisabetta Degasperi, Michelle Maas, …, Robert Thimme, Pietro Lampertico, Christoph Neumann-Haefelin

Correspondence

christoph.neumann-haefelin@uk-koeln.de (C. Neumann-Haefelin).

Graphical abstract

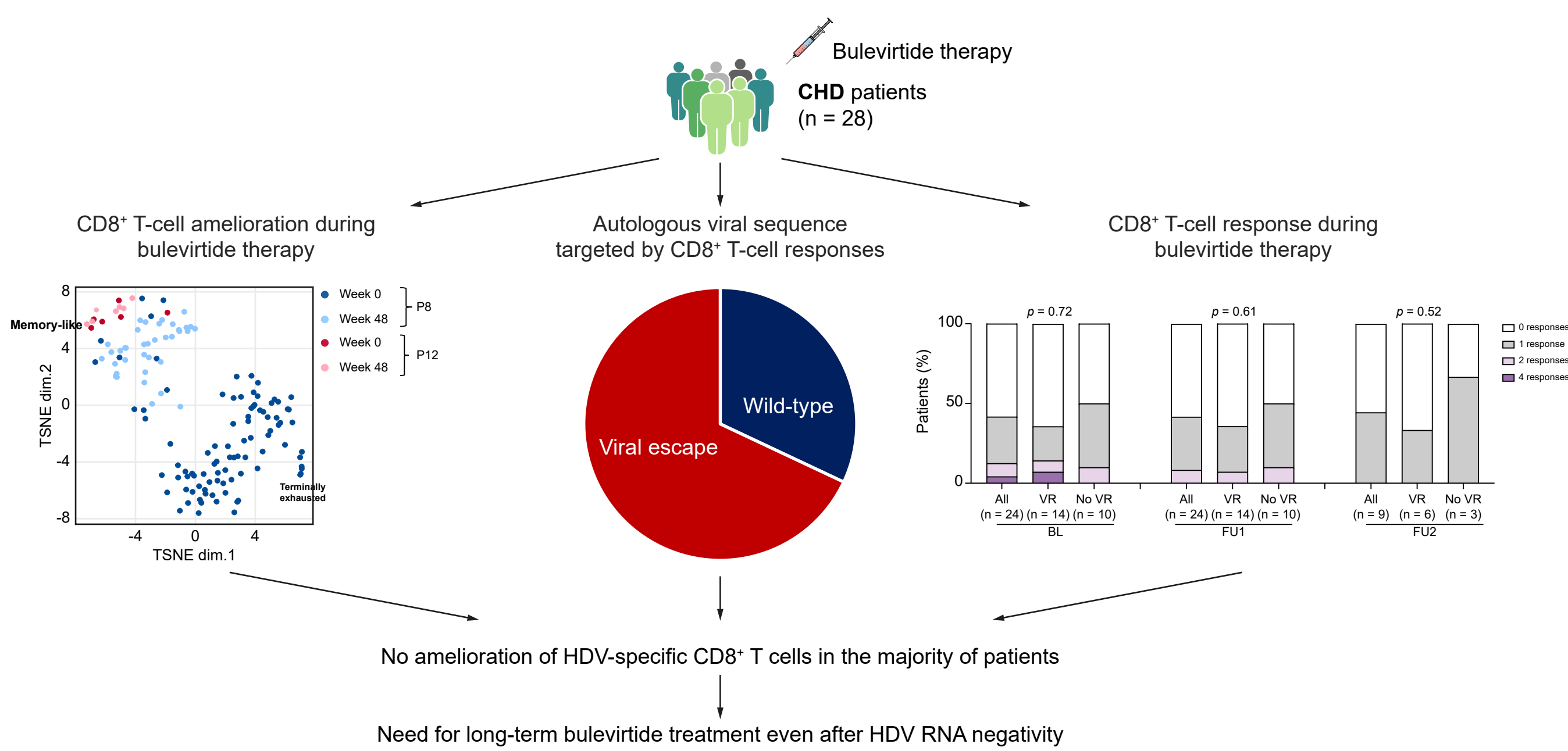

Highlights:

- During bulevirtide treatment, HDV-specific CD8+ T cells targeting conserved HDV epitopes are phenotypically ameliorated during antigen load reduction.
- However, the majority of HDV-specific CD8+ T-cell responses target viral epitopes with mutational viral escape.
- These results suggest that HDV-specific T cells may not contribute to prevent HDV relapse after bulevirtide discontinuation.
- Thus, long-term treatment may be needed even after achievement of HDV RNA negativity.

Impact and implications:

Bulevirtide can control viremia and normalize liver enzymes in a majority of patients with chronic hepatitis delta, but sustained cure with negative HDV RNA even after therapy is difficult to achieve. Restoration of functional HDV-specific CD8+ T cells attributable to reduced antigen loads may prevent viral relapse. We demonstrate here that amelioration of HDV-specific CD8+ T cells does indeed occur, but is limited to the few HDV-specific CD8+ T-cell responses targeting conserved viral epitopes. A majority of HDV-specific CD8+ T cells no longer recognize their target antigens because of mutational viral escape. These findings explain the need for long-term treatment continuation even once HDV RNA is negative, to prevent viral relapse.

https://doi.org/10.1016/j.jhepr.2026.101970

© 2026 The Author(s). Published by Elsevier B.V. on behalf of European Association for the Study of the Liver (EASL). This is an open access article under the CC BY license (http://creativecommons.org/licenses/by/4.0/). JHEP Reports, 2026, 8, 1–11

Fate of hepatitis D virus-specific CD8+ T cells during bulevirtide monotherapy in patients with chronic hepatitis delta☆

Valerie Oberhardt[1,†], Elisabetta Degasperi[2,†], Michelle Maas[1,3,4,†], Marta Borghi[2], Kathrin Heim[1], Roberta Soffredini[2], Maria Paola Anolli[2], Floriana Facchetti[2], Stéphanie Narguet[5], Tarik Asselah[5], Elahe Salimi Alizei[1], Özlem Sogukpinar[1], Frances Winkler[1], Sian Llewellyn-Lacey[6], Florian Emmerich[7], David A. Price[6,8], Tobias Boettler[1], Bertram Bengsch[1,9], Hendrik Luxenburger[1], Maike Hofmann[1], Robert Thimme[1], Pietro Lampertico[2,10,11,#], Christoph Neumann-Haefelin[1,4,12,13,*,#]

JHEP Reports **2026**. vol. 8 | 1–11

Check for updates

Background & Aims: Bulevirtide reduces viremia in chronic hepatitis D virus (HDV) infection, but long-term treatment is required in most patients to prevent relapse. Sustained treatment responses may be fostered by therapy-induced amelioration of HDV-specific CD8+ T-cell responses that are exhausted because of high viremia and antigen loads during chronic HDV infection. We therefore studied the effect of bulevirtide monotherapy on HDV-specific CD8+ T-cell repertoire, phenotype, and functionality.

Methods: Twenty-eight patients with HDV infection and cirrhosis starting bulevirtide treatment were followed for 40–120 weeks on treatment. HLA class I typing and HDV sequencing was performed. HDV-specific CD8+ T cells were analyzed longitudinally using optimal epitopes and overlapping peptides spanning the L-HDAg. *Ex vivo* high-dimensional flow cytometry analysis of peptide/HLA class I tetramer+ CD8+ T cells was performed longitudinally in selected patients.

Results: In 42% of patients, an HDV-specific CD8+ T-cell response was detectable at baseline, but did not substantially increase during treatment. Importantly, a large majority of HDV-specific CD8+ T-cell responses targeted viral epitopes with sequence variations consistent with viral escape mutations. Only one HLA-B*35-restricted HDV-specific CD8+ T-cell epitope was conserved and continuously targeted throughout therapy. HDV-specific CD8+ T cells targeting this conserved epitope displayed a predominant terminally exhausted phenotype at baseline that shifted longitudinally to a memory-like phenotype with suppression of HDV load.

Conclusions: Albeit based on small patient numbers, antiviral treatment with bulevirtide could ameliorate exhausted HDV-specific CD8+ T cells targeting conserved HDV epitopes. However, as the majority of HDV-specific CD8+ T-cell responses target escaped viral epitopes, this does not translate to a substantial improvement in overall HDV-specific CD8+ T-cell immunity. Our findings may explain in part why long-term bulevirtide treatment is required to prevent viral relapse.

© 2026 The Author(s). Published by Elsevier B.V. on behalf of European Association for the Study of the Liver (EASL). This is an open access article under the CC BY license (http://creativecommons.org/licenses/by/4.0/).

Introduction

HDV is a small defective hepatotropic RNA virus that depends on the HBsAg for cell entry into hepatocytes. HDV superinfection of individuals who are HBV-positive mostly results in chronic hepatitis delta, leading to cirrhosis in ∼75% of patients within 15 years.[1] Until 2020, off-label pegylated interferon-alpha (pegIFNα) was the only therapeutic option for patients with chronic hepatitis delta. Treatment with pegIFNα, however, results in sustained virological response in only ∼20% and is contraindicated in many patients, including those with advanced cirrhosis. In 2020, the entry inhibitor bulevirtide (BLV), a peptide homologue of the N-terminal HBsAg domain preS1, was approved for the treatment of chronic hepatitis delta in Europe. BLV leads to a decline of HDV viremia as well as normalization of liver enzymes in the majority of patients. Indeed, ∼70% of treated patients display a virological response, defined as an at least two log decline of HDV RNA, and a biochemical response, defined as normalization of alanine aminotransferase (ALT) levels, respectively, resulting in a combined response (virological + biochemical response) in ∼50% of patients.[2–4] While treatment response continues to increase with treatment duration, only a smaller part of patients become completely negative for HDV RNA after a treatment duration of up to 3 years, and even these patients remain at

☆ Given their role as Associate Editor, Tobias Boettler had no involvement in the peer-review of this article and had no access to information regarding its peer-review. Full responsibility for the editorial process for this article was delegated to the Editor-in-Chief Josep M. Llovet.

* Corresponding author. Address: Department of Gastroenterology and Hepatology, University Hospital Cologne, Kerpener Strasse 68, D-50937, Cologne, Germany.
E-mail address: christoph.neumann-haefelin@uk-koeln.de (C. Neumann-Haefelin).
† These first authors contributed equally.
# These last authors contributed equally.

https://doi.org/10.1016/j.jhepr.2026.101970

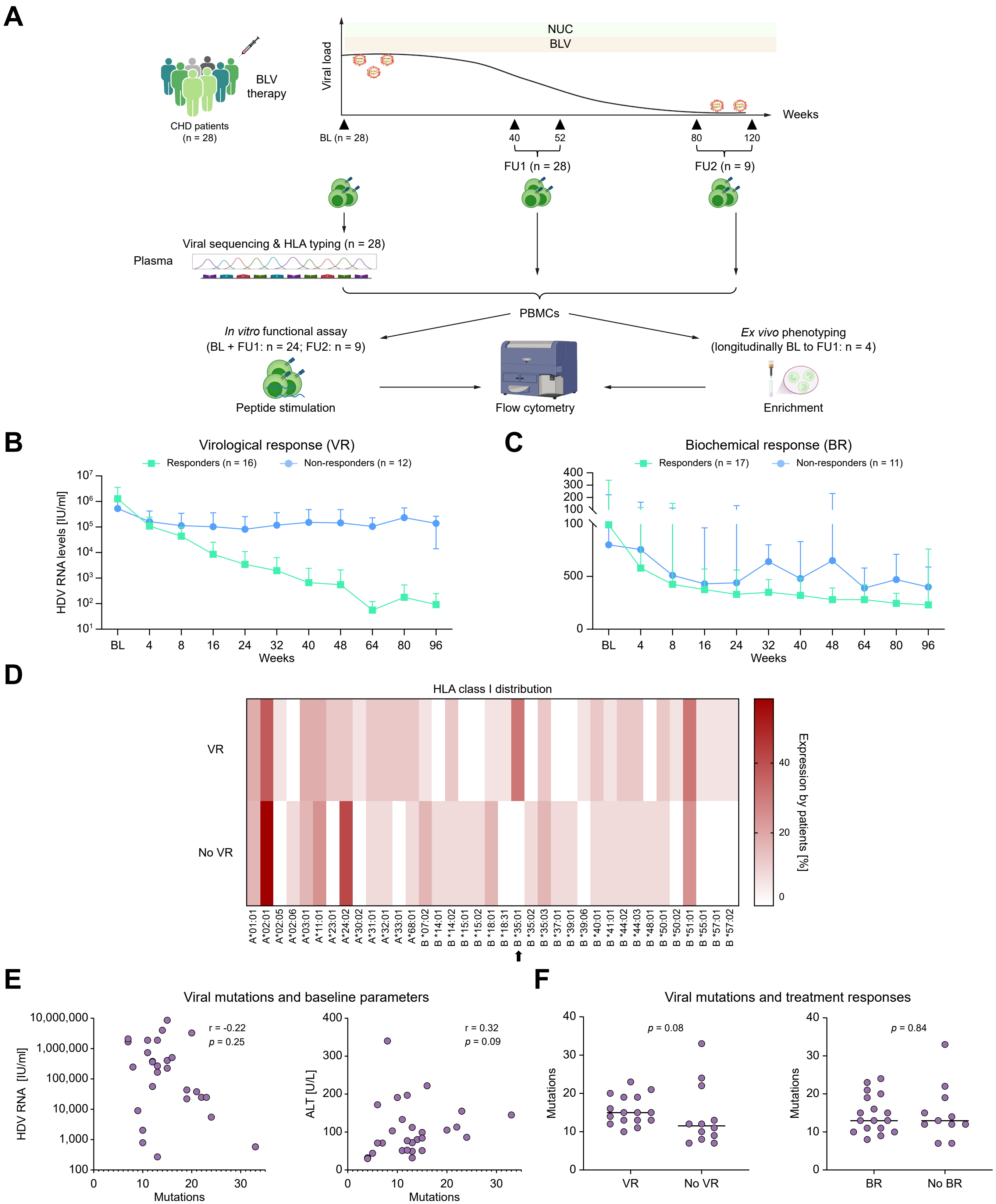

Fig. 1. Study design, treatment response, HLA class I, and viral sequence data in a cohort of patients with cirrhosis with chronic hepatitis delta and BLV therapy. (A) Study design. (B) Longitudinal HDV RNA levels in patients with virological response or non-response. (C) Longitudinal ALT levels in patients with biochemical response or non-response. (D) HLA class I distribution in patients with virological response *vs.* virological non-response. (E) Correlation of the number of HDV amino acid variations compared with consensus with HDV RNA levels and ALT levels in the respective patient at baseline. (F) Correlation of the number of HDV

high risk of viral relapse after stopping BLV therapy.[5] Thus, long-term treatment with BLV is the current state of the art.[6]

In chronic hepatitis delta, HDV-specific CD8+ T cells are detectable in a subset of patients. Indeed, two studies using overlapping peptides as well as one study mapping HDV-specific CD8+ T cell epitopes in regions with HLA class I-associated sequence variations identified HDV-specific CD8+ T-cell responses in 38.9–70.6% of patients.[7–9] The HDV-specific CD8+ T cell epitopes were largely restricted by HLA-B alleles. Mutational viral escape was observed in a substantial number of epitopes; however, the proportion of HDV-specific CD8+ T-cell responses affected by viral escape has not been defined.[7,8] Of note, HDV-specific CD8+ T cells targeting wild-type epitopes displayed a 'chronically activated' or exhausted phenotype.[7–10] As T-cell exhaustion is thought to be mainly driven by high antigen loads, BLV-induced decline of HDV viremia may result in amelioration of HDV-specific CD8+ T-cell immunity. Reinvigorated HDV-specific CD8+ T cells may in turn foster a sustained virological response to BLV treatment, even off therapy. We thus aimed to study the effect of BLV therapy on the HDV-specific CD8+ T-cell repertoire, phenotype and functionality in a unique cohort of 28 patients with chronic hepatitis delta starting BLV monotherapy.

Patients and methods

A total of 28 patients with cirrhosis caused by chronic hepatitis delta (CHD) starting BLV monotherapy were included in this study. Patients were recruited at the Foundation IRCCS Ca' Granda Ospedale Maggiore Policlinico, Milan, Italy, the Freiburg University Medical Center, Freiburg, Germany, and the University of Paris-Cité, Hôpital Beaujon, Paris, France. All patients were infected with HDV genotype 1. Patient characteristics are summarized in Table S1. Clinical assessment was performed every 4 weeks; sampling for peripheral blood mononuclear cell isolation was performed every 4–8 weeks during the first 24 weeks and then every 8–16 weeks. The last peripheral blood mononuclear cell sampling and analysis occurred after 40–52 (n = 19) or 80–120 (n = 9) weeks of therapy. HLA class I typing was performed by next-generation sequencing. Written informed consent was obtained from all participants before inclusion. The study was conducted according to federal guidelines and local ethics committee regulations (Albert-Ludwigs-Universität, Freiburg, Germany; #23-1458-S1) and the Declaration of Helsinki (1975).

Methods for immunological analyses, viral sequence analysis, and statistics are supplied in the Supplementary materials.

Results

Virological and biochemical response to BLV treatment in a cohort of patients with cirrhosis with CHD

We recruited a cohort of 28 patients with chronic hepatitis delta starting on BLV monotherapy. All patients had compensated cirrhosis, were infected with HDV genotype 1, and had an HBV backbone treatment with entecavir or tenofovir (Table S1). Patients were followed for 40–120 weeks on treatment (Fig. 1A). At baseline, HDV sequence analysis and HLA class I typing was performed. At baseline (n = 24), Week 40–52 (follow-up 1 [FU1], n = 24), and Week 80–120 (follow-up 2 [FU2], n = 9), comprehensive immunological analyses were performed. For analyses of the immunological results, patients were stratified as virological responders (HDV RNA decline >2 log10 or HDV RNA negative at Week 48) or virological non-responders and biochemical responders (ALT normalization at Week 48) or biochemical non-responders (Fig. 1B,C). Of note, virological and biochemical response rates were in the same range as previously reported (57% and 61%, respectively); they were not linked to baseline HBsAg levels (Fig. S1A). HLA class I typing reflected a typical Caucasian population with balanced HLA class I expression between patients with virological response and non-response, with a single exception for HLA-B*35:01 which tended to be overrepresented in virological responders (p = 0.055) (Fig. 1D; HLA-B*35:01 is indicated by an arrow). HDV sequence analysis revealed an expected HDV variability (median 13, range 7–33 of the 214 amino acids of L-HDAg variant from consensus), with no correlation with HDV RNA or ALT levels at baseline (Fig. 1E) and no association with virological or biochemical treatment response (Fig. 1F).

HDV-specific CD8+ T-cell responses at baseline and on BLV treatment

We next studied HDV-specific CD8+ T-cell responses longitudinally at baseline and the two follow-up time points during BLV treatment (FU1 ~1 year on treatment [Week 40–52]; FU2 ~2 years on treatment [Week 80–120]). In a comprehensive analysis, we used (1) 51 overlapping peptides (OLPs; 14–15mers, overlapping by 11 aa) spanning the complete L-HDAg protein (Table S2); and (2) a set of five pre-described and fine-mapped L-HDAg-specific CD8+ T-cell epitopes restricted by seven different HLA class I alleles (Table S3)[7–9] and performed peptide-specific expansion for 14 days followed by intracellular interferon-gamma (IFNγ) staining as a sensitive approach (Fig. 2A). Using OLPs, we detected HDV-specific CD8+ T-cell responses in a minority of patients at baseline (five of 24 patients, 20.8%) (Fig. 2B, upper left panel). In patients with positive responses, one to four (median two) responses were detectable per patient. Strikingly, after 1 and 2 years on treatment, the CD8+ T-cell response was not enhanced as hypothesized, but rather contracted. At FU1, CD8+ T-cell responses were detectable in four of 24 patients (16.7%), with one response each in three of these patients and two responses in the fourth patient (Fig. 2B, middle left panel). At FU2, two of nine (22.2%) tested patients displayed a CD8+ T-cell response with one response each (Fig. 2B, lower left panel). Using HLA class I-matched previously described CD8+ T-cell epitope peptides, we observed CD8+ T-cell responses in seven of 24 (29.1%) patients at baseline (Fig. 2B, upper right panel), nine of 24 (37.5%) patients at FU1 (Fig. 2B, middle right panel), and four of nine (44.4%) patients at FU2 (Fig. 2B, lower right panel). All patients were positive for a single

amino acid variations compared with consensus with virological and biochemical treatment response. Statistics: (E) Non-parametric Spearman correlation; (F) Mann–Whitney *U* test. ALT, alanine aminotransferase; BLV, bulevirtide; FU1, follow-up 1; FU2, follow-up 2; PBMCs, peripheral blood mononuclear cells.

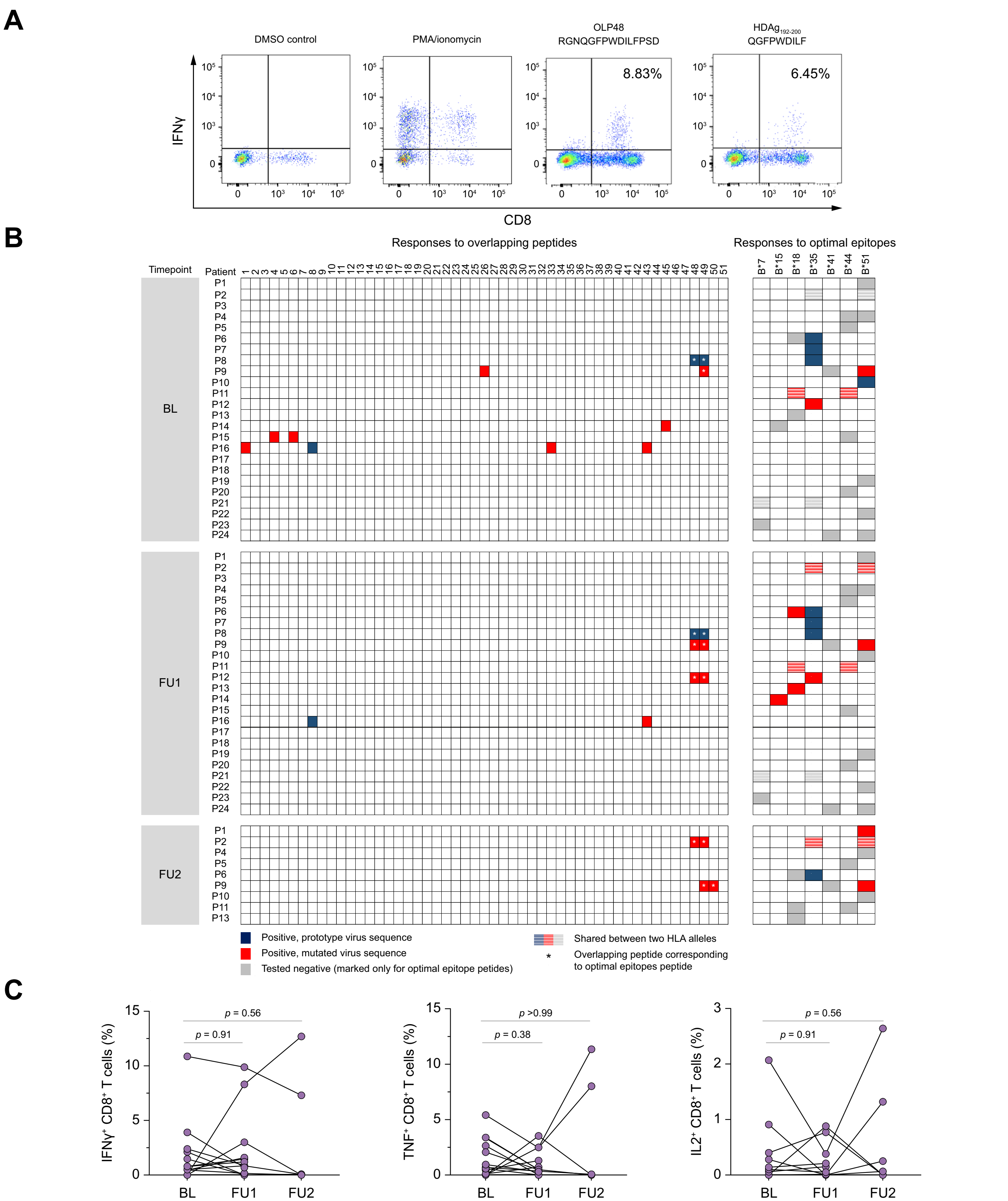

Fig. 2. Comprehensive analysis of the longitudinal HDAg-specific CD8+ T-cell response. (A) Exemplary CD8+ T-cell response to an overlapping HDAg peptide and the corresponding pre-described optimal epitope. After peptide-specific culture, intracellular cytokine staining (IFNγ, TNF, and IL-2) was performed after restimulation without an addition of peptide (DMSO negative control), after restimulation with PMA and ionomycin (positive control), or after restimulation with OLP 48 (amino acid indicated) or the pre-described optimal HLA-B*51-restricted epitope $HDAg_{192–200}$ (amino acid sequence indicated) contained within OLP 48. IFNγ staining is displayed. (B) Positive responses for OLPs and optimal epitope peptides at baseline, FU1 and FU2. Positive responses are displayed in blue (epitopes with conserved amino acid sequence in the respective patient) or red (epitopes with mutated amino acid sequence in the respective patient). For the optimal epitope

pre-described CD8+ T-cell epitope, except for one patient who targeted two pre-described CD8+ T-cell epitopes at FU2. As expected, CD8+ T-cell responses identified by OLPs and pre-described CD8+ T-cell epitope peptides partially overlapped (marked by an asterisk in Fig. 2B). Combining results or overlapping peptides and optimal epitope peptides, 10/24 (41.7%), 10/24 (41.7%), and four of nine (44.4%) patients displayed a positive response at baseline, FU1, and FU2, respectively. When we longitudinally compared the strength of the positive CD8+ T-cell responses regarding production of IFNγ, tumor necrosis factor (TNF), and IL-2, we did not observe a significant evolution between baseline, FU1 and FU2 time points (Fig. 2C; a separate analysis of responses towards OLPs and pre-described epitopes is displayed in Fig. S2). In a subset of patients, we studied HDV-specific CD8+ T cells every 4–8 weeks of the first 48 weeks of therapy (n = 11), and at Week 2 of therapy (n = 4). Of note, we did not observe a transient increase of HDV-specific CD8+ T-cell responses in these patients. When we correlated the baseline presence as well as strength of combined positive responses (responses against OLPs + responses against pre-described epitopes) with the baseline HDV RNA levels, ALT levels, as well as HBsAg levels, we did not observe any significant correlation (Fig. 3A,B and Fig. S3A and B). In addition, the number or strength of baseline response was not associated with virological or biochemical response to BLV treatment (Fig. 3C,D, baseline data points; a separate analysis of responses towards OLPs and pre-described epitopes is displayed in Fig. S3C and D). Furthermore, longitudinal evolution of HDV-specific CD8+ T-cell responses did not differ between patients with or without virological or biochemical response (Fig. 3C,D and Fig. S3C and D). The six patients who had a biochemical response without a virological response displayed similar results (three with one CD8+ T-cell response at baseline and FU1, three without a response at both time points), indicating that this dissociation in treatment response is also not related to the HDV-specific CD8+ T-cell response.

Viral sequence mutations affect the large majority of HDV-specific CD8+ T-cell responses

We and others have described mutational viral escape from CD8+ T-cell responses in chronic HDV infection on both, the individual as well as population level.[7,8,10] We thus wondered how many of the detected CD8+ T-cell responses targeted mutated CD8+ T-cell epitopes. Indeed, the large majority of CD8+ T-cell responses detected in our patient cohort targeted epitopes with a non-prototype sequence: 10/15 (66.7%) responses at baseline, eight of 12 (66.7%) responses at FU1, and three of four (75%) responses at FU2 (Fig. 2B, responses to epitopes with non-prototype sequence displayed in red; Table S4). Of note, for some but not all viral variants viral escape has been demonstrated by functional assays previously.[7,8] These data thus suggest that the majority of CD8+ T-cell responses were affected by viral escape. Strikingly, the only CD8+ T-cell response that targeted the prototype sequence in the majority of patients (three of four patients, 75%) and that was maintained throughout treatment was directed against the HLA-B*35-restricted CD8+ T-cell epitope L-HDAg$_{192–200/194–202}$ (Fig. 2B).

Partial phenotypic amelioration of HLA-B*35 restricted HDV-specific CD8+ T cells targeting the wild-type epitope

In chronic HCV infection, sustained virological response to direct-acting antiviral therapy results in partial phenotypical restoration of HCV-specific CD8+ T cells with a shift from predominantly terminally exhausted to memory-like CD8+ T cells.[11–13] This effect is limited to CD8+ T cells targeting wild-type epitopes, because CD8+ T cells targeting escaped epitopes display a memory-like phenotype already before antiviral therapy, because of a lack of antigen triggering by the mutated epitope. We hypothesized that in patients with virological response to BLV therapy a similar amelioration of virus-specific CD8+ T cells may occur. We focused on the HLA-B*35 restricted CD8+ T-cell epitope L-HDAg$_{194–202}$, because this was the only fine-mapped HDV-specific CD8+ T-cell epitope with predominance of the wild-type epitope that was longitudinally detectable during BLV treatment in our patient cohort. Importantly, we were able to detect L-HDAg$_{194–202}$-specific CD8+ T cells *ex vivo* in two HLA-B*35:01+ patients: Patient P8 with a wild-type epitope sequence, and patient P12 with a variant epitope sequence. We performed a multiparameter peptide/HLA class I tetramer staining for a comprehensive panel of activation, exhaustion, and memory markers longitudinally over 48 weeks of BLV treatment (Fig. 4A, exemplary plots for tetramer-positive cells as well as baseline characteristics and on-treatment kinetics of ALT and HDV RNA levels of the two patients). T-distributed Stochastic Neighbor Embedding (t-SNE) analysis revealed that the HDV-specific CD8+ T cells from patient P8 targeting the wild-type epitope underwent phenotypical evolution from baseline to week 48 of BLV treatment (Fig. 4B, data of patient P8 displayed in blue color). Of note, the phenotypical evolution continuously progressed throughout the 48 weeks of BLV treatment (Fig. S4A depicts the t-SNE analysis for all time points analyzed). On a single marker level, HDV-specific CD8+ T cells expressed high levels of the activation markers CD38 and CD39 as well as the exhaustion-associated marker PD-1 at baseline, but a reduced expression of these markers and an increasing level of the memory and survival markers CD127, BCL2, and TCF1 during BLV treatment (Fig. 4C and Fig. S4B). Indeed, HDV-specific CD8+ T cells shifted from a terminally exhausted (PD1+CD127−) to a memory-like phenotype (PD1+CD127+) during BLV treatment (Fig. 4D). Of note, HDV-specific CD8+ T cells in patient P12 targeting a variant epitope sequence did not phenotypically evolve during BLV therapy, but displayed a memory-like phenotype already at baseline, in line with the assumption that the CD8+ T cells did not experience antigen triggering by the variant epitope (Fig. 4B, data of patient P12 displayed in red color). For the activation marker CD38, differences between HDV-specific CD8+ T cells targeting a

peptides, only peptides corresponding to the HLA class I alleles of the respective patient were tested; peptides that tested negative are displayed in grey. (C) Longitudinal strength of HDAg-specific CD8+ T-cell responses producing IFNγ, TNF, and IL-2 are displayed. Statistics: (C) Wilcoxon matched-pairs signed rank test. FU1, follow-up 1; FU2, follow-up 2; IFNγ, interferon-gamma; OLP, overlapping peptide; TNF, tumor necrosis factor.

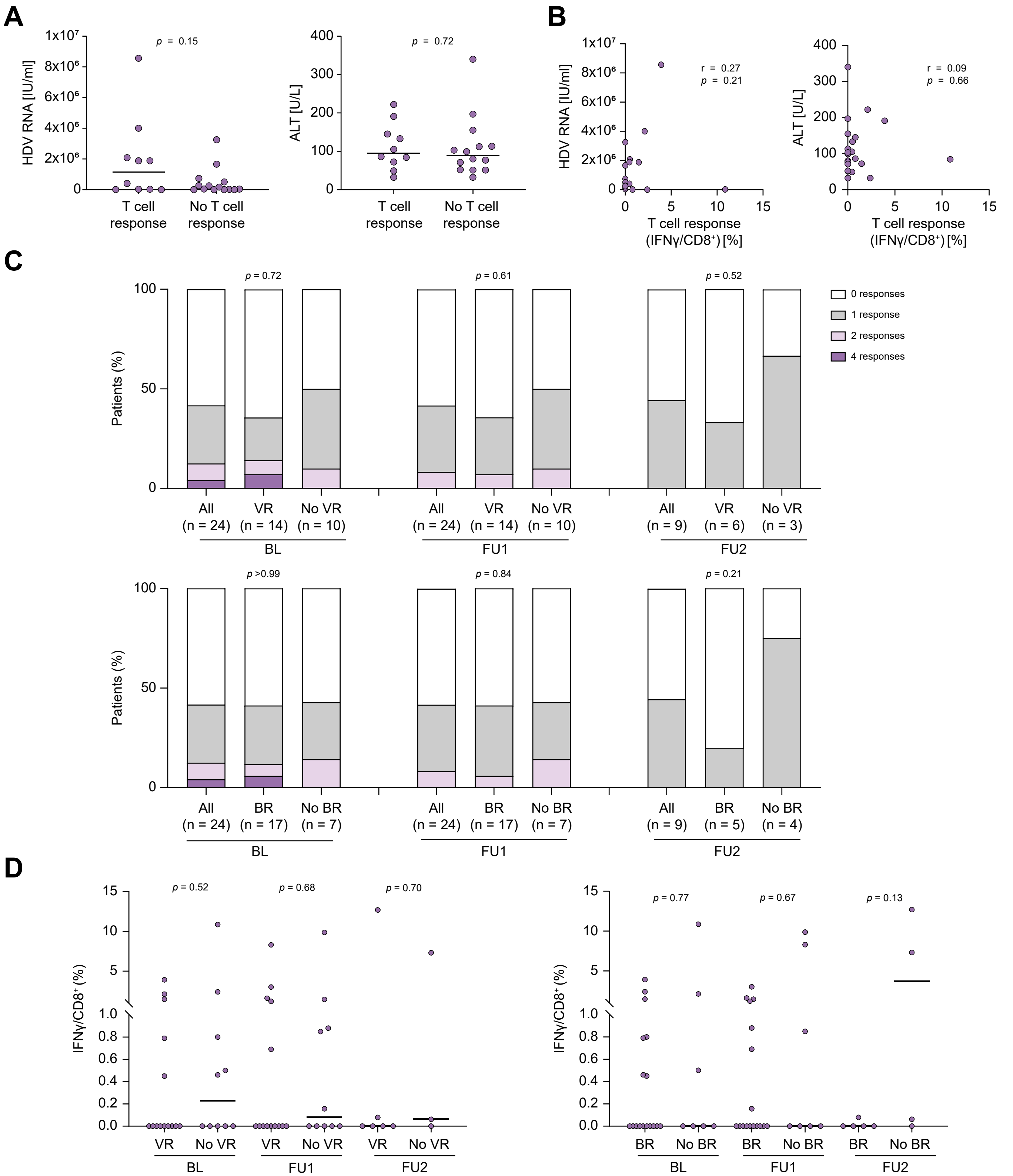

Fig. 3. Correlation of the HDAg-specific CD8+ T-cell response with baseline virological and biochemical data and treatment response. (A) Presence or absence of HDAg-specific CD8+ T-cell responses was correlated with HDV RNA and ALT levels. (B) Strength of HDAg-specific CD8+ T-cell responses was correlated with HDV RNA and ALT levels. (C,D) The longitudinal number (C) and strength (D) of HDAg-specific CD8+ T-cell responses is displayed for patients with or without virological response (VR) and with or without biochemical response (BR), respectively. For all graphs, CD8+ T-cell responses detected with OLPs or optimal epitope peptides were combined, counting responses against an OLP and an optimal epitope covered by this OLP only once. Statistics: (A,C,D): Mann–Whitney test; (B): non-parametric Spearman correlation. ALT, alanine aminotransferase; FU1, follow-up 1; FU2, follow-up 2; IFNγ, interferon-gamma; OLPs, overlapping peptides.

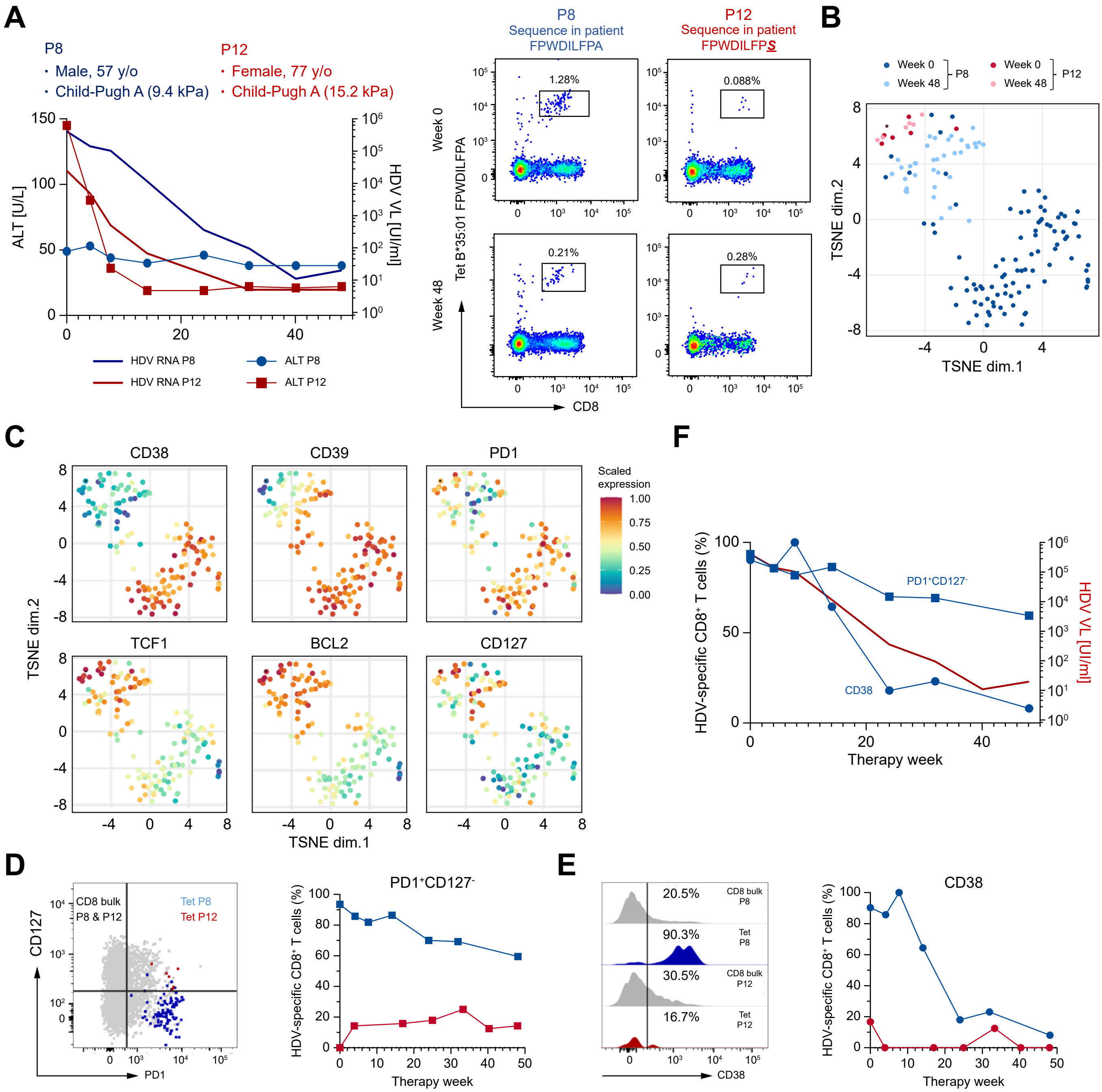

Fig. 4. BLV therapy ameliorates HDV-specific CD8+ T cells targeting epitopes with prototype viral sequence only. (A) Clinical data and tetramer staining of HLA-B*35:01-restricted $HDAg_{192–200}$-specific CD8+ T cells in patient P8 (prototype viral sequence) and patient P12 (mutated epitope sequence) at baseline and treatment Week 48, respectively. (B) t-SNE analysis of $HDAg_{192–200}$-specific CD8+ T cells from the two patients (P8 and P12) and the two time points (baseline and Week 48). (C) Expression level of the markers CD38, CD39, PD1, TCF1, BCL2, and CD127 blotted and the t-SNE distribution displayed in (B). (D) Longitudinal percentage of PD1+CD127– $HDAg_{192–200}$-specific CD8+ T cells in patients P8 and P12. The blot displays the expression of CD127 and PD1 at baseline. (E) Longitudinal percentage of CD38+ $HDAg_{192–200}$-specific CD8+ T cells in patients P8 and P12. The blot displays the expression of CD38 at baseline. (F) Longitudinal correlation of HDV RNA level, CD38 expression on $HDAg_{192–200}$-specific CD8+ T cells, and percentage of PD1+CD127– $HDAg_{192–200}$-specific CD8+ T cells in patient P8. ALT, alanine aminotransferase; t-SNE, T-distributed Stochastic Neighbor Embedding.

conserved epitope (patient P8) *vs.* a mutated epitope (patient P12) became most evident (Fig. 4E). For CD39, TCF1 and BCL2, differences between T cells targeting the conserved *vs.* mutated epitope were also obvious, but the temporal evolution was less marked compared with PD1; additional markers such as KLRG1, EOMAS, Tbet, TIGIT, and TOX did not substantially contribute to differential phenotypes or temporal evolution (Fig. S4C). Of note, the expression of activation, memory, and survival markers did not evolve differentially on memory-like (PD1+CD127+; Fig. S4D) *vs.* terminally exhausted

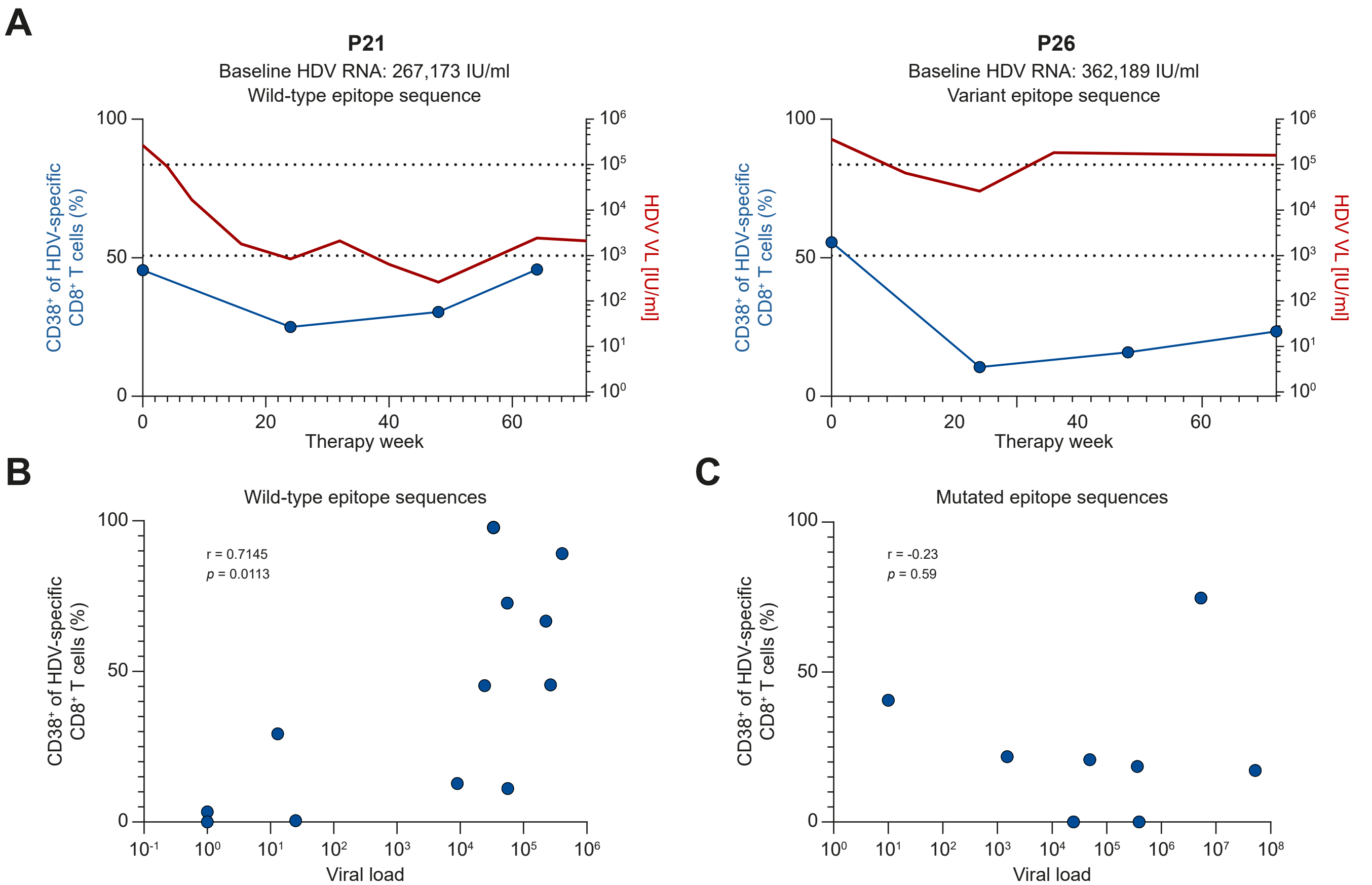

Fig. 5. CD38 expression by HDAg-specific CD8+ T cells mirrors the HDV RNA course in patients treated with BLV with viral rebound and correlates with HDV RNA in untreated patients. (A) Longitudinal HDV RNA levels and CD38 expression by HDAg-specific CD8+ T cells in BLV-treated patients P21 and P26. (B) Correlation of HDV RNA levels and percentage of CD38+ HDAg-specific CD8+ T cells in 11 BLV-naïve patients with chronic HDV infection displaying consensus epitope sequence (baseline data from patients P8, P21, P27, and P28 from the cohort of patients treated with BLV and patients P29–P34, P39, and P40 without BLV treatment; compare Tables S1 and S4). (C) Correlation of HDV RNA levels and percentage of CD38+ HDAg-specific CD8+ T cells in 8 BLV-naïve patients with chronic HDV infection displaying variant epitope sequence (Tables S1 and S4: baseline data from patients P12, P25, and P26 from the cohort of patients treated with BLV; patients P31 and P35–P38 without BLV treatment). Statistics: (B,C): non-parametric Spearman correlation. BLV, bulevirtide.

(PD1+CD127−; Fig. S4E) HDV-specific CD8+ T cells. Importantly, the global CD8+ T-cell population in the two patients did not show a phenotypic evolution during BLV therapy, further supporting that the phenotypic changes are indeed limited to HDV-specific CD8+ T cells that are triggered by antigen (Fig. S4F). When we compared the temporal decline of HDV RNA and the decline of the activation maker CD38 on HDV-specific CD8+ T cells as well as the frequency of terminally exhausted PD1+CD127− HDV-specific CD8+ T cells in patient P8 (conserved epitope), we observed that CD38 expression declined synchronically with HDV RNA levels, while the decline of terminally exhausted HDV-specific CD8+ T cells was delayed (Fig. 4F). This observation suggests that the decline of HDV viremia reduces activation of HDV-specific CD8+ T cells, leading to a delayed shift from predominantly terminally exhausted to maintained memory-like HDV-specific CD8+ T cells. It thus argues against a causal role of HDV-specific CD8+ T cells in HDV control during BLV treatment.

We extended the HDV-specific CD8+ T cell phenotype analyses in patients P21 and P26 who displayed an initial HDV RNA decline, followed by a partial viral rebound on treatment (Fig. 5A). HDV-specific CD8 T cells of these patients displayed similar kinetics of CD38 expression, with an initial decline followed by a rebound. Effects were more obvious for patient P21 targeting a wild-type epitope and more moderate in patient P26 targeting an epitope with viral variant sequence treatment (Fig. 5A). These data further support the concept that the partial amelioration is mainly driven by evolving HDV RNA load and not by the BLV treatment *per se*. This concept is further supported by a clear correlation between the activation marker CD38 on HDV-specific CD8+ T cells targeting conserved HDV epitopes and HDV RNA levels in BLV-naïve patients (Fig. 5B; these data include baseline data from patients P8, P27, and P28 from the cohort of BLV-treated patients and patients P29–P34, P39, and P40 without BLV treatment; for patient and epitope data compare Table S1 and Table S4, respectively). Of note, the same association was not found for HDV-specific CD8+ T cells targeting variant HDV epitopes in BLV-naïve patients (Fig. 5C; baseline data from patients P12, P25, and P26 from the cohort of BLV-treated patients and patients P31 and P35–P38 without BLV treatment; for patient and epitope data compare Tables S1 and S4, respectively).

Discussion

To our knowledge, this is the first study investigating T-cell responses in patients with CHD patients with cirrhosis treated with BLV monotherapy. In this analysis conducted on 28 patients, we found baseline detectable HDV-specific CD8+ T-cell response in 42%, but no substantial increase during BLV treatment for 40–120 weeks. Moreover, the large majority of HDV-specific CD8+ T-cell responses targeted viral epitopes with sequence variations consistent with viral escape mutations. This observation suggests that antiviral immunity is limited in its ability to support sustained virological response, even in the long-term. Of note, cure of HCV infection with direct-acting antivirals (DAA) leads to amelioration of HCV-specific CD8+ T cells, with a decline of terminally exhausted HCV-specific CD8+ T cells and an increase of memory-like HCV-specific CD8+ T cells, associated with an increase of pro-survival transcription factors and enhanced functional capacities.[11,12] These partially restored HCV-specific CD8+ T cells are thought to contribute to sustained virological response after DAA treatment of chronic HCV infection.

In contrast to HCV-specific CD8+ T cells, little is known about the fate of HDV-specific CD8+ T cells during antiviral therapy of CHD with either pegIFNα or BLV. Indeed, virological response to pegIFNα treatment was shown to be associated with sustained production of cytokines most likely by HDV-specific CD8+ T cells, whereas patients failing treatment displayed decreasing cytokine production during pegIFNα treatment.[14] Further analyses, including phenotypical analyses of HDV-specific CD8+ T cells was, however, not performed in this study. For BLV treatment, a very recent study demonstrated a shift in NK cell phenotype in patients with biochemical response *vs*. those without biochemical response.[15] However, HDV-specific CD8+ T cells were so far not studied during BLV treatment. We thus hypothesized that a partial restoration of HDV-specific CD8+ T cells during BLV treatment may contribute to viral suppression during treatment and prevention of HDV relapse off treatment.

Surprisingly, when we analyzed our cohort of patients with chronic HDV infection and progressed liver disease undergoing BLV treatment, we observed rather a decrease then an increase of HDV-specific CD8+ T cells. We used a sensitive method, including peptide-specific expansion of HDV-specific CD8+ T cells followed by intracellular cytokine staining (IFNγ, TNF, and IL-2) and used both, overlapping peptides spanning the complete L-HDAg as well as a set of five previously described fine-mapped HDV-specific CD8+ T-cell epitope peptides restricted by seven HLA-class I alleles. While this experimental set-up is very sensitive to detect antiviral CD8+ T cells capable to produce antiviral cytokines, it cannot detect terminally exhausted virus-specific CD8+ T cells that are severely compromised in their capability to expand and to produce antiviral cytokines. Thus, the fact that we did not observe an increased frequency of HDV-specific CD8+ T cells during BLV treatment even stronger argues against a general increase or functional restoration of HDV-specific CD8+ T cells during BLV treatment in our cohort.

When we correlated our data on targeted HDV-specific CD8+ T-cell epitopes with the autologous viral sequences obtained from the respective patients, we found that at baseline, two-thirds of HDV-specific CD8+ T-cell responses targeted epitope sequences with amino acid variations compared with the prototype sequence, suggesting viral escape.[7] Strikingly, during BLV treatment, the HDV-specific CD8+ T-cell response seemed to narrow and focus on an HLA-B*35-restricted CD8+ T-cell epitope that displayed a conserved viral sequence in the majority of patients. Intriguingly, HLA-B*35:01 was the only HLA class I allele that was enriched in patients with virological response in our cohort. It is important to note, however, that this association was only borderline significant ($p = 0.055$) and was only present for HLA-B*35:01 but not B*35:02 or B*35:03. Thus, definitively larger patient cohorts will be needed to confirm a beneficial role of HLA-B*35:01 during BLV treatment.

The positive detection of HLA-B*35-restricted HDV-specific CD8+ T cells by peptide/HLA class I tetramer staining allowed us to perform a multiparameter phenotypical analysis of HDV-specific CD8+ T cells during BLV treatment. Of note, HDV-specific CD8+ T cells targeting the prototype sequence displayed a terminally exhausted phenotype at baseline, but shifted to a memory-like phenotype gradually during the first 48 weeks of BLV treatment. By contrast, HDV-specific CD8+ T cells targeting a variant epitope sequence in another HLA-B*35+ patient displayed a memory-like phenotype already at baseline that did not change during BLV treatment, consistent with the notion that these HDV-specific CD8+ T cell did not recognize their cognate antigen and are thus not driven into exhaustion by repetitive antigen triggering. When we correlated the evolution of the HDV-specific CD8+ T-cell phenotype and the HDV RNA level during the course of BLV treatment, we observed that the phenotypic changes followed the decrease of viral load, suggesting that the amelioration of the CD8+ T-cell phenotype is rather a consequence and not a cause of viral control and antigen reduction. The observed shift toward a memory-like phenotype thus reflects reduced antigenic stimulation rather than immune recovery, explaining that it does not translate into functional improvement or contribution to viral control. This concept was also supported by the correlation of the expression of the activation marker CD38 on HDV-specific CD8+ T cells and HDV RNA levels in patients not undergoing antiviral treatment, and the similar evolution of expression of other markers by memory-like (PD1+CD127+) *vs*. terminally exhausted (PD1+CD127−) subsets. Interestingly, our findings in HDV infection partially mirror previous findings in HCV infection.[11,12] In both infections, memory-like virus-specific CD8+ T cells are enriched compared with terminally exhausted T cells during antiviral therapy, resulting in an overall increased expression of survival markers such as TCF-1 and BCL2 on virus-specific CD8+ T cells. These changes were, however, much more pronounced in HCV infection, likely linked to sustained viral control in this infection, and rather decent in HDV, where HDV is rather controlled then completely eliminated. On the memory-like T cell subset, however, only the activation marker CD38 showed a substantial decline during antiviral treatment in both infections, indicating that rather a shift of T-cell subsets then a real reprogramming of virus-specific CD8+ T cells occurred.

Our data thus indicate that amelioration of HDV-specific CD8+ T cells during BLV treatment is possible in principle, however, most HDV-specific CD8+ T-cell epitopes are affected by viral escape. Thus, in the overall cohort, amelioration of

HDV-specific CD8+ T cells does not play a substantial role. These results partially explain that long-term BLV treatment is necessary to prevent viral relapse.

Of note, our study has some limitations. First, decline of HDV viremia is slow during BLV monotherapy, so amelioration of HDV-specific CD8+ T cells may require more time than 1–2 years. Second, all patients analyzed in our study had advanced liver disease, possibly limiting antiviral CD8+ T-cell immunity. However, we did not observe a clear difference between HDV-specific CD8+ T-cell responses in patients with cirrhosis *vs*. patients without cirrhosis when we analyzed an independent cohort of 60 patients with hepatitis delta, using the same approach (unpublished data). Third, our cohort only included patients with BLV monotherapy (on a nucleos(t)ide analogue backbone), but did not include antiviral treatment aiming at HBsAg reduction. As HDV contains both HDAg and HBsAg as possible targets of antiviral CD8+ T-cell immunity, it will be important to study HDAg- as well as HBsAg-specific CD8+ T cells in patients undergoing BLV/pegIFN combination therapy as well as in patients undergoing novel treatments such as monoclonal antibodies and siRNA. These treatments may restore HBsAg-specific CD8+ T cells and contribute to prevention of HDV relapse. Fourth, our findings are based on a rather small number of patients with detectable HDV-specific CD8+ T cell responses. Viral escape as a frequent limitation of HDV-specific CD8+ T-cell responses has been supported by studies from us and others.[7,8] The finding that BLV treatment allows amelioration of HDV-specific CD8+ T cell responses targeting wild-type epitopes clearly requires confirmation in future studies. Fifth, our study only analyzed peripheral HDV-specific CD8+ T cells, while these are likely enriched in the liver and may also differentially react to antiviral therapy. Sixth, we limited our study to the CD8+ T-cell compartment. In the OLP experiments, we also detected a small number of CD4+ T cell responses without a trend towards enhanced number or magnitude during BLV treatment, but this observation requires confirmation. In addition to these conceptual limitations, our study also has some methodical limitations. Although the combined use of overlapping peptides and optimal peptides of described HDV-specific CD8+ T-cell epitopes is the most comprehensive approach used to date, some HDV-specific CD8+ T-cell responses may have been missed because of the use of peptides based on the HDV genotype 1 consensus sequence and not patient-specific sequences. In addition, overlapping peptides with the length of 14 or 15 amino acids do not always stimulate CD8+ T cells that usually recognize 8- to 10-mer epitope peptides. In line with this limitation, a combined analyses of responses towards pre-described epitope peptides and OLPs did not differ from (and not substantially add to) the analyses performed with pre-described epitope peptides only. Further, because of the very low frequencies of HDV-specific CD8+ T cells, we could not perform functional assays *ex vivo*, thus we can only speculate on the functional implications of the phenotypic changes observed *ex vivo*.

In sum, HDV-specific CD8+ T cells can be ameliorated during BLV monotherapy, but this effect likely has little impact on antiviral control during BLV treatment or on prevention of HDV relapse off treatment, because viral escape is the dominant mechanism underlying the ineffective HDV-specific CD8+ T-cell response.

Affiliations

[1]*Department of Medicine II, Freiburg University Medical Center, Faculty of Medicine, University of Freiburg, Freiburg, Germany;* [2]*Division of Gastroenterology and Hepatology, Foundation IRCCS Ca' Granda Ospedale Maggiore Policlinico, Milan, Italy;* [3]*Faculty of Biology, University of Freiburg, Freiburg, Germany;* [4]*Department of Gastroenterology and Hepatology, University Hospital Cologne, Faculty of Medicine, University of Cologne, Cologne, Germany;* [5]*University of Paris-Cité, Hôpital Beaujon, Service d'hépatologie AP-HP & INSERM UMR1149, Clichy, France;* [6]*Division of Infection and Immunity, Cardiff University School of Medicine, University Hospital of Wales, Cardiff, UK;* [7]*Institute for Transfusion Medicine and Gene Therapy, Freiburg University Medical Center, Faculty of Medicine, University of Freiburg, Freiburg, Germany;* [8]*Systems Immunity Research Institute, Cardiff University School of Medicine, University Hospital of Wales, Cardiff, UK;* [9]*Signalling Research Centres BIOSS and CIBSS, University of Freiburg, Freiburg, Germany;* [10]*CRC "A. M. and A. Migliavacca" Center for Liver Disease, Department of Pathophysiology and Transplantation, University of Milan, Milan, Italy;* [11]*D-SOLVE consortium, an EU Horizon Europe funded project (No 101057917), Hannover, Germany;* [12]*German Centre for Infection Research (DZIF), Site Bonn-Cologne, Cologne, Germany;* [13]*Center for Molecular Medicine Cologne (CMMC), University of Cologne, Cologne, Germany*

Abbreviations

ALT, alanine aminotransferase; BLV, bulevirtide; CHD, chronic hepatitis delta; DAA, direct-acting antivirals; FU1, follow-up 1; FU2, follow-up 2; IFNγ, interferon-gamma; OLPs, overlapping peptides; PBMCs, peripheral blood mononuclear cells; pegIFNα, pegylated interferon-alpha; TNF, tumor necrosis factor; t-SNE, T-distributed Stochastic Neighbor Embedding.

Financial support

This project was supported by grants from the Deutsche Forschungsgemeinschaft (DFG, German Research Foundation; TRR-179, project number 272983813, to TB, BB, HL, MH, RT, and CNH; SFB-1160, project number 256073931, to BB, MH, RT, and CNH) and the Deutsches Zentrum für Infektionsforschung (DZIF, German Center for Infection Research; project number TTU 05.822, to CNH). MH was supported by the DFG Heisenberg program (project no. HO 5836/2-1). DAP was supported by the PolyBio Research Foundation (Balvi B43). The authors thank the D-SOLVE consortium, a European Union funded project (No 101057917).

Conflicts of interest

ED: on advisory board for Gilead Sciences, IPSEN, and Roche; speaking and teaching for Gilead Sciences and IPSEN; travel grant from Gilead Sciences; and research grant from Gilead Sciences. TA: consultant, expert, and speaker for Bluejay, Gilead Sciences, GSK, Janssen, MYR Pharmaceuticals, and VIR Biotechnology. TB: advisor and speaker bureau for Gilead Sciences and Falk Foundation. BB: advisor for Roche and speaker bureau for Falk Foundation, BMS, AstraZeneca, and Roche. RT: speaker bureau for Bayer Vital and Falk Foundation and advisor for AbbVie, Gilead Sciences, OPASCA, Topas Therapeutics, and Roche. PL: advisor and speaker bureau for Roche Pharma/Diagnostics, Gilead Sciences, GSK, AbbVie, Janssen, MYR, Eiger, Antios, Aligos, VIR, Grifols, Altona, Roboscreen, and Bluejay. CN–H: speaker bureau for AbbVie, Gilead Sciences, MSD, Pfizer, and Falk Foundation. The remaining authors declare no conflicts of interest that pertain to this work.

Please refer to the accompanying ICMJE disclosure forms for further details.

Authors' contributions

Conceptualization: PL, CNH. Recruitment of patients: ED, MB, RS, MPA, FF, SN, TA, TB, BB, HL. Four-digit HLA-typing: FE. Designs and synthesis of peptide/

HLA class I tetramers: SLL, DAP. Performance and analysis of viral sequencing and immunological data: VO, MM, KH, ESA, ÖS, FW. Interpretation of data: VO, MM, MH, RT, PL, CNH. Drafting of the manuscript: VO, MM, HL, MH, RT, PL, CNH. Reviewing and editing of the manuscript: all authors. Approved the submitted version: all authors.

Data availability

No data are deposited on public databases. All requests for raw and analyzed data and materials will be reviewed by the corresponding author to verify if the request is subject to any confidentiality obligations. Patient-related data not included in the paper were generated as part of clinical examination and may be subject to patient confidentiality. Any data and materials that can be shared will be released via a material transfer agreement.

Supplementary data

Supplementary data to this article can be found online at https://doi.org/10.1016/j.jhepr.2026.101970.

References

Author names in bold designate shared co-first authorship

[1] Asselah T, Rizzetto M. Hepatitis D virus infection. N Engl J Med 2023;389:58–70.
[2] Degasperi E, Anolli MP, Jachs M, et al. Real-world effectiveness and safety of bulevirtide monotherapy for up to 96 weeks in patients with HDV-related cirrhosis. J Hepatol 2025;82:1012–1022.
[3] Wedemeyer H, Aleman S, Brunetto M, et al. Bulevirtide monotherapy in patients with chronic HDV: efficacy and safety results through week 96 from a phase III randomized trial. J Hepatol 2024;81:621–629.
[4] Wedemeyer H, Aleman S, Brunetto MR, et al. A phase 3, randomized trial of bulevirtide in chronic hepatitis D. N Engl J Med 2023;389:22–32.
[5] **Wedemeyer H**, **Aleman S**, Blank A, et al. 144 Weeks of bulevirtide monotherapy for chronic hepatitis D: final and posttreatment results from a Phase 3 randomized trial. J Hepatol 2026;85:493–503.
[6] European Association for the Study of the Liver. EASL clinical practice guidelines on hepatitis delta virus. J Hepatol 2023;79:433–460.
[7] **Karimzadeh H**, **Kiraithe MM**, **Oberhardt V**, et al. Mutations in hepatitis D virus allow it to escape detection by CD8+ T cells and evolve at the population level. Gastroenterology 2019;156:1820–1833.
[8] Kefalakes H, Koh C, Sidney J, et al. Hepatitis D virus-specific CD8+ T cells have a memory-like phenotype associated with viral immune escape in patients with chronic hepatitis D virus infection. Gastroenterology 2019;156:1805. 19.e1809.
[9] Landahl J, Bockmann JH, Scheurich C, et al. Detection of a broad range of low-level major histocompatibility complex class II-restricted, hepatitis delta virus (HDV)-specific T-cell responses regardless of clinical status. J Infect Dis 2019;219:568–577.
[10] Karimzadeh H, Kiraithe MM, Kosinska AD, et al. Amino acid substitutions within HLA-B*27-restricted T cell epitopes prevent recognition by hepatitis delta virus-specific CD8+ T cells. J Virol 2018;92:e01891–17.
[11] **Hensel N**, **Gu Z**, **Sagar**, et al. Memory-like HCV-specific CD8+ T cells retain a molecular scar after cure of chronic HCV infection. Nat Immunol 2021;22:229–239.
[12] Tonnerre P, Wolski D, Subudhi S, et al. Differentiation of exhausted CD8+ T cells after termination of chronic antigen stimulation stops short of achieving functional T cell memory. Nat Immunol 2021;22:1030–1041.
[13] Wieland D, Kemming J, Schuch A, et al. TCF1+ hepatitis C virus-specific CD8+ T cells are maintained after cessation of chronic antigen stimulation. Nat Commun 2017;8:15050.
[14] Grabowski J, Yurdaydin C, Zachou K, et al. Hepatitis D virus-specific cytokine responses in patients with chronic hepatitis delta before and during interferon alfa-treatment. Liver Int 2011;31:1395–1405.
[15] Chen PC, Deterding K, Engelskircher SA, et al. TIGIT expression on natural killer cell subsets is an early indicator of alleviating liver inflammation following bulevirtide treatment in chronic hepatitis D. Hepatology 2025;82:1565–1581.

Keywords: Hepatitis D virus; Hepatitis delta; Virus-specific CD8+ T cell; Viral escape; Bulevirtide.
Received 5 January 2026; received in revised form 26 June 2026; accepted 14 July 2026; Available online 24 July 2026

Journal of Hepatology, Volume *8*

Supplemental information

Fate of hepatitis D virus-specific CD8+ T cells during bulevirtide mono-therapy in patients with chronic hepatitis delta

Valerie Oberhardt, Elisabetta Degasperi, Michelle Maas, Marta Borghi, Kathrin Heim, Roberta Soffredini, Maria Paola Anolli, Floriana Facchetti, Stéphanie Narguet, Tarik Asselah, Elahe Salimi Alizei, Özlem Sogukpinar, Frances Winkler, Sian Llewellyn-Lacey, Florian Emmerich, David A. Price, Tobias Boettler, Bertram Bengsch, Hendrik Luxenburger, Maike Hofmann, Robert Thimme, Pietro Lampertico, and Christoph Neumann-Haefelin

Fate of hepatitis D virus-specific CD8+ T cells during bulevirtide monotherapy in patients with chronic hepatitis delta

Valerie Oberhardt, Elisabetta Degasperi, Michelle Maas*, Marta Borghi, Kathrin Heim, Roberta Soffredini, Maria Paola Anolli, Floriana Facchetti, Stéphanie Narguet, Tarik Asselah, Elahe Salimi Alizei, Özlem Sogukpinar, Frances Winkler, Sian Llewellyn-Lacey, Florian Emmerich, David A. Price, Tobias Boettler, Bertram Bengsch, Hendrik Luxenburger, Maike Hofmann, Robert Thimme, Pietro Lampertico, Christoph Neumann-Haefelin

Table of contents

Supplementary methods…………………………………………………………………………2
Table S1…………………………………………………………………………………………...6
Table S2…………………………………………………………………………………………...7
Table S3…………………………………………………………………………………………...9
Table S4………………………………………………………………………………………….10
Fig. S1…………………………………………………………………………………………...11
Fig. S2…………………………………………………………………………………………12
Fig. S3…………………………………………………………………………………………13
Fig. S4…………………………………………………………………………………………15
Fig. S5…………………………………………………………………………………………20

Supplementary methods

PBMC isolation

Venous blood samples (50 mL per draw) were collected in EDTA-anticoagulated tubes. Peripheral blood mononuclear cells (PBMCs) were isolated with lymphocyte separation medium by density gradient centrifugation (Pancoll separation medium, PAN Biotech GmbH; Aidenbach, Germany). PBMCs were stored at -80°C. Frozen PBMCs were thawed and resuspended in RPMI 1640 medium supplemented with 10% fetal calf serum, 1% penicillin/streptomycin, and 1.5% HEPES buffer 1 mol/L containing 50 U/mL benzonase (Sigma-Aldrich) (RPMI complete medium; all additives from ThermoScientific; Waltham, MA).

Peptides and tetramers for T-cell analysis

A total of 51 overlapping peptides (OLP) spanning the L-HDAg (consensus HDV genotype 1 sequence obtained from 104 patients [1]) were synthesized as 14- or 15-mers overlapping by 11 amino acids with an unmodified amine NH2 terminus and an amidated acid COOH terminus with standard Fmoc chemistry and a purity of >70% (Genaxxon Bioscience, Ulm, Germany; **Table S2**). Additionally, peptides for optimal epitopes (9-10-mers) were synthesized in the same manner (**Table S3**). Peptide/HLA-class I tetrameric complexes were generated as described previously [2]. For HLA-B*08:01 tetramers, peptide was loaded on HLA class I easYmers (immunAware) according to the manufacturer´s instructions. For tetramerization, the HDV peptide-loaded HLA class I monomers were subsequently incubated together with phycoerythrin (PE)- conjugated streptavidin (Agilent) following the manufacturer´s protocol.

In vitro expansion and intracellular IFNγ staining using overlapping peptides or pre-described optimal CD8+ T-cell epitopes

In vitro expansion with OLPs was performed as follows: 20% of the PBMCs were stimulated with a pool of all 51 OLPs (5 µM/peptide) for 1 h at 37 °C, washed and co-cultured with the remaining PBMCs in RPMI complete medium supplemented with 20 U/mL with recombinant interleukine-2 (IL-2) (StemCell Technologies) and anti-CD28 monoclonal antibody (0.5 µg/mL) (BD Bioscience). Alternatively, PBMCs were stimulated with optimal peptides (10 µM) for *in vitro* expansion. Cells were then fed every 3 days with RPMI complete medium containing 20 U/mL recombinant interleukin (IL-2). On day 10, intracellular IFNγ staining was performed with 12 smaller pools of OLPs (9 pools with 4 OLPs each and 3 pools with 5 OLPs

each). Therefore, cells were re-stimulated with OLP pools (25 µM/peptide for 1h; 5 µM/peptide final concentration), dimethyl sulfoxide as a negative control or phorbol 12-myristate 13-acetate and ionomycin as positive control. After 1h at 37°C, Brefeldin A (GolgiPlug, 0.5 µl/mL) and monensin (GolgiStop, 0.5 µl/mL) (both BD Bioscience) were added. After an additional incubation for 4 h, surface (anti-CD8-APC, anti-CD4-BV421; Viaprobe) and intracellular (anti-IFNγ-FITC) staining was performed. Stained cells were fixed in phosphate-buffered saline (PBS) containing 2% paraformaldehyde (Sigma-Aldrich) and were acquired via flow cytometry. Subsequently, on day 12-14 single OLPs of positive pools and HLA-matched optimal CD8+ T-cell epitopes were tested by intracellular cytokine staining. In particular, cells were re-stimulated with individual OLP (25 µM for 1h; 5 µM final concentration) or optimal CD8+ T-cell epitopes (75 µM for 1h; 15 µM final concentration), dimethyl sulfoxide as a negative control or phorbol 12-myristate 13-acetate and ionomycin as positive control. After 1h at 37°C, Brefeldin A (GolgiPlug, 0.5 µl/mL) and monensin (GolgiStop, 0.5 µl/mL) (both BD Bioscience) were added. After an additional incubation for 4 h, surface (anti-CD8-APC, anti-CD4-BV421; Viaprobe) and intracellular (IFNγ-FITC, TNF-PE-Cy7, anti-IL-2-PerCP-Cy5.5) staining was performed. Data were acquired with and BD FACSCanto II flow cytometer (BD Biosciences) and analyzed with FlowJo software, version 10.6.2 (FlowJo, Ashland, OR).

Analysis of HDV sequences and HLA class I-associated viral polymorphism

Total RNA was extracted from serum or plasma samples using the QiAmp Viral RNA Kit (Qiagen, Hilden, Germany) and with SuperScript III First strand synthesis Kit for RT PCR (Invitrogen, MA, USA) and HDV-specific primer 771R (5´-CGGTCCCCTCGGAATGTTG-3´) reverse transcribed into cDNA. By using GoTaq®G2 Flexi DNA Polymerase (Promega, WI, USA), the L-HDAg-encoding region was amplified from cDNA in a 2-step nested polymerase chain reaction (PCR) incoporating the HDV-specific primers 891F (5´-AGGTCGGACCGCGRGGAGGT-3`), 339R (5´-GCTGAAGGGGTCCTCTGGAGGTG-3`), 912F (5´-GAGATGCCATGCCGACCCGAAGAG-3´) and 1674R (5´-AGAAAAGAGTAAGAGYACTGAGG-3´). According thermal profile was used for the first PCR: (i) 94 °C for 3 minutes; (ii) 94 °C for 1 minute, (iii) 94 °C for 30 seconds, 70 °C-1dT 1 °C (70°-58 °C) for 45 seconds, 72 °C for 90 seconds (12 cycles); (iiii) 94 °C for 30 seconds, 58 °C for 45 seconds, 72 °C for 90 seconds (23 cycles) and (iiiii) 72 °C for 7 minutes. The second PCR thermal profile contains the following steps: (i) 94 °C for 3 minutes; (ii) 97 °C for 1minute, (iii) 94 °C for 30 seconds, 70 °C-1dT 1 °C (70°-55 °C) 45 seconds, 72 °C for 90 seconds (15 cycles); (iiii) 94 °C for 30 seconds, 55 °C for 45 seconds, 72 °C for 90 seconds (20 cycles) and (iiiii) 72 °C for 7 minutes.

PCR products were purified with the QuickStep™2 PCR purification kit (EdgeBio, CA, USA) and sequenced on an ABI 3730xl DNA Analyzer (Eurofins Genomics Germany GmbH; Ebersberg, Germany) with the internal primers 912F and 1674R. Sequence homology analyses were performed in Genious Prime v.11.0.5. The reference genome of human HDV (M21012.1) was downloaded from the National Center for Biotechnology Information data base (https://www.ncbi.nlm.nih.gov/nuccore/M21012.1). HDV epitopes were then mapped to the corresponding protein alignment.

Magnetic bead-based enrichment of HDV-specific CD8+ T cells

Tetramer-based enrichment was performed as described by Alanio *et al.* [3]. Briefly, $1 \times 10^7 - 2 \times 10^7$ PBMCs were labelled with PE-coupled peptide-loaded HLA class I tetramers for 30 min. Next, the enrichment was performed using anti-PE beads with MACS technology (Miltenyi Biotec) according to the manufacturer´s instructions. Subsequently, analysis of the enriched HDV-specific CD8+ T cells was conducted by multiparametric flow cytometry and frequencies of HDV-specific CD8+ T cells were calculated as follows: Absolute number of HDV-specific CD8+ T cells (enriched sample) divided by the absolute number of CD8+ T cells (pre-enriched sample) x 200. Only samples including > 5 HDV-specific CD8+ T cells were used for further subset gating and phenotypic analyses (detection limit of 5×10^{-6}).

Multiparametric flow cytometry for T-cell analysis

The following antibodies were used for multiparametric flow cytometry: anti-CD8-BUV395 (RPA-T8, 1:400), anti-CD45RA-BUV496 (HI100, 1:800), anti-CD127-BUV737 (HIL-7R-M21, 1:50), anti-BCL-2-BV421 (100; 1:100), anti-TIGIT-BV510 (A15153G; 1:8.3), anti-PD-1-BV605 (EH12.1; 1:25), anti-CD39-BV650 (TU66, 1:1.5), anti-KLRG1-BV711 (13F12F2, 1:50), anti-CCR7-BV786 (G043H7, 1:50), anti-TCF1-AlexaFluor488 (C63D9, 1:100) (Cell Signaling), anti-Eomes-PE-Cy5.5 (104; 1:800), anti-CD244-PE-CF594 (C1.7, 1:200), anti-Tbet-PE-Cy7 (4B10, 1:200), anti-TOX-eFluor660 (TRX19, 1:100) (Thermo Fisher), anti-CD38-AlexaFluor700 (HIT2, 1:400), anti-CD19-APC-eF780 (HIB19, 1:400), anti-CD14-APC-eF780 (61D3, 1:400), anti-IFNγ-FITC (25723.11, 1:50), anti-CD4 (RPA-T4, 1:500), anti-TNF-α-PE-Cy7 (MAb11, 1:400) (BD Bioscience), anti-IL-2-PerCP-Cy5.5 (MQ1-17H12, 1:100). For live/dead discrimination a fixable Viability Dye (APC-eFluor780 1:200) was used. All antibodies were purchased from BD Biosciences (Germany), Thermo Scientific and Cell Signaling Technology (Danvers, MA). FoxP3/Transcription Factor Staining Buffer Set (Thermo Fisher) and Fixation/Permeabilization Solution Kit

(BD Biosciences) were used according to the manufacturer's protocol to stain for intranuclear and cytoplasmic molecules, respectively. After fixation of cells in 2 % para- formaldehyde (PFA, Sigma), analyses were performed on a FACSCanto II or LSRFortessa with FACSDiva software version 10.6.2 (BD). Data were analysed with FlowJo 10.6.2 (Treestar). Gating strategy is displayed in **Fig. S5.**

Dimensional reduction of multiparametric flow cytometry data

Tetramer-positive CD8+ T cells were defined using FlowJo 10. Only samples with ≥ 5 HDV-specific CD8+ T cells were included in the dimensionality reduction analysis. Dimensionality reduction of flow cytometry data was performed with R version 4.5.0 using the Bioconductor CATALYST package (version 1.32.0). The analyses were performed on gated virus-specific CD8+ T cells including the markers CD38, CD39, PD1, TCF-1, BCL-2, CD127, CD45RA, CCR7, EOMES, TIGIT, KLRG1, 2B4, T-BET and TOX. Downsampling of cells to 100 or 200 cells (t-SNE or diffusion maps) was performed before dimensionality reduction to facilitate the visualization of different samples.

Statistics

Statistical analysis was performed with GraphPad Prism 10 (GraphPad Prism Software). Statistical significance was assessed by Mann-Whitney test, nonparametric Spearman correlation, or Wilcoxon matched-pairs signed rank test as indicated. Analyses were performed in independent experiments.

References for Supplementary methods

1 Karimzadeh H, Kiraithe MM, Kosinska AD, Glaser M, Fiedler M, Oberhardt V*, et al.* Amino acid substitutions within HLA-B*27-restricted T cell epitopes prevent recognition by hepatitis delta virus-specific CD8+ T cells. J Virol 2018;**92**.
2 Price DA, Brenchley JM, Ruff LE, Betts MR, Hill BJ, Roederer M*, et al.* Avidity for antigen shapes clonal dominance in CD8+ T cell populations specific for persistent DNA viruses. J Exp Med 2005;**202**:1349-61.
3 Alanio C, Lemaitre F, Law HK, Hasan M, Albert ML. Enumeration of human antigen-specific naive CD8+ T cells reveals conserved precursor frequencies. Blood 2010;**115**:3718-25.

Table S1: Patients' characteristics

ID	Age	Sex	Country of birth	Liver elastography (kPa)	HDV RNA (log10)	ALT (U/L)	Child -Pugh Score	Oesoph. varices	Bilirubin (mg/dl)	Virological response	Bio-chemical response	HLA-A1	HLA-A2	HLA-B1	HLA-B2	Epitope repertoire analysis	T-cell pheno-typing
P1	62	M	caucasian	7.8	4.64	99	A5	yes	1.4	yes	yes	32:01	33:01	14:02	51:01	yes	
P2	40	F	caucasian	24.0	4.34	113	A6	yes	1.5	yes	no	02:01	03:01	35:03	51:01	yes	
P3	62	M	caucasian	39.8	6.22	80	A6	yes	1.4	no	no	24:02	32:01	39:01	50:01	yes	
P4	40	F	caucasian	14.6	3.31	51	A5	yes	0.5	yes	yes	23:01	24:02	44:03	51:01	yes	
P5	70	F	caucasian	16.5	4.58	155	A6	no	1	yes	yes	02:01	33:01	14:02	44:02	yes	
P6	29	M	caucasian	10.9	6.32	133	A5	yes	1	no	no	02:01	24:02	18:01	35:03	yes	
P7	49	M	caucasian	11.6	3.95	86	A5	yes	1.7	no	yes	11:01	24:02	35:02	35:03	yes	
P8	57	F	caucasian	9.4	5.61	49	A5	no	0.9	yes	yes	01:01	11:01	35:01	41:01	yes	yes
P9	34	F	caucasian	16.3	4.39	84	A5	yes	1.4	no	no	02:01	24:02	14:02	51:01	yes	
P10	54	M	caucasian	33.3	6.60	222	A6	yes	1	yes	no	01:01	03:01	51:01	57:01	yes	
P11	68	M	caucasian	15.5	6.27	105	A5	yes	1.5	no	yes	31:01	68:01	18:01	44:03	yes	
P12	77	M	caucasian	15.2	4.39	145	A5	no	0.5	yes	yes	03:01	11:01	35:01	35:03	yes	yes
P13	59	M	caucasian	57.8	5.87	112	A5	yes	1.4	yes	yes	31:01	32:01	18:01	39:06	yes	
P14	47	F	caucasian	17.4	3.74	32	B7	yes	1.8	no	yes	02:01	02:06	15:01	48:01	yes	
P15	37	F	caucasian	22.3	6.27	72	A5	no	0.8	yes	yes	02:01		18:31	44:03	yes	
P16	67	F	tunisian	54.5	6.93	191	A6	yes	0.8	yes	yes	23:01	30:02	50:01	50:02	yes	
P17	56	M	egyptian	41.2	5.70	340	A5	yes	1.2	yes	yes	02:05	68:01	50:01	57:02	yes	
P18	38	F	caucasian	21.2	6.51	197	B7-8	yes	2.9	yes	yes	31:01	68:01	40:01	55:01	yes	
P19	51	M	caucasian	32.8	2.76	52	A6	no	0.9	no	no	02:01	24:02	51:01		yes	
P20	36	F	caucasian	16.5	2.43	51	B8	yes	2.1	no	yes	01:01	03:01	14:01	44:02	yes	
P21	50	M	caucasian	33.2	5.43	77	C10	yes	2.43	yes	no	02:01		07:02	35:01	yes	yes
P22	60	F	caucasian	26.2	5.22	71	A6	yes	1.19	yes	yes	11:01	24:02	40:01	51:01	yes	
P23	44	M	caucasian	ND	2.90	32	A5	yes	0.55	no	yes	02:01	03:01	07:02	40:01	yes	
P24	47	F	caucasian	ND	5.39	103	B8	yes	1	no	yes	02:01		41:01	51:01	yes	
P25	40	M	caucasian	8.9	5.58	172	A5	yes	0.6	yes	no	02:01		35:01	44:02		yes
P26	58	F	caucasian	53.1	5.56	44	A5	yes	0.9	no	no	01:01	11:01	15:02	37:01		yes
P27	59	M	caucasian	62.1	5.35	71	B7	yes	1.9	yes	no	01:01	02:01	35:01	49:01		yes
P28	53	F	caucasian	9.6	4.75	30	A5	no	0.7	no	no	01:01	11:01	07:02	15:01		yes
P29	68	M	caucasian	12.3	1.11	34	A5	no	0.9	no BLV therapy		01:01	02:01	08:01	27:02		yes
P30	41	M	caucasian	8.1	4.52	89	no cirrhosis	no	0.5	no BLV therapy		01:01	33:03	08:01	52:01		yes
P31	43	M	caucasian	10.0	1.00	35	no cirrhosis	no	1.7	no BLV therapy		01:01	02:01	08:01	15:01		yes
P32	38	F	caucasian	4.9	4.38	36	no cirrhosis	no	0.4	no BLV therapy		01:01	02:01	08:01	15:01		yes
P33	56	F	caucasian	ND	4.74	156	B8	ND	2.0	no BLV therapy		02:01		07:02	15:01		yes
P34	46	M	caucasian	5.1	3.95	73	no cirrhosis	no	ND	no BLV therapy		02:01	03:01	07:02	15:01		yes
P35	32	F	caucasian	9.7	6.72	90	no cirrhosis	no	0.6	no BLV therapy		24:02		15:01	35:02		yes
P36	37	F	caucasian	5.9	4.69	33	no cirrhosis	no	0.5	no BLV therapy		01:01	02:01	08:01	15:01		yes
P37	25	M	caucasian	ND	7.72	570	no cirrhosis	no	0.7	no BLV therapy		02	68	13	15:01		yes
P38	55	F	caucasian	6.7	3.18	35	no cirrhosis	no	0.4	no BLV therapy		01:01	32:01	14:01	15:01		yes
P39	30	F	african	4.5	1.40	62	no cirrhosis	no	0.3	no BLV therapy		30:01	34:02	35:01	42:01		yes
P40	61	M	caucasian	5.4	1.00	35	no cirrhosis	no	0.9	no BLV therapy		11:01	24:02	35:01	58:01		yes

Table S2: Overlapping peptides and pools covering L-HDAg

Peptide name	Pool	Sequence	Length	Position in L-HDAg
HDV-OLP-1	pool 1	MSRSESKKNRGGREE	15	1-15
HDV-OLP-2		ESKKNRGGREEVLEQ	15	5-19
HDV-OLP-3		NRGGREEVLEQWVNG	15	9-23
HDV-OLP-4		REEVLEQWVNGRKKL	15	13-27
HDV-OLP-5	pool 2	LEQWVNGRKKLEELE	15	17-31
HDV-OLP-6		VNGRKKLEELERDLR	15	21-35
HDV-OLP-7		KKLEELERDLRKVKK	15	25-39
HDV-OLP-8		ELERDLRKVKKKIKK	15	29-43
HDV-OLP-9	pool 3	DLRKVKKKIKKLEDD	15	33-47
HDV-OLP-10*		VKKKIKKLEDDNPWL	15	37-51
HDV-OLP-11		IKKLEDDNPWLGNIK	15	41-55
HDV-OLP-12		EDDNPWLGNIKGILG	15	45-59
HDV-OLP-13	pool 4	PWLGNIKGILGKKDK	15	49-63
HDV-OLP-14		NIKGILGKKDKDGEG	15	53-67
HDV-OLP-15		ILGKKDKDGEGAPPA	15	57-71
HDV-OLP-16		KDKDGEGAPPAKRAR	15	61-75
HDV-OLP-17	pool 5	GEGAPPAKRARTDQM	15	65-79
HDV-OLP-18		PPAKRARTDQMEVDS	15	69-83
HDV-OLP-19		RARTDQMEVDSGPRK	15	73-87
HDV-OLP-20		DQMEVDSGPRKRPLR	15	77-91
HDV-OLP-21	pool 6	VDSGPRKRPLRGGFT	15	81-95
HDV-OLP-22		PRKRPLRGGFTDKER	15	85-99
HDV-OLP-23		PLRGGFTDKERQDHR	15	89-103
HDV-OLP-24		GFTDKERQDHRRRKA	15	93-107
HDV-OLP-25	pool 7	KERQDHRRRKALENK	15	97-111
HDV-OLP-26		DHRRRKALENKKKQL	15	101-115
HDV-OLP-27		RKALENKKKQLSAGG	15	105-119
HDV-OLP-28		ENKKKQLSAGGKSLS	15	109-123
HDV-OLP-29	pool 8	KQLSAGGKSLSKEEE	15	113-127
HDV-OLP-30		AGGKSLSKEEEEELK	15	117-131

HDV-OLP-31		SLSKEEEEELKRLTE	15	121-135
HDV-OLP-32		EEEEELKRLTEEDER	15	125-139
HDV-OLP-33	pool 9	ELKRLTEEDERRERR	15	129-143
HDV-OLP-34		LTEEDERRERRVAGP	15	133-147
HDV-OLP-35		DERRERRVAGPPVGG	15	137-151
HDV-OLP-36		ERRVAGPPVGGVNPL	15	141-155
HDV-OLP-37	pool 10	AGPPVGGVNPLEGGS	15	145-159
HDV-OLP-38		VGGVNPLEGGSRGAP	15	149-163
HDV-OLP-39		NPLEGGSRGAPGGGF	15	153-167
HDV-OLP-40		GGSRGAPGGGFVPSM	15	157-171
HDV-OLP-41		GAPGGGFVPSMQGVP	15	161-175
HDV-OLP-42	pool 11	GGFVPSMQGVPESPF	15	165-179
HDV-OLP-43		PSMQGVPESPFTRTG	15	169-183
HDV-OLP-44		GVPESPFTRTGEGLD	15	173-187
HDV-OLP-45		SPFTRTGEGLDIRGN	15	177-191
HDV-OLP-46		RTGEGLDIRGNQGFP	15	181-195
HDV-OLP-47	pool 12	GLDIRGNQGFPWDIL	15	185-199
HDV-OLP-48		RGNQGFPWDILFPSD	15	189-203
HDV-OLP-49		GFPWDILFPSDPPFS	15	193-207
HDV-OLP-50		DILFPSDPPFSPQSC	15	197-211
HDV-OLP-51		PSDPPFSPQSCRPQ	14	201-214

* HDV-OLP-10 data was excluded from analysis due to non-specificity for CHD patients (data not shown)

Table S3: Pre-described optimal epitopes tested in this study

HLA restriction	Position in L-HDAg	Sequence	Reference
B*07	194-202	FPWDILFPA	[4]
B*15	170-179	SMQGVPESPF	[5]
B*18	46-54	DENPWLGNI	[5], [4]
B*35	192-200	QGFPWDILF	[4]
B*35	194-202	FPWDILFPA	[4]
B*41	140-149	RERRVAGPPV	[5]
B*44	46-54	DENPWLGNI	[4]
B*51	192-200	QGFPWDILF	[6] and own data

Patients in this cohort did not express the rare HLA alleles A*02:05, B*27, B*37, B*52, or B*58. Described epitopes restricted by these HLA alleles were thus not tested.

References for Table S3:

4 Kefalakes H, Koh C, Sidney J, Amanakis G, Sette A, Heller T, *et al.* Hepatitis D virus-specific CD8+ T cells have a memory-like phenotype associated with viral immune escape in patients with chronic hepatitis D virus infection. Gastroenterology 2019;**156**:1805-19 e9.

5 Karimzadeh H, Kiraithe MM, Oberhardt V, Salimi Alizei E, Bockmann J, Schulze Zur Wiesch J, *et al.* Mutations in hepatitis D virus allow it to escape detection by CD8+ T cells and evolve at the population level. Gastroenterology 2019;**156**:1820-33.

6 Landahl J, Bockmann JH, Scheurich C, Ackermann C, Matzat V, Heide J, *et al.* Detection of a broad range of low-level major histocompatibility complex class II-restricted, hepatitis delta virus (HDV)-specific T-cell responses regardless of clinical status. J Infect Dis 2019;**219**:568-77.

Table S4: Autologous viral epitope sequences

Patient	Epitope name	Prototype sequence	Autologous viral sequence
P1	HLA-B*51: HDAg192-200	QGFPWDILF	RGFPWDMLF
P2	HLA-B*35/*51: HDAg192-200 (and olp48/49)	QGFPWDILF	RGFPWDILF
P6	HLA-B*18: HDAg46-54	DENPWLGNI	EDNPWLGNI
	HLA-B*35: HDAg192-200	QGFPWDILF	QGFPWDILF
P7	HLA-B*35: HDAg192-200	QGFPWDILF	QGFPWDILF
P8	HLA-B*35: HDAg194-202 (and olp48/49)	FPWDILFPA	FPWDILFPA
P9	HLA-B*51: HDAg194-202 (and olp48/49)	FPWDILFPA	FPWDILFPS
	olp26	DHRRRKALENKKKQL	AHRRRKALENKKKQL
P10	HLA-B*51: HDAg194-202	QGFPWDILF	QGFPWDILF
P11	HLA-B*18/*44: HDAg46-54	DENPWLGNI	DDNPWLGNI
P12	HLA-B*35: HDAg194-202 (and olp48/49)	FPWDILFPA	FPWDILFPS
P13	HLA-B*18: HDAg46-54	DENPWLGNI	EENPWLGNI
P14	HLA-B*15: HDAg170-179	SMQGVPESPF	NMLEIPESPF
	olp45	SPFTRTGEGLDIRGN	SPFSRTGEGLDVRGN
P15	olp4	REEVLEQWVNGRKKL	REETLEQWVSARKKL
	olp6	VNGRKKLEELERDLR	VSARKKLEDLERDLR
P16	olp1	MSRSESKKNRGGREE	MSRAESKKHRGGREE
	olp8	ELERDLRKVKKKIKK	ELERDLRKVKKKIKK
	olp33	ELKRLTEEDERRERR	ELERLTKEDEKRKRR
	olp43	PSMQGVPESPFTRTG	PSMQGVPESPFSRHG
P21	HLA-B*35: HDAg192-200	QGFPWDILF	QGFPWDILF
P25	HLA-B*35: HDAg194-202	QGFPWDILF	RGFPWDILF
P26	HLA-B*37: HDAg100-108	QDHRRRKAL	QEHRRRKAL
P27	HLA-B*35: HDAg194-202	QGFPWDILF	QGFPWDILF
P28	HLA-B*15: HDAg170-179	SMQGVPESPF	SMQGVPESPF
P29	HLA-B*8: HDAg50-58	WLGNIKGIL	WLGNIKGIL
P30	HLA-B*8: HDAg50-58	WLGNIKGIL	WLGNIKGIL
P31	HLA-B*8: HDAg50-58	WLGNIKGIL	WLGNIKGIL
	HLA-B*15: HDAg170-179	SMQGVPESPF	NMQGVPESPF
P32	HLA-B*8: HDAg50-58	WLGNIKGIL	WLGNIKGIL
P33	HLA-B*15: HDAg170-179	SMQGVPESPF	SMQGVPESPF
P34	HLA-B*15: HDAg170-179	SMQGVPESPF	SMQGVPESPF
P35	HLA-B*15: HDAg170-179	SMQGVPESPF	NMQGVPESPF
P36	HLA-B*15: HDAg170-179	SMQGVPESPF	NMQGVPESPF
P37	HLA-B*15: HDAg170-179	SMQGVPESPF	NMQGVPESPF
P38	HLA-B*15: HDAg170-179	SMQGVPESPF	NMQGVPESPF
P39	HLA-B*35: HDAg194-202	QGFPWDILF	QGFPWDILF
P40	HLA-B*35: HDAg194-202	QGFPWDILF	QGFPWDILF

Fig. S1

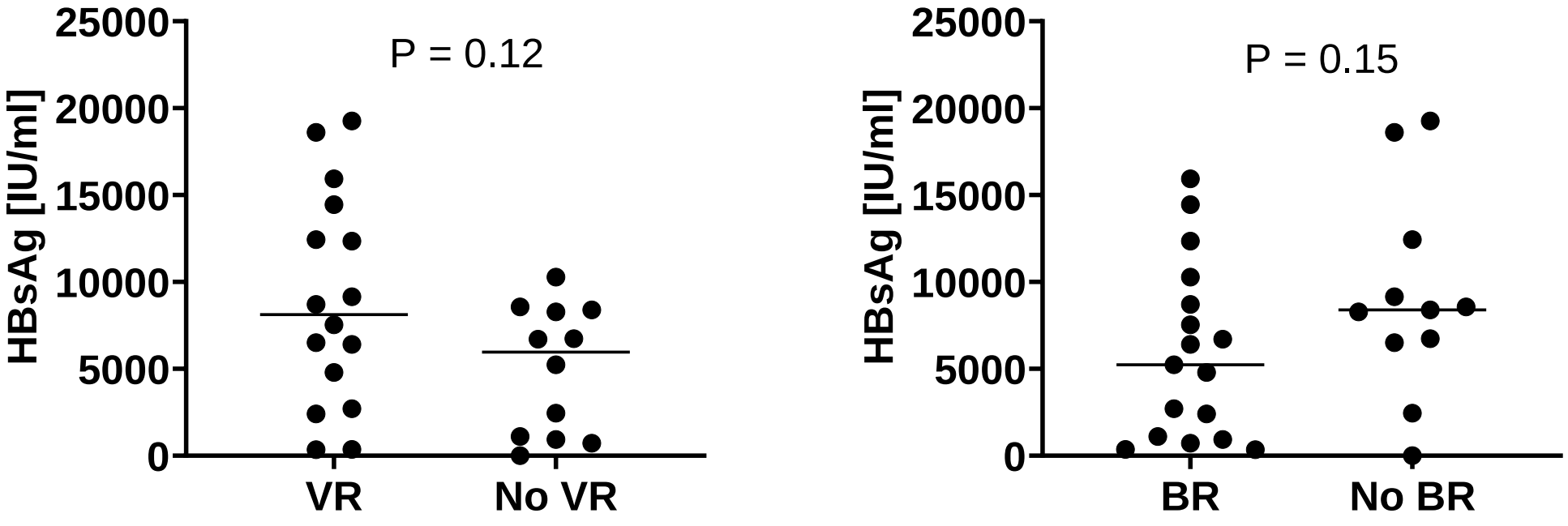

Fig. S1: Comparison of baseline HBsAg levels in patients with or without virological response (left) and in patients with or without biochemical response (right). Statistics: Mann-Whitney test.

Fig. S2

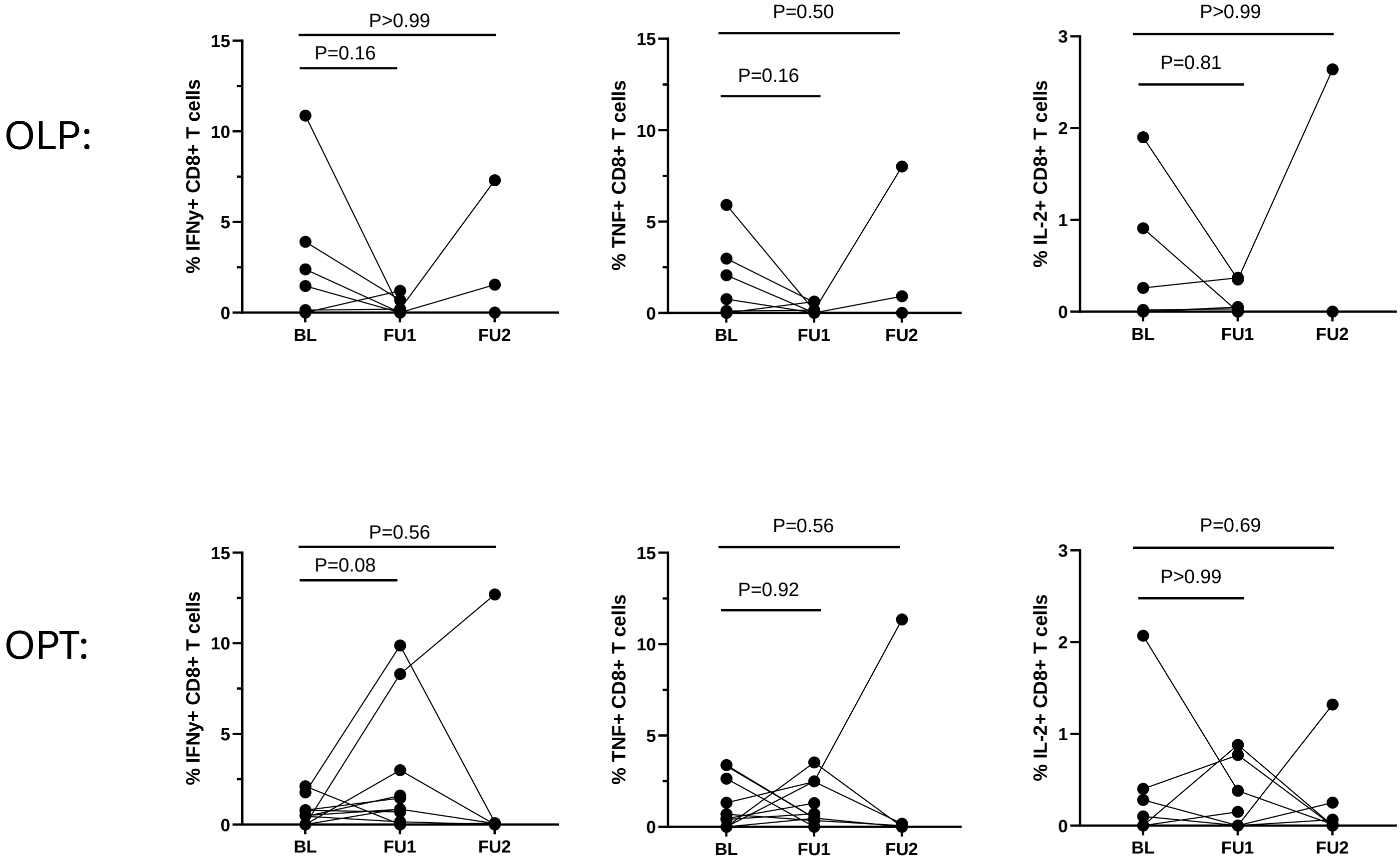

Fig. S2: Longitudinal strength of HDAg-specific CD8+ T-cell responses producing IFNγ, TNF, and IL-2. Results are displayed separately for experiments using OLP (upper row) and optimal epitope peptides (lower row), respectively. Statistics: Wilcoxon matched-pairs signed rank test.

Fig. S3

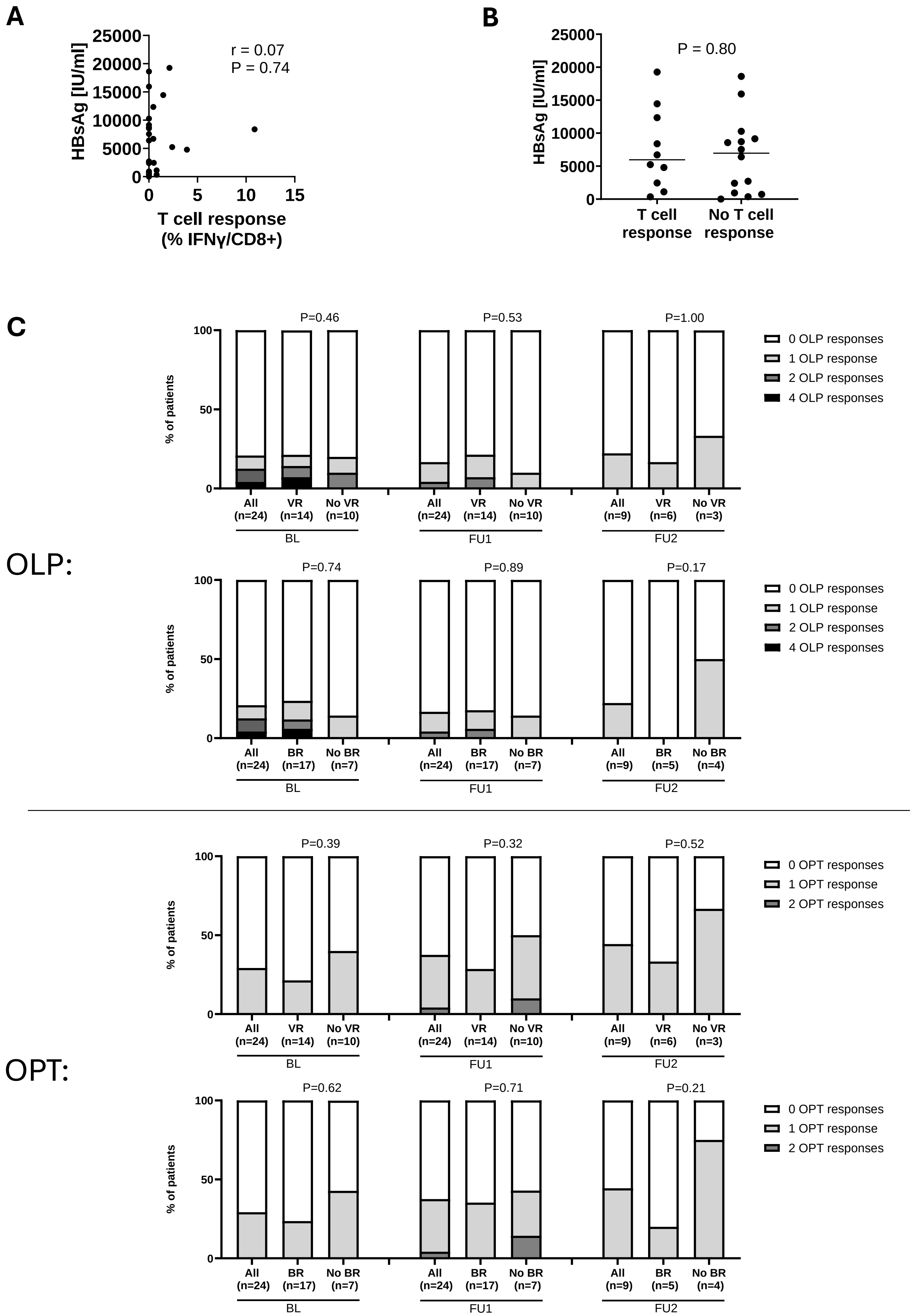
A
HBsAg [IU/ml]
25000
20000
15000
10000
5000
0
r = 0.07
P = 0.74
0
5
10
15
T cell response
(% IFNγ/CD8+)
B
HBsAg [IU/ml]
P = 0.80
T cell response
No T cell response
C
OLP:
P=0.46
P=0.53
P=1.00
% of patients
100
50
0
All (n=24)
VR (n=14)
No VR (n=10)
All (n=9)
VR (n=6)
No VR (n=3)
BL
FU1
FU2
0 OLP responses
1 OLP response
2 OLP responses
4 OLP responses
P=0.74
P=0.89
P=0.17
All (n=24)
BR (n=17)
No BR (n=7)
All (n=9)
BR (n=5)
No BR (n=4)
OPT:
P=0.39
P=0.32
P=0.52
0 OPT responses
1 OPT response
2 OPT responses
P=0.62
P=0.71
P=0.21

Fig. S3 (continued)

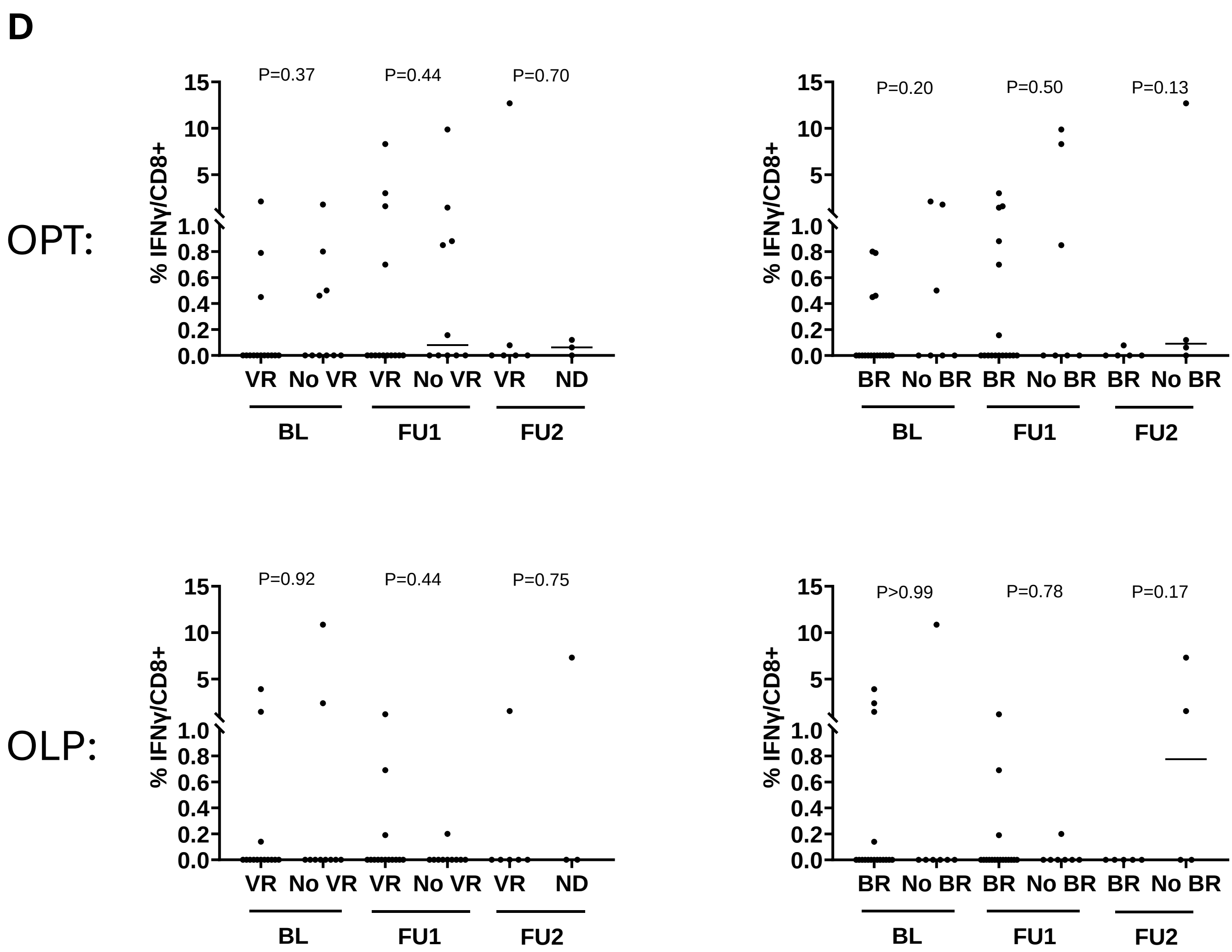

Fig. S3: Correlation of the HDAg-specific CD8+ T-cell response with baseline virological and biochemical data and treatment response.

A Presence or absence of HDAg-specific CD8+ T-cell responses was correlated with baseline HBsAg levels. **B** Strength of HDAg-specific CD8+ T-cell responses was correlated with baseline HBsAg levels. **C and D** The longitudinal number (C) and strength (D) of HDAg-specific CD8+ T-cell responses is displayed for patients with or without virological response (VR) and with or without biochemical response (BR), respectively. Responses detected using OLPs and optimal epitope peptides are displayed separately. Statistics: A, C, D: Mann-Whitney test; B: nonparametric Spearman correlation.

Fig. S4

A

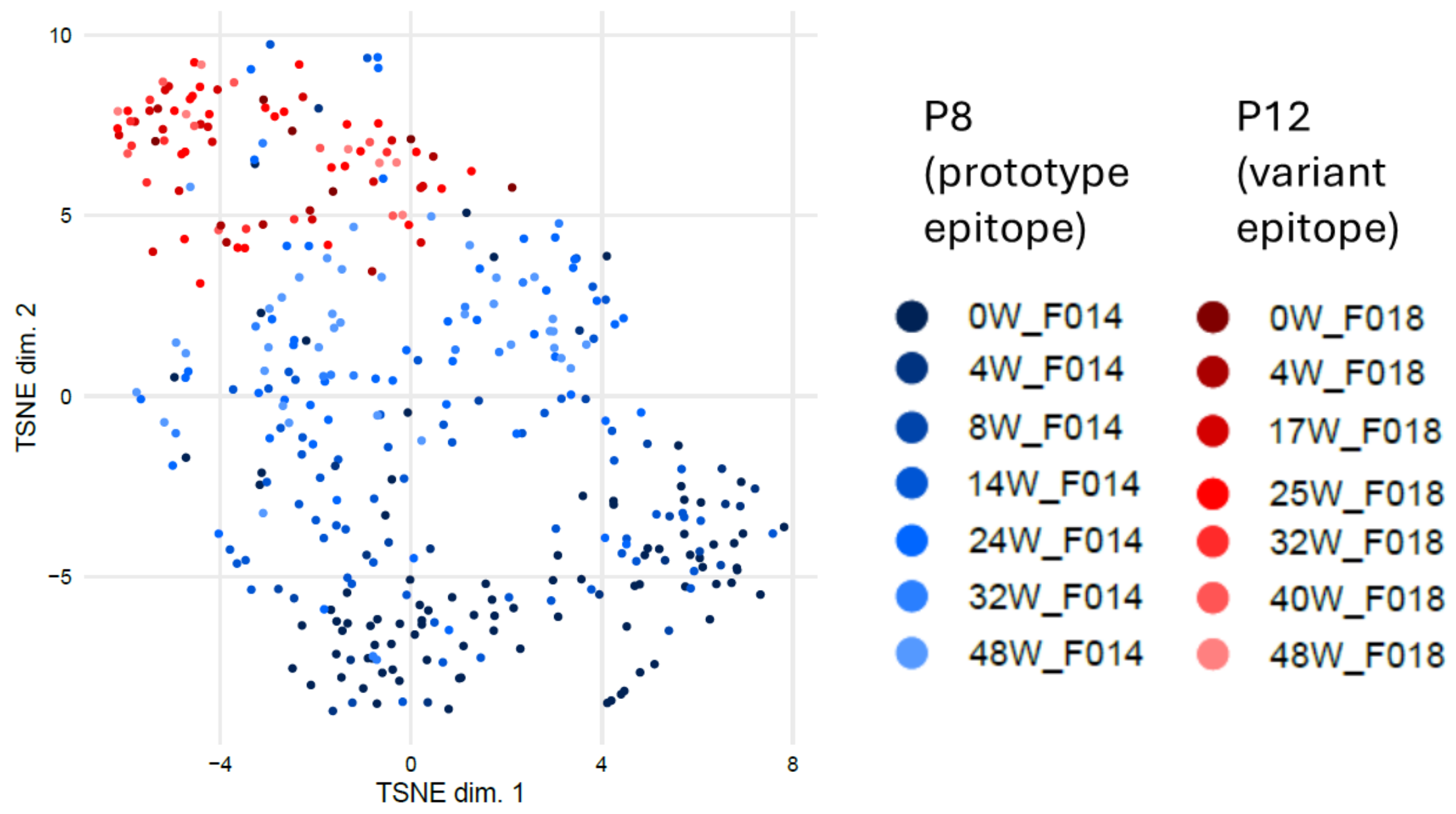

10
5
0
−5
−4
0
4
8
TSNE dim. 2
TSNE dim. 1
P8
(prototype
epitope)
P12
(variant
epitope)
0W_F014
4W_F014
8W_F014
14W_F014
24W_F014
32W_F014
48W_F014
0W_F018
4W_F018
17W_F018
25W_F018
32W_F018
40W_F018
48W_F018

B

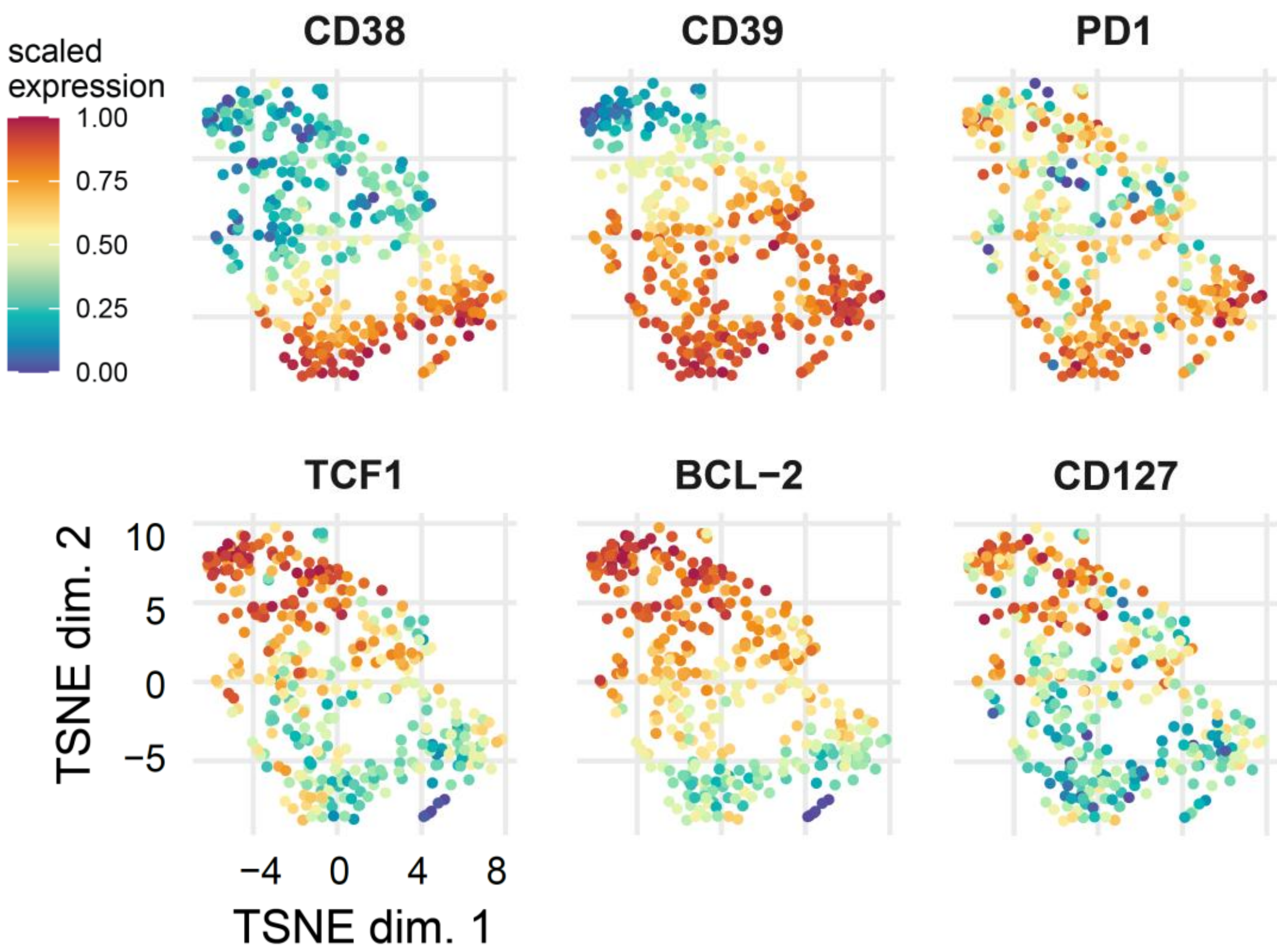

scaled
expression
1.00
0.75
0.50
0.25
0.00
CD38
CD39
PD1
TCF1
BCL−2
CD127
TSNE dim. 2
10
5
0
−5
−4
0
4
8
TSNE dim. 1

Fig. S4 (continued)

C

Marker expression on HDV-specific CD8+ T cells:

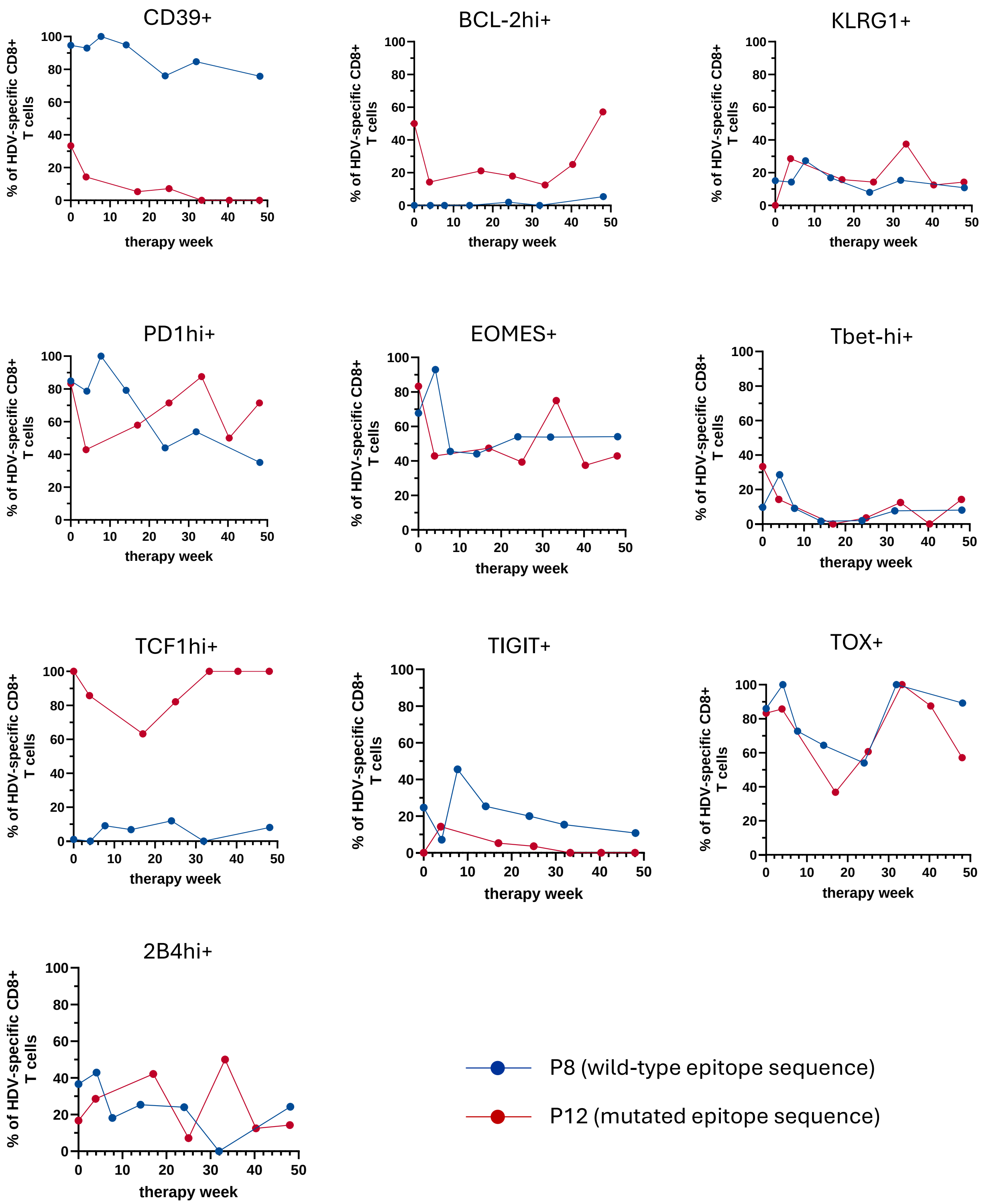

Fig. S4 (continued)

D

Marker expression on **PD1+/CD127+** HDV-specific CD8+ T cells:

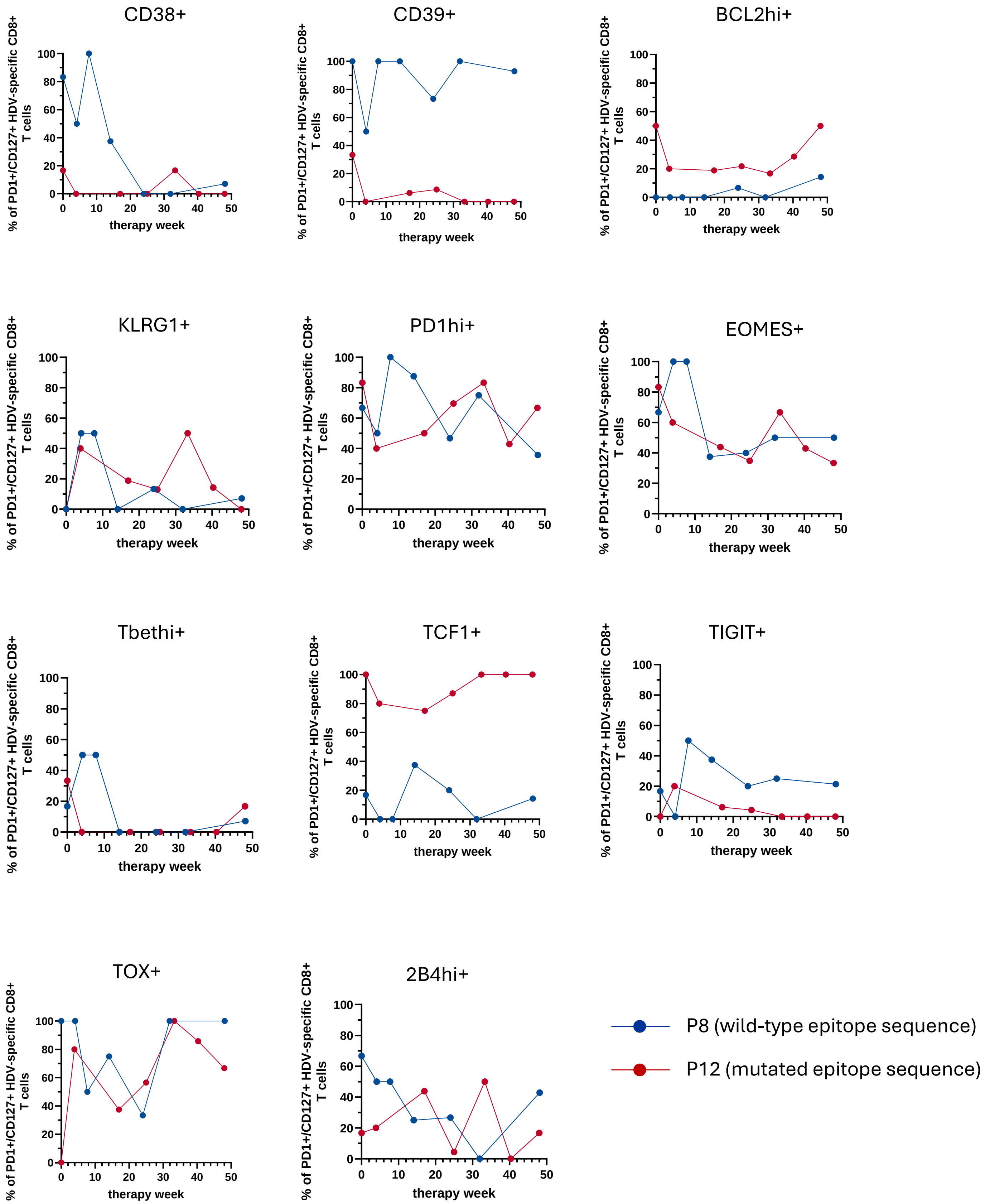

Fig. S4 (continued)

E

Marker expression on **PD1+/CD127-** HDV-specific CD8+ T cells

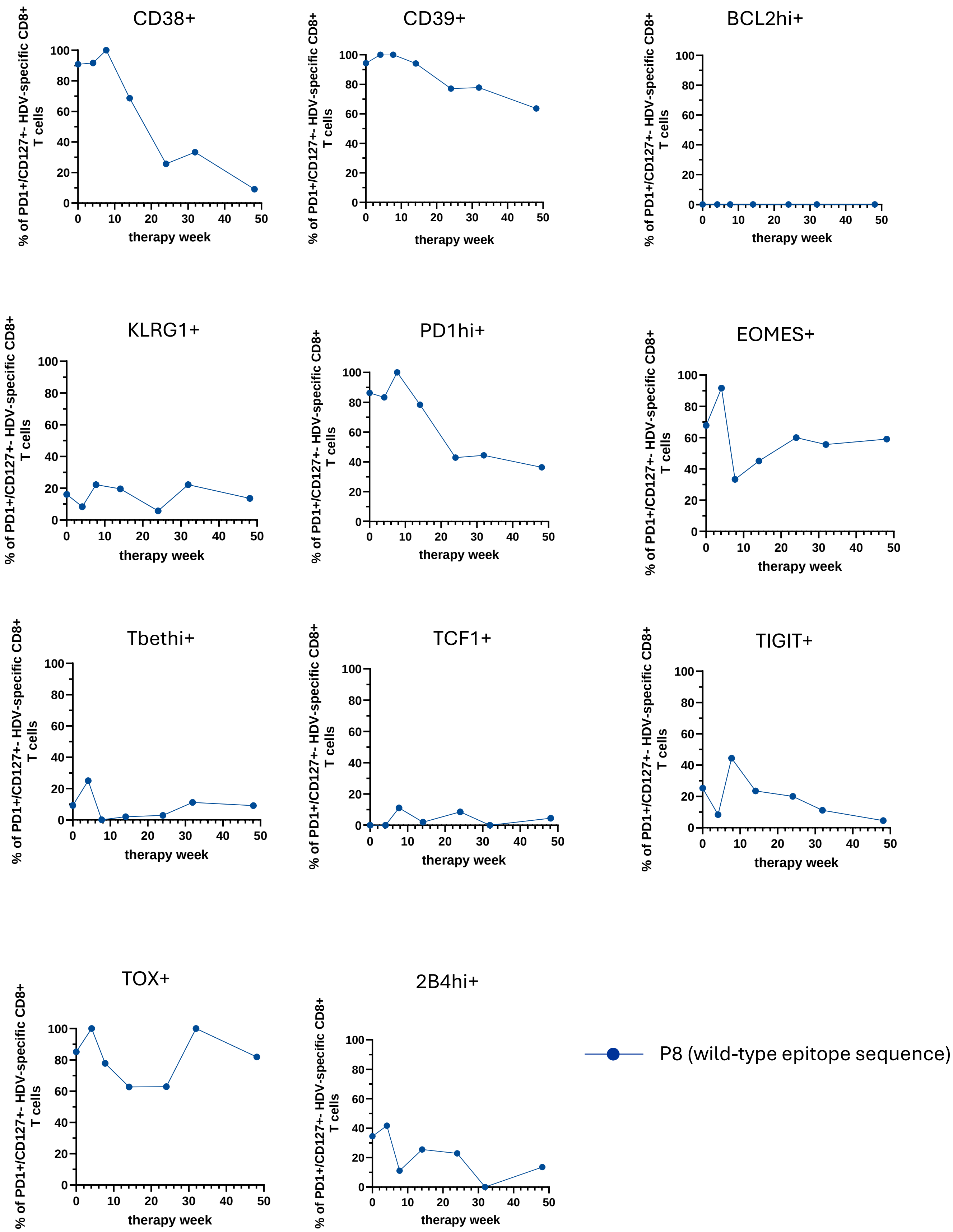

Fig. S4 (continued)

F

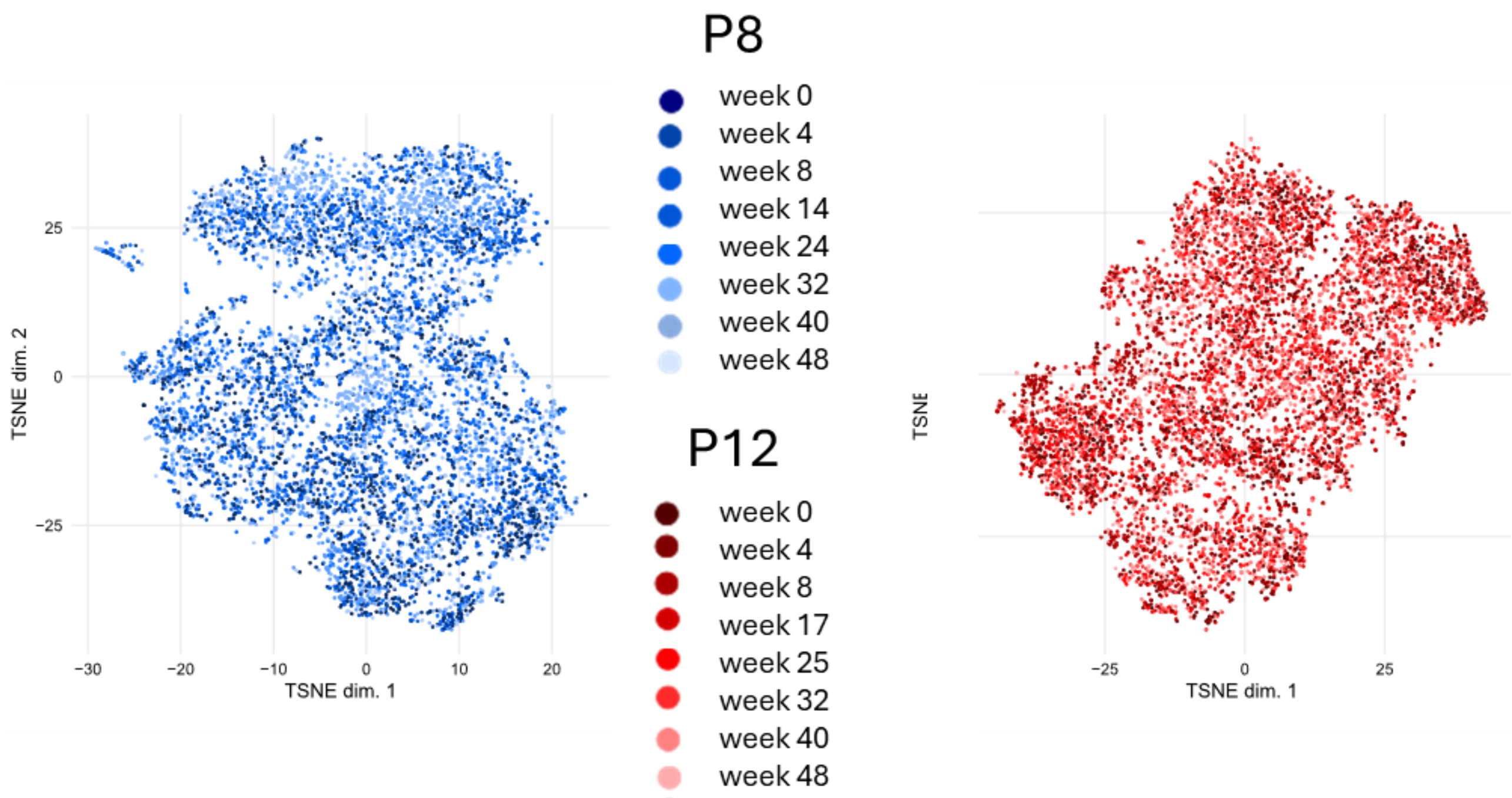

Fig. S4: BLV therapy ameliorates HDV-specific CD8+ T cells targeting epitopes with prototype viral sequence only.

A t-SNE analysis of HDAg192-200-specific CD8+ T cells from the two patients (P8 with prototype viral epitope sequence and P12 with variant viral epitope sequence) from multiple time points during 48 weeks of BLV treatment (compare Fig. 4B, where only baseline and week 48 are displayed).

B Scaled expression of single markers.

C Longitudinal percentage of marker-positive HDAg192-200-specific CD8+ T cells in patients P8 and P12.

D Longitudinal percentage of marker-positive PD1+CD127+ HDAg192-200-specific CD8+ T cells in patients P8 and P12.

E Longitudinal percentage of marker-positive PD1+CD127- HDAg192-200-specific CD8+ T cells in patients P8 (P12 is not shown, since in this patient, the number of PD1+CD127- was not sufficient for phenotypic analysis at several time-points).

F t-SNE analysis of global CD8+ T cell population from patients P8 and P12 from multiple time points during BLV treatment.

Fig. S5

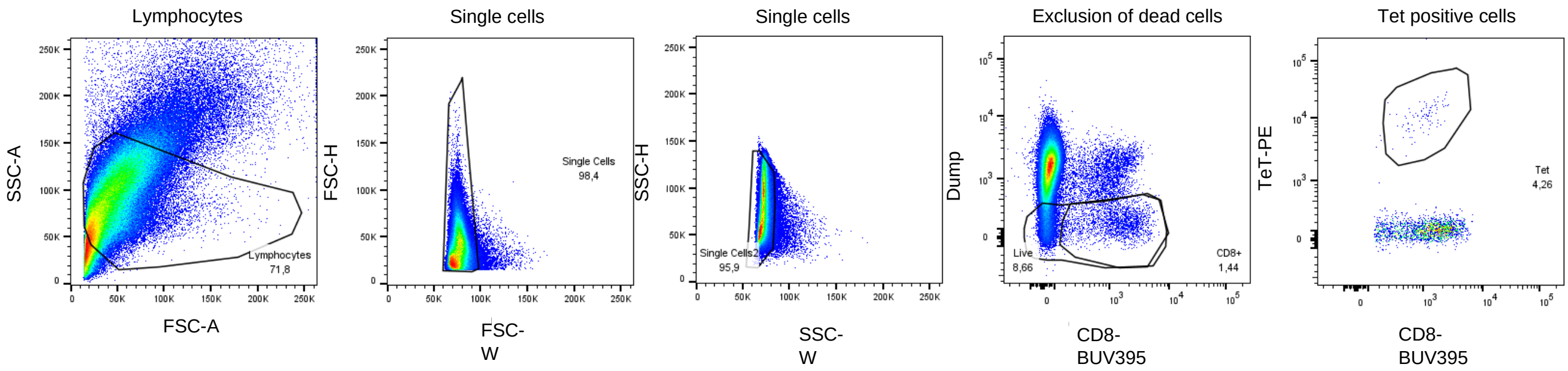

Fig. S5: Gating strategy for phenotypical analysis of tetramer-positive CD8+ T cells.
