# Supplementary material for: Fate of hepatitis D virus-specific CD8+ T cells during bulevirtide monotherapy in patients with chronic hepatitis delta
Source: JHEP Rep. 2026 Jul 24;8(10):101970. doi: 10.1016/j.jhepr.2026.101970 (PMC13594906; doi:10.1016/j.jhepr.2026.101970)
Supplement: Supplementary file 4 [file mmc2.docx]

**JHEP Reports**

**CTAT methods**

Tables for a “Complete, Transparent, Accurate and Timely account” (CTAT) are now mandatory for all revised submissions. The aim is to enhance the reproducibility of methods.

- Only include the parts relevant to your study
- Refer to the CTAT in the main text as ‘Supplementary CTAT Table’
- Do not add subheadings
- Add as many rows as needed to include all information
- Only include one item per row

**If the CTAT form is not relevant to your study, please outline the reasons why:**

1.1 **Antibodies**

| **Name** | **Citation** | **Supplier** | **Cat no.** | **Clone no.** |
| --- | --- | --- | --- | --- |
| BUV786 Anti-Human CCR7 (CD197) |  | BD Biosciences | 353230 | G043H7 |
| AlexaFluor700 Mouse Anti-Human CD38 |  | BD Horizon | 564979 | HIT2 |
| BUV737 Mouse Anti-Human CD127 (IL-7Rα) |  | BD Horizon | 612794 | HIL-7R-M21 |
| BV605 Mouse Anti-Human CD279 (PD-1) |  | BD Horizon | 563245 | EH12.1 |
| TCF1/TCF7(C63D9) Rabbit mAb (Alexa Fluor® 488 Conjugate) |  | Cell Signaling | 6444S | C63D9 |
| BUV496 Mouse Anti-Human CD45RA |  | BD OptiBuild | 750258 | HI100 |
| PE-Cyanine7 Anti-Hu/Mo T-bet |  | BD Biosciences | 25-5825-82 | eBio4B10 |
| BUV711Anti-Human KLRG1 |  | Invitrogen | 67-9488-42 | 13F12F2 |
| eFluor™ 660 Anti-HU/Mo TOX |  | BD Bioscience | 50-6502-B2 | TXRX10 |
| BV421 anti-human CD4 |  | BioLegend | 300532 | RPA-T4 |
| Fixable Viability Dye eFluor™ 780 |  | Invitrogen | 65-0865 |  |
| APC-eFluor™ 780 CD14 Anti-Human |  | I BD Bioscience | 47-0149-42 | 61D3 |
| APC-eFluor™ 780 CD19 Anti-Human |  | BD Bioscience | 47-0199-42 | HIB19 |
| BUV395 Mouse Anti-Human CD8 |  | BD Horizon | 563795 | RPA-T8 |
| APC CD8 |  | BD Bioscience | 345775 | SK1 |
| BV421 anti-BCL2 |  | BioLegend | 658709 | 100 |
| BV510 Anti-Human TIGIT |  | BioLegend | 372738 | A15153G |
| BV650 Mouse Anti-HumanCD39 |  | BD Horizon | 563361 | TU66 |
| PE-Cyanine 5.5 Anti-Human EOMES |  | BD Bioscience | 35-4877-42 | WD1928 |
| PE-CF594 Anti-Human CD244 (2B4) |  | BioLegend | 329521 | C1.7 |
| FITC Mouse Anti-Human IFN-γ |  | BD Biosciences | 340449 | 25723.11 |
| PE Rat Anti-Human IL-2 |  | BD Biosciences | 559334 | MQ1-17H12 |
| PE/Cyanine7 anti-human TNF-α Antibody |  | BioLegend | 502930 | MAb11 |
| Purified Mouse Anti-Human CD28 |  | BD Biosciences | 556620 | CD28.2 |
| rh IL-2 |  | Stemcell Technologies | 78036.2 |  |

1.2 **Cell lines**

| **Name** | **Citation** | **Supplier** | **Cat no.** | **Passage no.** | **Authentication test method** |
| --- | --- | --- | --- | --- | --- |
|  |  |  |  |  |  |

1.3 **Organisms**

| **Name** | **Citation** | **Supplier** | **Strain** | **Sex** | **Age** | **Overall n number** |
| --- | --- | --- | --- | --- | --- | --- |
|  |  |  |  |  |  |  |

1.4 **Sequence based reagents**

| **Name** | **Sequence** | **Supplier** |
| --- | --- | --- |
| **HDV-OLP-1** | MSRSESKKNRGGREE | Genaxxon bioscience GmbH |
| **HDV-OLP-2** | ESKKNRGGREEVLEQ | Genaxxon bioscience GmbH |
| **HDV-OLP-3** | NRGGREEVLEQWVNG | Genaxxon bioscience GmbH |
| **HDV-OLP-4** | REEVLEQWVNGRKKL | Genaxxon bioscience GmbH |
| **HDV-OLP-5** | LEQWVNGRKKLEELE | Genaxxon bioscience GmbH |
| **HDV-OLP-6** | VNGRKKLEELERDLR | Genaxxon bioscience GmbH |
| **HDV-OLP-7** | KKLEELERDLRKVKK | Genaxxon bioscience GmbH |
| **HDV-OLP-8** | ELERDLRKVKKKIKK | Genaxxon bioscience GmbH |
| **HDV-OLP-9** | DLRKVKKKIKKLEDD | Genaxxon bioscience GmbH |
| **HDV-OLP-10** | VKKKIKKLEDDNPWL | Genaxxon bioscience GmbH |
| **HDV-OLP-11** | IKKLEDDNPWLGNIK | Genaxxon bioscience GmbH |
| **HDV-OLP-12** | EDDNPWLGNIKGILG | Genaxxon bioscience GmbH |
| **HDV-OLP-13** | PWLGNIKGILGKKDK | Genaxxon bioscience GmbH |
| **HDV-OLP-14** | NIKGILGKKDKDGEG | Genaxxon bioscience GmbH |
| **HDV-OLP-15** | ILGKKDKDGEGAPPA | Genaxxon bioscience GmbH |
| **HDV-OLP-16** | KDKDGEGAPPAKRAR | Genaxxon bioscience GmbH |
| **HDV-OLP-17** | GEGAPPAKRARTDQM | Genaxxon bioscience GmbH |
| **HDV-OLP-18** | PPAKRARTDQMEVDS | Genaxxon bioscience GmbH |
| **HDV-OLP-19** | RARTDQMEVDSGPRK | Genaxxon bioscience GmbH |
| **HDV-OLP-20** | DQMEVDSGPRKRPLR | Genaxxon bioscience GmbH |
| **HDV-OLP-21** | VDSGPRKRPLRGGFT | Genaxxon bioscience GmbH |
| **HDV-OLP-22** | PRKRPLRGGFTDKER | Genaxxon bioscience GmbH |
| **HDV-OLP-23** | PLRGGFTDKERQDHR | Genaxxon bioscience GmbH |
| **HDV-OLP-24** | GFTDKERQDHRRRKA | Genaxxon bioscience GmbH |
| **HDV-OLP-25** | KERQDHRRRKALENK | Genaxxon bioscience GmbH |
| **HDV-OLP-26** | DHRRRKALENKKKQL | Genaxxon bioscience GmbH |
| **HDV-OLP-27** | RKALENKKKQLSAGG | Genaxxon bioscience GmbH |
| **HDV-OLP-28** | ENKKKQLSAGGKSLS | Genaxxon bioscience GmbH |
| **HDV-OLP-29** | KQLSAGGKSLSKEEE | Genaxxon bioscience GmbH |
| **HDV-OLP-30** | AGGKSLSKEEEEELK | Genaxxon bioscience GmbH |
| **HDV-OLP-31** | SLSKEEEEELKRLTE | Genaxxon bioscience GmbH |
| **HDV-OLP-32** | EEEEELKRLTEEDER | Genaxxon bioscience GmbH |
| **HDV-OLP-33** | ELKRLTEEDERRERR | Genaxxon bioscience GmbH |
| **HDV-OLP-34** | LTEEDERRERRVAGP | Genaxxon bioscience GmbH |
| **HDV-OLP-35** | DERRERRVAGPPVGG | Genaxxon bioscience GmbH |
| **HDV-OLP-36** | ERRVAGPPVGGVNPL | Genaxxon bioscience GmbH |
| **HDV-OLP-37** | AGPPVGGVNPLEGGS | Genaxxon bioscience GmbH |
| **HDV-OLP-38** | VGGVNPLEGGSRGAP | Genaxxon bioscience GmbH |
| **HDV-OLP-39** | NPLEGGSRGAPGGGF | Genaxxon bioscience GmbH |
| **HDV-OLP-40** | GGSRGAPGGGFVPSM | Genaxxon bioscience GmbH |
| **HDV-OLP-41** | GAPGGGFVPSMQGVP | Genaxxon bioscience GmbH |
| **HDV-OLP-42** | GGFVPSMQGVPESPF | Genaxxon bioscience GmbH |
| **HDV-OLP-43** | PSMQGVPESPFTRTG | Genaxxon bioscience GmbH |
| **HDV-OLP-44** | GVPESPFTRTGEGLD | Genaxxon bioscience GmbH |
| **HDV-OLP-45** | SPFTRTGEGLDIRGN | Genaxxon bioscience GmbH |
| **HDV-OLP-46** | RTGEGLDIRGNQGFP | Genaxxon bioscience GmbH |
| **HDV-OLP-47** | GLDIRGNQGFPWDIL | Genaxxon bioscience GmbH |
| **HDV-OLP-48** | RGNQGFPWDILFPSD | Genaxxon bioscience GmbH |
| **HDV-OLP-49** | GFPWDILFPSDPPFS | Genaxxon bioscience GmbH |
| **HDV-OLP-50** | DILFPSDPPFSPQSC | Genaxxon bioscience GmbH |
| **HDV-OLP-51** | PSDPPFSPQSCRPQ | Genaxxon bioscience GmbH |
| **HDV-OPT-194-202** | FPWDILFPA | Genaxxon bioscience GmbH |
| **HDV-OPT-170-179** | SMQGVPESPF | Genaxxon bioscience GmbH |
| **HDV-OPT-46-54** | DENPWLGNI | Genaxxon bioscience GmbH |
| **HDV-OPT-192-200** | QGFPWDILF | Genaxxon bioscience GmbH |
| **HDV-OPT-194-202** | FPWDILFPA | Genaxxon bioscience GmbH |
| **HDV-OPT-140-149** | RERRVAGPPV | Genaxxon bioscience GmbH |

1.5 **Biological samples**

| **Description** | **Source** | **Identifier** |
| --- | --- | --- |
|  |  |  |

1.6 **Deposited data**

| **Name of repository** | **Identifier** | **Link** |
| --- | --- | --- |
|  |  |  |

1.7 **Software**

| **Software name** | **Manufacturer** | **Version** |
| --- | --- | --- |
| **FlowJo** | LLC, BD Life Sciences, USA | 10.6.2 |
| **GraphPad Prism** | GraphPad Prism Software Inc., USA | 10 |
| **R & R-Studio** |  | 4.5.1 |

1.8 **Other (e.g. drugs, proteins, vectors etc.)**

| **Name** | **Supplier** | **Cat no.** |
| --- | --- | --- |
| Anti-PE MicroBeads | Miltenyi Biotech | 130-048-801 |

1.9 **Please provide the details of the corresponding methods author for the manuscript:**

Prof. Dr. Christoph Neumann-Haefelin

University Hospital Cologne

Department of Gastroenterology and Hepatology

Kerpener Strasse 62

50937 Köln

Germany

christoph.neumann-haefelin@uk-koeln.de

**2.0	Please confirm for randomised controlled trials all versions of the clinical protocol are included in the submission. These will be published online as supplementary information.**
